# Extensive Diversity and Disparity of the Early Miocene Platanistoids (Cetacea, Odontoceti) in the Southeastern Pacific (Chilcatay Formation, Peru)

**DOI:** 10.3390/life10030027

**Published:** 2020-03-18

**Authors:** Giovanni Bianucci, Christian de Muizon, Mario Urbina, Olivier Lambert

**Affiliations:** 1 Dipartimento di Scienze della Terra, Università di Pisa, 56126 Pisa, Italy; 2 CR2P (CNRS, MNHN, SU), Muséum National d’Histoire Naturelle, Département Origines et Évolution, 75005 Paris, France; muizon@mnhn.fr; 3 Departamento de Paleontología de Vertebrados, Museo de Historia Natural de la Universidad Nacional Mayor de San Marcos, Lima 15072, Peru; mariourbina01@hotmail.com; 4 Institut Royal des Sciences Naturelles de Belgique, D.O. Terre et Histoire de la Vie, 1000 Brussels, Belgium

**Keywords:** Odontoceti, Squalodelphinidae, Platanistidae, early Miocene, Peru, phylogeny, paleoecology

## Abstract

Several aspects of the fascinating evolutionary history of toothed and baleen whales (Cetacea) are still to be clarified due to the fragmentation and discontinuity (in space and time) of the fossil record. Here we open a window on the past, describing a part of the extraordinary cetacean fossil assemblage deposited in a restricted interval of time (19–18 Ma) in the Chilcatay Formation (Peru). All the fossils here examined belong to the Platanistoidea clade as here redefined, a toothed whale group nowadays represented only by the Asian river dolphin *Platanista gangetica*. Two new genera and species, the hyper-longirostrine *Ensidelphis riveroi* and the squalodelphinid *Furcacetus flexirostrum*, are described together with new material referred to the squalodelphinid *Notocetus vanbenedeni* and fragmentary remains showing affinities with the platanistid *Araeodelphis*. Our cladistic analysis defines the new clade Platanidelphidi, sister-group to Allodelphinidae and including *E. riveroi* and the clade Squalodelphinidae + Platanistidae. The fossils here examined further confirm the high diversity and disparity of platanistoids during the early Miocene. Finally, morphofunctional considerations on the entire platanistoid assemblage of the Chilcatay Formation suggest a high trophic partitioning of this peculiar cetacean paleocommunity.

## 1. Introduction

The evolutionary history of cetaceans is overall increasingly well documented by a representative fossil record scattered in various parts of the world [1,2,3]. This record describes in detail: (1) The progressive adaptation of ancient cetaceans, named archaeocetes, to life in the sea [4,5,6]; (2) the origin of mysticetes and their later evolution characterized by the replacement of teeth with baleen [7,8,9,10] and the tendency for extreme gigantism [11,12]; and (3) the great radiation of odontocetes that over time explored a large number of feeding strategies and ecological niches, thanks to their ability to echolocate [13,14,15] and to their marked cranial plasticity [16,17]. In spite of this general picture, our knowledge on this highly successful clade of marine mammals is still far from exhaustive. In recent years, new taxa have been continuously described, highlighting the fact that we do not yet possess a solid dataset of past cetacean diversity. Furthermore, two weak points in the framework of the evolutionary history of cetaceans are (1) the discontinuity of the fossil record, from both a temporal and a geographical point of view; and (2) the low geochronological resolution featuring many fossils or fossil assemblages. These critical issues should be taken into consideration when attempting to reconstruct in detail the ecological structure of ancient cetacean paleocommunities, to analyze with a statistically significant approach some evolutionary trends, and to tentatively correlate these to the main abiotic and biotic changes observed at a global scale [7,18,19]. 

**Figure 1:**
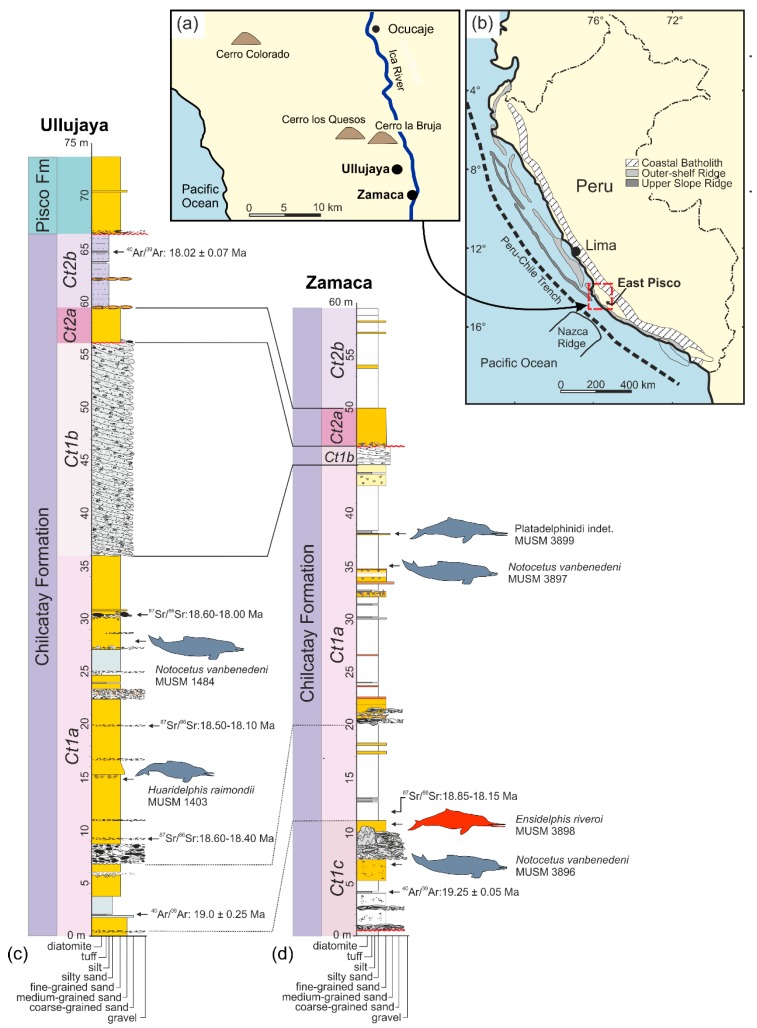
Geographical position of Ullujaya and Zamaca (Pisco Basin, southern coast of Peru) (**a**,**b**) and related composite stratigraphic sections (**c**,**d**) showing the distribution of fossil platanistoids in the Chilcatay Fm, including the specimens with known stratigraphical position described in this paper. Red silhouette indicates holotype. Absolute datings (^40^Ar/^39^Ar on ash layers) constraining the age of the fossil platanistoids are also reported along the sections.

In this context, this paper focuses on a part of the fossil cetacean assemblage coming from the extraordinary Cenozoic marine vertebrate Lagerstätte of the East Pisco Basin (Peru). The fossils here examined were collected in the lower Miocene layers of the Chilcatay Formation (Chilcatay Fm) exposed in the vertebrate-bearing fossil localities of Ullujaya and Zamaca, western Ica Valley, Ica Region. More than 180 partial skeletons of cetaceans, together with remains of other vertebrates, have been discovered in these two localities and most of these fossils are marked on two published geological maps [20,21,22]. Furthermore, all the fossils reported in the maps have been included in detailed stratigraphic columns accompanied by a precisely defined geochronological and biostratigraphic framework (ca 19–18 Ma) for the deposition of the entire sequence of fossil-bearing marine sediments (Figure 1). 

More specifically, this paper focuses on the fossils of the Ullujaya-Zamaca assemblage belonging to the superfamily Platanistoidea, an odontocete clade that underwent a major radiation during the early Miocene and which today is only represented by the South Asian river dolphin (*Platanista gangetica*) confined to the freshwaters of the Indus and Ganges river systems [23]. Following other works already published by us on the platanistoids from the Chilcatay Formation [24,25,26], the fossils here described further support the great diversity and morphological disparity of this clade. 

## 2. Materials and Methods 

### 2.1. Institutional Abbreviations

GAS, Georgian Academy of Sciences, Tbilisi, Georgia; GMNH, Gunma Museum of Natural History, Tomioka, Japan; IRSNB, Institut Royal des Sciences Naturelles de Belgique, Brussels, Belgium; LACM, Natural History Museum of Los Angeles County, Los Angeles, U.S.A.; LDUCZ, Grant Museum of Zoology, University College London, United Kingdom; MGP-PD, Museo di Geologia e Paleontologia, Università di Padova; MNHN, Muséum National d’Histoire Naturelle, Paris, France; MLP, Museo de Ciencias Naturales de La Plata, Buenos Aires, Argentina; MSNUP, Museo di Storia Naturale, Università di Pisa, Italy; MUSM, Museo de Historia Natural, Universidad Nacional Mayor de San Marco, Lima, Peru; MZUF, Museo di Storia Naturale, Zoological collection, Università degli Studi di Firenze, Italy; NMG, National Museum of Georgia, Tbilisi, Georgia; NMV, Museum Victoria Palaeontology Collections, Melbourne, Australia; OU, Geological Museum, University of Otago, Dunedin, New Zealand; SBCM, San Bernardino County Museum, Redlands, U.S.A.; UCMP, University of California Museum of Paleontology, Berkeley, U.S.A.; USNM, National Museum of Natural History, Smithsonian Institution, Washington, D.C., U.S.A.

### 2.2. Anatomical Abbreviation

BZW, bizygomatic width of the skull; CBL, condylobasal length of the skull; TBL, total body length.

### 2.3. Collection and Preparation 

The platanistoid specimens described here where discovered during several field expeditions from 2010 to 2019 that involved all the authors of this paper. The fossils were excavated by one of the authors (M.U.) and by W. Aguirre and subsequently transported to the MUSM for preparation and storage. The preparation and consolidation of these fossils was made by W. Aguirre using mechanical tools and standard fossil vertebrate preparation techniques.

### 2.4. Anatomical Terminology 

The anatomical terminology follows Mead and Fordyce [27] for the skull and mostly Evans and de Lahunta [28] for the postcranial skeleton. 

### 2.5. Cladistic Analysis 

The phylogenetic analysis was performed using a modified version of the matrix published by Bianucci et al. [26] (Table A1 in the Appendix B). The new genera *Ensidelphis* and *Furcacetus* and seven new characters (characters 42–48 in the Appendix A) were added, whereas the fragmentary USNM 475496 specimen and one controversial character (character 42 in [26]) were removed from the matrix. Some character states were coded differently from a previous version of the matrix due to the discovery of new material (e.g., for *Notocetus*) or a better preparation of previously described fossils (e.g., MUSM 603). The final matrix includes 24 taxa coded for 48 morphological characters. 

The parsimony analysis was executed with the software PAUP (version 4.0b10; [29]), considering all characters unordered and unweighted, and using the tree bisection and reconnection (TBR) algorithm with ACCTRAN optimization. 

## 3. Geological, Stratigraphical, and Paleontological Setting 

The marine sediments constituting the Chilcatay Fm as exposed in the vertebrate-bearing fossil localities of Ullujaya and Zamaca were deposited in the onshore portion of the East Pisco Basin, which extends over some 30 km across strike between the present-day Coastal Cordillera to the west and the Western Cordillera to the east, and about 200 km from Pisco to Nazca (Figure 1) [30,31,32]. The complete Eocene–Pliocene succession filling this basin overlies a pre-Cenozoic crystalline basement consisting of a variety of Precambrian metamorphic rocks, known as the Arequipa Massif [33,34], intruded by a complex assemblage of lower Paleozoic gabbroic to granitoid rocks forming the San Nicolás Batholith [35] that, in turn, is unconformably overlain by Jurassic volcano-sedimentary rocks [32]. From its base upward the Cenozoic fill of the basin comprises the Caballas, Paracas, Otuma, Chilcatay, and Pisco formations [21,22,31,36,37,38,39]. 

On the whole the Eocene–Pliocene sedimentary succession of the East Pisco Basin represents one of the most significant marine vertebrate Lagerstätte of the Cenozoic Era due to the exceptional preservation and the elevated concentration of fossils [21,22,40,41,42,43,44,45,46] referred to cetaceans (archaeocetes [47,48,49]; odontocetes [24,25,50,51,52,53,54,55,56,57,58,59,60,61,62]; mysticetes [10,63,64,65,66,67,68]), pinnipeds [69,70], marine birds [71,72,73,74], marine turtles [75], marine sloths [76,77,78,79,80,81], and sharks and rays [82,83,84,85,86,87,88]. 

By using an allostratigraphic approach, Di Celma et al. [21,22] subdivided the Chilcatay strata exposed in the vertebrate-bearing fossil localities of Ullujaya and Zamaca into two distinctive sediment wedges, informally designated Ct1 and Ct2 in ascending stratigraphic order, separated by a major intraformational unconformity (Figure 1). In the Zamaca area the base of Ct1 rests with an angular unconformity on the Otuma Formation. This basal unconformity does not occur in the Ullujaya area, where the lowermost portion of the Chilcatay Fm is not exposed. The Ct1 allomember comprises three facies associations indicative of shoreface (Ct1c), offshore (Ct1a), and subaqueous delta (Ct1b) depositional settings. The Ct1c association, only exposed at the Zamaca locality, is 10.5 m-thick and consists of massive or weakly bedded sandstones with scattered boulders alternating with pebble- to boulder-sized conglomerate beds with abundant shelly calcarenite matrix. The Ct1a association is 35 m and 31 m-thick at Ullujaya and Zamaca, respectively, and consists of silty to sandy mudstones interbedded with occasional very fine- to fine-grained sandstone beds, as well as a few volcanic ash layers. The Ct1b association is comprised of mixed siliciclastic-carbonate oblique and sigmoidal clinoforms downlapping onto the underlying sediments of Ct1a; Ct1b thickness decreases basinward from about 20 m at Ullujaya to a zero-edge in the central part of the Zamaca area. The Ct2 allomember comprises two facies associations recording shoreface (Ct2a) and offshore (Ct2b) marine depositional settings. The Ct2a association is about 3.5 m-thick at both Ullujaya and Zamaca and is composed of medium- to very coarse-grained sandstones containing sub-rounded to sub-angular pebbles. The Ct2b association is 7 m and more than 15 m-thick at Ullujaya and Zamaca, respectively, and consists of a heterolithic succession of weakly bioturbated, thinly-bedded silty mudstone intercalated with minor, laterally persistent, very fine-grained sandstone interbeds and occasional volcanic ash layers [21,22].

The entire stratigraphical succession of the Chilcatay Fm exposed at the Zamaca and Ullujaya localities has been roughly constricted through radiometric dating of ash layers to an interval between 19.25 and 18.02 Ma (late early Miocene, Burdigalian), considering that a volcanic ash layer sampled at Zamaca, 4 m above the contact between the Chilcatay Fm and the underlying Otuma Formation, gave an ^40^Ar/^39^Ar age of 19.25 ± 0.05 Ma, and that a volcanic ash layer sampled at Ullujaya, just 1 m below the contact between the Chilcatay and overlying Pisco Formation, provide an age of 18.02 ± 0.07 Ma [89]. Moreover, four samples for ^87^Sr/^86^Sr stratigraphy were collected along the Ct1a sequence at Zamaca and Ullujaya and gave ages for the whole stratigraphical sequence comprised between 18.85 and 18.00 Ma [90]. These ^40^Ar/^39^Ar and ^87^Sr/^86^Sr geochronological ages are consistent with biostratigraphic results obtained with silicoflagellates and diatoms, both further constraining the deposition of the Chilcatay Fm in the Ullujaya-Zamaca area between 19 and 18 Ma [21,60]. Another volcanic ash sample, collected from the basal portion of the Ct1a facies association exposed at Ullujaya, gave a ^40^Ar/^39^Ar ages of 19.00 ± 0.28; consequently, the age of the underlying Ct1c facies association exposed at Zamaca can be further constricted between 19.25 ± 0.08 Ma and 19.00 ± 0.28 Ma.

The vertebrate fossil assemblage of the Chilcatay Fm exposed at Ullujaya and Zamaca is dominated by cetaceans (mostly odontocetes) and elasmobranches (mostly lamniformes and carcharhiniformes); large bony fishes and sea turtles were also recorded [20,21,22,86]. Besides the platanistoid remains here described, the odontocete assemblage includes already published material belonging to the squalodelphinids *Huaridelphis raimondii* [24] and *Notocetus vanbenedeni* [25], the longirostrine homodont *Chilcacetus cavirhinus* [57], the heterodont *Inticetus vertzi* [60], and undescribed eurhinodelphinids, kentriodontids, and physeteroids [20,21,22].

## 4. Systematic Paleontology

Cetacea Brisson, 1762

Neoceti Fordyce and Muizon, 2001

Odontoceti Flower, 1867

Platanistoidea Gray, 1863

**Remarks on the superfamily Platanistoidea and its content.** In its first definition proposed by Simpson [91] the superfamily Platanistoidea included *Platanista*, all other extant "river dolphins" (*Lipotes*, *Inia*, and *Pontoporia*, the latter being listed there as *Stenodelphis*), and their closest fossil relatives. Currently, the only extant genus recognized as belonging to this clade is *Platanista*, whereas the other "river dolphins" are placed within the Delphinida clade [92,93,94,95,96,97]. However, in the past decades a number of fossil taxa have been included in the Platanistoidea, radically changing the concept of this superfamily. Besides the Platanistidae, Muizon [92] first included in the Platanistoidea the extinct families Squalodelphinidae and Squalodontidae, and, a few years later [98], also the Dalpiazinidae and *Prosqualodon*. Later, Fordyce [99] added the Waipatiidae and Barnes [100] the Allodelphinidae. This broad concept of the Platanistoidea has been questioned in several recent phylogenies. For example, the heterodont Squalodontidae and *Prosqualodon* were recovered in a more basal position in several analyses [26,95,101,102], whereas in other analyses the position of these taxa inside or outside Platanistoidea depends on the settings of the phylogenetic analyses (e.g., homoplastic characters being down weighted or not [103,104,105,106,107]). The family Waipatidae was also removed from Platanistoidea in some phylogenies (e.g., [26,95,101,102]), whereas the poorly known Dalpiazinidae were never included in a software-assisted phylogenetic analysis. By contrast, the families Allodelphinidae and Squalodelphinidae appear as two distinct clades closely related to the Platanistidae in part of the recent phylogenies (e.g., [24,26,101,108]), with allodelphinids being recovered in the basalmost position, sister group of the clade formed by the platanistids and squalodelphinids. In several other recent papers (e.g., [103,104,105,106,107]) allodelphinids were not included in the phylogenetic analyses and squalodelphinids were paraphyletic.

Since the phylogenetic analysis presented below confirms again the close relationships between the Platanistidae, Squalodelphinidae, and Allodelphinidae, a new definition of the Platanistoidea sensu stricto is proposed below, only including the above mentioned three families and thus excluding the Dalpiazinidae, Squalodontidae, Waipatidae, and *Prosqualodon*. 

Proposing such a less inclusive, but more stable definition of this superfamily, we do not a priori exclude that in future analyses the Platanistoidea clade falls near to one or more of the above excluded families. In fact, the aim of this restrictive choice is essentially to put order in the controversial systematics of the large odontocete group including extinct platanistoid-like taxa. 

Finally, the New Clade Name (NCN) Platanidelphidi is below defined following the rules reported in the International Code of Phylogenetic Nomenclature (PhyloCode [109]). The Platanidelphidi clade includes the new genus *Ensidelphis* described below and the platanistoid MUSM 603 previously referred to aff. *Huaridelphis raimondii* [24], together with the clade formed by Platanistidae + Squalodelphinidae, the latter being repeatedly recovered in morphological phylogenies since the first analyses by Muizon [92,98].

**Emended diagnosis of Platanistoidea.** The members of the Platanistoidea are nearly homodont odontocetes having single-rooted teeth and sharing the following characters: (1) Vertex distinctly shifted to the left compared to the sagittal plane of the skull (absent in *Allodelphis* and *Ninjadelphis*); (2) long hamular fossa of the pterygoid sinus extending anteriorly on the palatal surface of the rostrum (also present in Ziphiidae); (3) presence of an articular rim on the lateral surface of the periotic; (4) elongated anterior spine on the tympanic bulla, associated with a marked anterolateral convexity; (5) great reduction of the coracoid process of the scapula and the acromion located on the anterior edge of the scapula (also present in *Squalodon* and *Prosqualodon*); (6) neurocranium distinctly shorter than wide, with ratio between neurocranium length (longitudinal, from occipital condyles to level of antorbital notches) and postorbital width < 0.90 (also present in Eurhinodelphinidae); and (7) anterior portion of the zygomatic process of the squamosal in contact with the postorbital process of the frontal (also present in Eurhinodelphinidae).

**Platanidelphidi** **(NCN)**

**Definition.** The branch-based Platanidelphidi consists of the extant *Platanista* and all species that share a more recent common ancestor with *Platanista* than with *Allodelphis*. 

**Etymology.** From *Platanista*, the type genus of the Platanistidae; and from *delphinus*, dolphin in Latin. The name is also a combination of Platanistidae and Squalodelphinidae, the two families currently included in the Platanidelphidi.

**Diagnosis.** The members of the Platanidelphidi clade are platanistoids sharing the following synapomorphies: (1) Asymmetry of the premaxillae on the rostrum at some distance anterior to the premaxillary foramina, with the right premaxilla being distinctly narrower than the left in dorsal view; (2) posterior dorsal infraorbital foramen(ina) along the vertex more medial than the lateralmost margin of the premaxilla on the cranium; (3) deep fossa in the frontal on orbit roof, at the level of the frontal groove (presumably for orbital lobe of pterygoid sinus); (4) palatines not contacting each other on the sagittal plane and displaced dorsolaterally; (5) thick zygomatic process of the squamosal (ratio between the maximum distance from the anteroventral margin of the zygomatic process to the posterodorsal margin, in lateral view, and the vertical distance from the lower margin of the occipital condyles to the vertex of the skull > 0.35); (6) dorsal outline of the zygomatic process of the squamosal in lateral view being dorsally convex (also present in *Squalodon* and in some specimens of the eurhinodelphinid *Xiphiacetus*); and (7) ventral edge of the zygomatic process of squamosal in lateral view being almost straight or convex. 

Platanidelphidi *incertae sedis*

***Ensidelphis***, **gen. nov.**

**LSID**: zoobank.org:act: C0D8D0CE-1769-4BA1-85D9-054889976B18

**Type and only known species**. *Ensidelphis riveroi*, sp. nov.

**Diagnosis**. As for the type and only referred species.

**Etymology**. From ‘*ensis’*, Latin name of a Roman sword similar to the *gladius* but longer and narrower; for the very elongated and narrow rostrum; and from ‘*delphis*’, dolphin in Latin. Gender masculine.

***Ensidelphis riveroi***, **sp. nov.**

Figure 2, Figure 3, Figure 4, Figure 5, Figure 6, Figure 7, Figure 8 and Figure 9, Table 1 and Table 2

**LSID**: zoobank.org:act: act:1DEC0DD9-FEFA-4CC8-A668-11934B4256AA

**Holotype and only referred specimen**. MUSM 3898 consists of an almost complete and well-preserved cranium (only small portions of both antorbital processes and both the jugals are missing) with articulated, complete mandibles. Both tympanic bullae are exposed on the ventral surface, probably hiding the in situ periotics and accessory ossicles. Only six incomplete teeth are visible, embedded in their alveoli on the right mandible. The atlas, axis, and two additional cervical vertebrae from the same animal are also preserved.

**Type locality.** Zamaca locality, Western Ica Valley, Ica Region, Southern Peru (Figure 1a,b). Geographic coordinates: 14°37'1.65" S, 75°37'31.25" W; 307 m above sea level. This specimen was reported in the Zamaca fossil map of Di Celma et al. [22] with the field number ZM 97 and provisionally referred to “aff. Platanistidae indet.”

**Type horizon**. The holotype of *Ensidelphis riveroi* MUSM 3898 was collected in the Chilcatay Fm exposed at Zamaca locality, and more precisely at 10.7 m above the contact with the underlying Otuma Formation, near the top of the Ct1c facies association of the Ct1 allomember [21,22] (Figure 1d). The age of the Ct1c facies association is constricted between 19.25 ± 0.08 Ma and 19.00 ± 0.28 Ma (early Burdigalian) on the basis of two volcanic ash layer samples dated by ^40^Ar/^39^Ar [89]. 

**Diagnosis**. *Ensidelphis* is a small odontocete having a narrow and very elongated rostrum (about 80% of the CBL) bearing about 64 small single-rooted teeth in each quadrant. It differs from all other odontocetes in having a protuberance (‘temporal swelling’, new term) on the lateral surface of the temporal fossa dorsal to the squamosal-parietal suture. It is referred to the Platanistoidea as defined above by having: Elongated hamular fossa of the pterygoid sinus extending anteriorly on the palatal surface of the rostrum; neurocranium shorter than wide (ratio < 0.90); and elongated anterior spine on the tympanic bulla associated with a marked anterolateral convexity. It differs from all other Platanistoidea in having a lesser posterior extension of both right and left ascending processes of the premaxillae, ending at the contact with the anterolateral angles of the nasals, and in having an even more elongated anterior spine of the tympanic bulla. It differs from all other Platanistoidea, with the exception of *Platanista*, for the lesser minimal distance between the temporal crests (see quantification below; character possibly related to the temporal swelling mentioned above). It differs from the similarly hyper-longirostrine Allodelphinidae in having the dorsal opening of the mesorostral groove anterior to the rostrum base narrower than the premaxilla, the lateral rostral suture between premaxilla and maxilla not deeply grooved, proportionally wider premaxillae at rostrum base (>60% of the width of the rostrum), and vertex not strongly transversely pinched. *Ensidelphis* belongs to the Platanidelphidi clade in having: Asymmetry of the premaxillae on the rostrum at some distance anterior to the premaxillary foramina, with the right premaxilla distinctly narrower than the left in dorsal view; posterior dorsal infraorbital foramen located along the vertex, more medial than the lateralmost margin of the premaxilla in the cranium; vertex distinctly shifted to the left compared to the sagittal plane of the skull; thick zygomatic process of the squamosal (ratio between the maximum distance from the anteroventral margin of the zygomatic process to the posterodorsal margin, in lateral view, and the vertical distance from the lower margin of the occipital condyles to the vertex of the skull > 0.35); and ventral edge of zygomatic process of squamosal in lateral view almost straight. Within the Platanidelphidi, *Ensidelphis* differs from *Platanista*, *Pomatodelphis,* and *Zarhachis* in the lateral rostral suture between premaxilla and maxilla being not deeply grooved; from *Huaridelphis*, *Notocetus*, and *Squalodelphis* in the presence of a deep lateral groove on the mandible; from *Phocageneus*, *Notocetus,* and *Squalodelphis* in the ventral groove of the tympanic not affecting the whole length of the bone, including the anterior spine; from *Araeodelphis*, *Dilophodelphis*, *Furcacetus*, *Huaridelphis*, *Platanista,* and *Squalodelphis* in the very elongated rostrum (>70% of the CBL); from *Furcacetus, Huaridelphis*, *Macrosqualodelphis*, and *Notocetus* in the smaller size of teeth at rostrum mid-length (diameter <2% of BZW); from *Platanista*, *Pomatodelphis,* and *Zarhachis* in the less elongated mandibular symphysis (61% of the total mandibular length contra >65%) and a consequent smaller angle between the mandibular rami (25° contra roughly 60°).

**Etymology**. *riveroi*, honoring Mariano Eduardo de Rivero y Ustariz (1798–1857), prominent Peruvian geologist and archaeologist.

Description and Comparison

**Ontogeny.** We consider the holotype of *Ensidelphis riveroi* as an adult animal, having: Nasals and frontals fused together at the vertex, fusion of the premaxillae and maxillae at the anterior end of the rostrum, well individualized upper and lower alveoli, and all epiphyses of preserved post-atlas cervical vertebrae completely fused.

**Total body length estimate**. The TBL of *Ensidelphis* was estimated to 1.95 m, using a BZW value of 196 mm in the equation proposed by Pyenson and Sponberg [110] for the stem platanistoids. However, considering the extreme elongation of its rostrum, we suspect that the TBL of *Ensidelphis* was greater than the one calculated with this equation. Therefore, we tried another way to get a better estimate using the extant *Platanista gangetica* and the Miocene *Zarhachis flagellator* for comparison. We chose *Zarhachis* because it is a relative of *Ensidelphis* having the same hyper-longirostrine cranium (see [111,112]; ratio between BZW and CBL equals 0.23 and about 0.22 for the holotype of *Ensidelphis riveroi* and *Z. flagellator* USNM 10485, respectively), and *Platanista* because it is the closest extant relative of *Ensidelphis*. 

The length of the subcomplete thoracic portion (10 vertebrae) of *Z. flagellator* USNM 10485 is about 64.05 cm (measurement after Kellogg [111]; note that the last thoracic of USNM 10485 lacks the centrum, so we estimated its length as a mean value between the ninth thoracic and the first lumbar). The vertebral column of three measured skeletons of *Platanista gangetica* (LDUCZ Z2282, MHNP A7945, MSNUP M272) is between 4.12 and 4.76 times the length of its thoracic portion. Using the same proportions, the length of the postcranial skeleton of *Z. flagellator* can be estimated between 264 and 305 cm and, adding the skull length (119.5 cm according to Kellogg [111]), we obtain a skeletal length between 383 to 424 cm. Based on these estimations, the skeletal length of *Z. flagellator* is between 3.21 to 3.55 times the length of its skull. Considering that the skull length of *Ensidelphis* is 865 cm and applying the same proportions as for *Zarhachis*, we obtain an estimate of the skeletal length of *Ensidelphis* between 277 (= 865 × 3.21) and 307 cm (865 × 3.55). However, the actual body length is slightly greater than the skeletal length due to soft tissues, including intervertebral disks. Consequently a few more centimeters should be added. Therefore, the body length of *Ensidelphis* could have been around 3 m, a length significantly larger that the estimation obtained with the Pyenson and Sponberg [110] equation.

*Cranium* 

**Figure 2:**
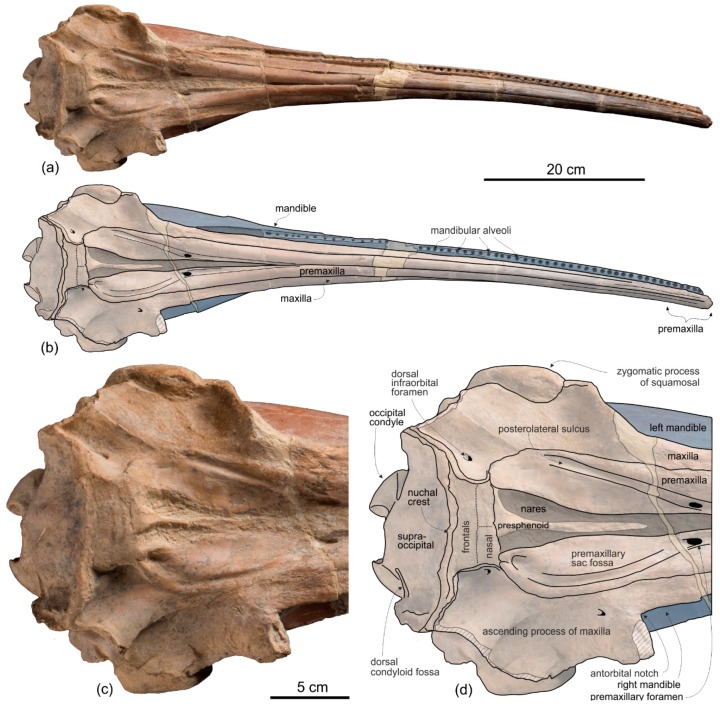
Skull in dorsal view of the holotype (MUSM 3898) of *Ensidelphis riveroi* from the lower Miocene Chilcatay Fm (Zamaca, Pisco Basin, Peru). (**a**,**b**), complete skull; (**c**,**d**), detail of the neurocranium. Linear hatching indicates major breaks, dark shading areas covered by sediment or dental alveoli, and beige shading reconstructed missing parts. In (**b**) and (**d**) the mandibles are shown in blue.

**General morphology**. The most conspicuous character of the cranium of *Ensidelphis* is the extreme elongation of its rostrum (81% of the CBL) (Figure 2a,b; Table 1). Among odontocetes, such an elongated rostrum, a state defined as hyper-longirostry [17], has only been observed in all the allodelphinids (*Allodelphis*, *Goedertius*, *Ninjadelphis*, and *Zarhinocetus*), pomatodelphinine platanistids (*Pomatodelphis* and *Zarhachis*), eurhinodelphinids (i.e., *Eurhinodelphis*, *Schizodelphis*, *Xiphiacetus*, and probably *Ziphiodelphis*), the eoplatanistid *Eoplatanista*, and the lipotid *Parapontoporia*. Compared with the other hyper-longirostral platanistoids, the rostrum of *Ensidelphis* displays a dorsoventral compression intermediate between the less compressed rostra of allodelphinids and the more compressed rostra in pomatodelphinids. As in *Pomatodelphis* and *Zarhachis,* the dorsoventral compression in *Ensidelphis* is more pronounced near the apex of the rostrum. The rostrum of *Ensidelphis* clearly differs from the rostrum of the extant *Platanista* by not displaying a marked transverse compression, a character also observed in a fragmentary early Miocene fossil rostrum referred to Platanistinae [100]. Moreover, the rostrum of the *E. riveroi* holotype is markedly bent towards the right, a possibly natural condition also observed in some adult females of extant *Platanista gangetica* [113] and in some specimens of *Pontoporia blanivillei* [114] (see below for a possible interpretation of this peculiar asymmetry).

As in all platanistoids and in eurhinodelphinids the neurocranium of *Ensidelphis* is anteroposteriorly shortened, its length being about 90% of the postorbital width.

As in all Platanidelphidi and the allodelphinid *Zarhinocetus*, the vertex and the bony nares of *Ensidelphis* are shifted on the left side and the main transverse axis of the nasals is obliquely oriented, even if this feature is less marked than in some other platanistoids (e.g., *Notocetus* and *Huaridelphis*). As a consequence of this shifting, the left frontal is markedly anteroposteriorly shorter than the right on the vertex. 

The vertex is low, flat, and weakly sloping anteroventrally; moreover, in lateral view it does not form a pointed crest as observed in several other platanistoids. The transverse compression of the vertex in *Ensidelphis* is lesser than observed in all Platanidelphidi with the exception of *Huaridelphis*. In fact, in *Ensidelphis* and *Huaridelphis* the minimum transverse width of the vertex is only slightly greater than the transverse width of bony nares, whereas in all other members of the Platanidelphidi the minimum transverse width of the vertex is the same or slightly lower than the width of bony nares. An exception is represented by *Platanista*, displaying a strongly transversely pinched vertex, as observed in the allodelphinids *Allodelphis*, *Goedertius*, *Ninjadelphis,* and *Zarhinocetus*, but not in *Arktocara*, a putative allodelphinid lacking transverse compression of the vertex [115].

The temporal fossa displays a remarkable height (ratio between vertical height of the fossa, in lateral view, and vertical distance from the lower margin of the occipital condyles to the vertex of the skull estimated to 0.70) (Figure 4 and Figure 5). Among platanistoids, a similar height of the temporal fossa (ratio > 0.60) is only observed in *Furcacetus*, *Macrosqualodelphis, Notocetus*, and *Platanista.* Nevertheless, the temporal fossa of *Ensidelphis* differs from the temporal fossa of the aforementioned platanistoids in being anteroposteriorly compressed (height > anteroposterior length).

A clear autapomorphy of *Ensidelphis* is the presence of a peculiar protuberance, here named temporal swelling, on the medial wall of the temporal fossa, just above the squamosal–parietal suture. Clearly visible in lateral (Figure 4c,d, Figure 5c,d) and posterior (Figure 6c,f) views of the cranium, this swelling is interpreted here as an original character, neither due to a pathology, or a trauma, or even post-mortem deformation, since it is present with the same shape and exactly in the same position in both the right and the left fossae. Further supporting the non-artificial nature of this character is the low minimum transverse distance between the temporal crests at the very same level of these swellings (best observed in posterior view of the cranium). The ratio between this distance and BZW is 0.55 for *Ensidelphis*; among other platanistoids, only *Platanista* has a lower value. It is possible that the strong transverse posterior compression of the skull has been compensated (possibly for biomechanical reasons, in relation to the origin of the temporal muscles) by the appearance of the temporal swellings.

**Figure 3:**
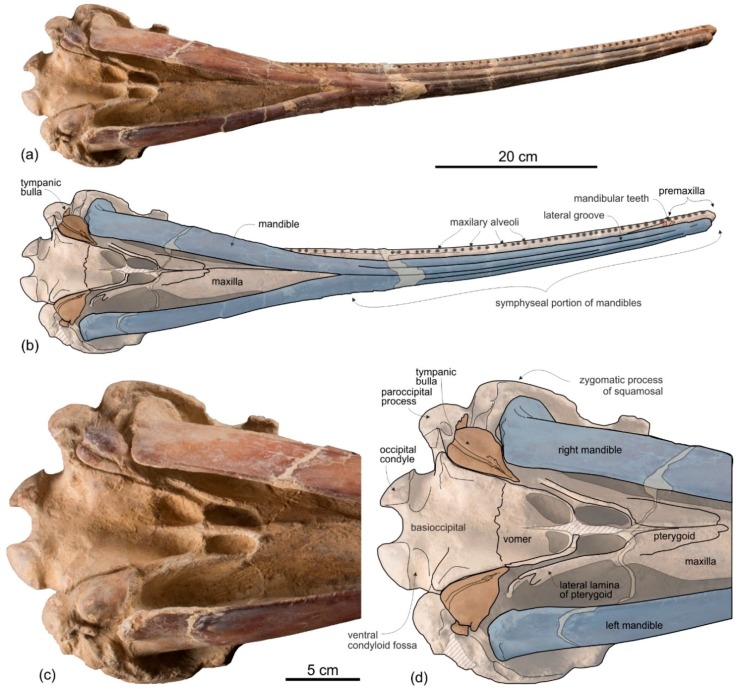
Skull in ventral view of the holotype (MUSM 3898) of *Ensidelphis riveroi* from the lower Miocene Chilcatay Fm (Zamaca, Pisco Basin, Peru). (**a**,**b**), complete skull; (**c**,**d**), detail of the neurocranium. Linear hatching indicates major breaks, dark shading areas covered by sediment or dental alveoli, and beige shading reconstructed missing parts. In (**b**) and (**d**) the mandibles are shown in blue.

**Premaxilla**. The apex of the rostrum is formed by the premaxillae only, as clearly showed in ventral view by the oblique maxillary/premaxillary suture that runs anterolaterally, reaching the lateral margin of the rostrum about 60 mm from the end (Figure 3a,b). Among other platanistoids, the anterior portion of the rostrum being formed by the premaxillae only is also observed in *Dilophodelphis*, *Furcacetus*, *Huaridelphis*, *Notocetus*, and *Squalodelphis*, whereas in all the allodelphinids and platanistids both premaxillae and maxillae are proposed to reach the apex of the rostrum [108,116]. Outside platanistoids, eurhinodelphinids display an even longer anterior premaxillary part of the rostrum (e.g., [117]). 

The premaxillae are joined together dorsomedially, closing the mesorostral groove from the apex of the rostrum to 150 mm anterior to the base of the rostrum (Figure 2a,b). However, the two premaxillae remain distinct from one another by a narrow but clear medial sulcus. Roughly at the level of the right antorbital notch, the opening of the mesorostral groove reaches its maximum transverse width (11 mm). Among platanistoids a wider dorsal opening of the mesorostral groove near the rostrum base is observed in *Medocinia, Squalodelphis,* and in all the allodelphinids.

In dorsal view each premaxilla is laterally fused to the maxilla for about the anterior 100 mm of the rostrum, then the premaxilla–maxilla suture is marked by a thin sulcus becoming deeper towards the posterior portion of the rostrum, but remaining narrow for all the anteroposterior extension of the rostrum, without forming a deep and wide lateral groove as observed in the platanistids *Platanista*, *Pomatodelphis*, and *Zarhachis*.

In the anterior portion of the rostrum the premaxillae are dorsoventrally flattened, then, proceeding posteriorly, their cross section becomes hemicylindrical at rostrum mid-length, before flattening again towards the posterior portion of the rostrum. At the base of the rostrum the dorsal surface of the premaxillae is flat but markedly medioventrally sloping to form a prenarial depression, also observed in most other platanistoids and some other archaic odontocetes (e.g., squalodontids).

About 150 mm anterior to the base of the rostrum, at the level where the premaxillae begin to diverge, the right premaxilla is distinctly transversely narrower than the left premaxilla. This character is observed in all platanistoids, with the exception of *Araeodelphis*. At the base of the rostrum the premaxillae are moderately wide (transverse width of the premaxillae equals 63% of the width of the rostrum), an intermediate condition between allodelphinids (<60%) on the one side, and *Medocinia* and *Squalodelphis* (>75%) on the other side.

A single large premaxillary foramen is present on each premaxilla 35 mm anterior to the rostrum base. The premaxillary foramen is also anterior to the antorbital notch in other platanistoids, with the exception of *Dilophodelphis*, *Macrosqualodelphis*, *Platanista*, and the *Notocetus* skulls from the Chilcatay Fm, all having the premaxillary foramen roughly level with the antorbital notch. The anteromedial and posterolateral sulci are wide and clearly discernible, especially on the right side, whereas the posteromedial sulcus is weakly excavated. The premaxillary sac fossae are moderately transversely concave, they slope medioventrally, and they have the same transverse width. The right ascending process of the premaxilla ends with a posterior point incised by a notch followed anteriorly by a longitudinal wide groove, similar to the premaxillary cleft described in *Waipatia* [99], also observed in most other platanistoids (e.g., [24,26,112]), and in several other archaic odontocetes (e.g., [105,118]). The left ascending process of the premaxilla has a rounded posterior margin without incision or groove. The posterior end of both the right and left ascending processes of the premaxillae contacts the anterolateral angle of the corresponding nasal. Such a limited posterior extension of the premaxillae distinguishes *Ensidelphis* from the other platanistoids, all having the posteromedial margin of the ascending process of both premaxillae in contact with the lateral margin of the nasal. A partial exception is observed in *Furcacetus*, whose right premaxilla only contacts the anterolateral angle of the right nasal. Nevertheless, in *Furcacetus* the left premaxilla displays a significant posterior extension, as in all other platanistoids except *Ensidelphis*. A greater posterior extension of the premaxillae is present in allodelphinids, all having the premaxillae extending posteriorly beyond the nasals.

**Maxilla**. In dorsal view (Figure 2) the maxilla remains transversely narrow along the entire length of the rostrum, showing a flat dorsal surface roughly parallel to the horizontal plane, only weakly ventrolaterally sloping in the mid portion of the rostrum.

Formed by the maxilla, the lateral margin of the posterior portion of the rostrum is dorsoventrally thin and blade-like as in all platanistoids of the Platanidelphidi clade, with the exception of *Huaridelphis, Medocinia*, and *Notocetus*, having a markedly thicker margin.

Both ascending processes of the maxillae are partly broken; consequently, their original dorsal elevation cannot be assessed, although their preserved parts are already higher than the dorsal margin of the rostrum base, a condition shared with all other platanistoids. Due to the incompleteness of the antorbital processes, the shape of the two antorbital notches is unknown.

No dorsal infraorbital foramina are visible around the base of the rostrum, whereas a posterior dorsal infraorbital foramen is located more medial than the lateralmost margin of the premaxilla. The same position of the posterior infraorbital foramina is observed in all known skulls of Platanidelphidi where these foramina are visible.

The posteromedial portions of the ascending processes of the maxillae lateral to the vertex are weakly asymmetrical with the right more anteroposteriorly elongated than the left. Furthermore, the right maxilla descends more abruptly ventrolaterally from the vertex than the left (a condition opposite to that observed in squalodelphinids, all having the left maxilla sloping more abruptly ventrolaterally), to form a deep fossa posterolateral to the right nasal.

The palatal surface of the maxillae is partly covered by the articulated mandibles (Figure 3); however, the latter are partly shifted to the left, making the ventral surface of the right maxilla largely visible along most of the rostrum. This surface is flat and horizontal; near the lateral margin of the rostrum, it is pierced by well-defined alveoli.

**Presphenoid and cribriform plate**. The well-preserved nasal septum runs anteroposteriorly along the sagittal plane from the base of the rostrum to the cribriform plate, separating the bony nares (Figure 2c,d). The cribriform plate borders anteriorly the nasals, reaching dorsally the anterodorsal margin of these bones.

**Nasal**. The vertex is a flat, trapezoidal area where the sutures between bones are not clearly visible (Figure 2c,d). This could be due to some post-mortem abrasion of the skull or, more likely, it could be a genuine anatomical feature. In fact, bone fusion at the vertex is observed in other odontocetes, as for example in the beaked whale *Tusciziphius* [119]. If abrasion can be excluded, the nasals of *Ensidelphis* have a flat dorsal surface. They are probably anteroposteriorly compressed, being shorter than the frontals at the vertex, and, together in dorsal view, form a rectangle with an angle of about 7° between its anterior side and a coronal plane of the skull. A similar oblique orientation of the longitudinal axis of the nasals is present in most Platanidelphidi and also in the archaic odontocete *Waipatia*. Although the nasal-frontal suture is not clearly discernible, being only tentatively reconstructed in Figure 2b,d, it seems that the maximum transverse compression across the vertex occurs at the level of the nasals, with the frontals progressively widening posteriorly. In lateral view the dorsal margin of the nasals slopes anteroventrally, forming an angle of 12° with the dorsal margin of the rostrum. An anterior slope of the nasals is also observed in *Furcacetus* and *Huaridelphis*, being more pronounced in the latter (22–35°), whereas it is absent in other platanistoids such as *Macrosqualodelphis* and *Notocetus,* both displaying an inflated and subhorizontal dorsal surface of the nasals.

**Frontal**. The frontals at the vertex are trapezoidal, flat and slope anteroventrally with the same inclination as the nasals (Figure 2c,d). The suture between the right and left frontals is not discernible.

Both preorbital processes of the frontals are broken and only a posteromedial portion is preserved. In lateral view the best-preserved right process (Figure 5c,d) displays a thickening (ratio between the height of this process measured in lateral view perpendicular to the maxilla-frontal suture and the vertical distance from the lower margin of the occipital condyles to the vertex of the skull = 0.10) that is lesser than in *Dilophodelphis, Furcacetus, Medocinia, Pomatodelphis,* and *Zarhachis*, all having ratios >0.30. However, this value in *Ensidelphis* was measured along the broken lateral surface and a thickening of the preorbital process in its missing lateral portion cannot be excluded. Along the same longitudinal break of the supraorbital process, the section visible in lateral view suggests that the orbit was short and that the postorbital process was robust and triangular.

The articulated mandibles cover most of the ventral surface of the two orbit roofs (Figure 3); consequently, it is not possible to check for the presence of a deep fossa in the medial portion of the orbit roof, as observed in all other Platanidelphidi.

**Figure 4:**
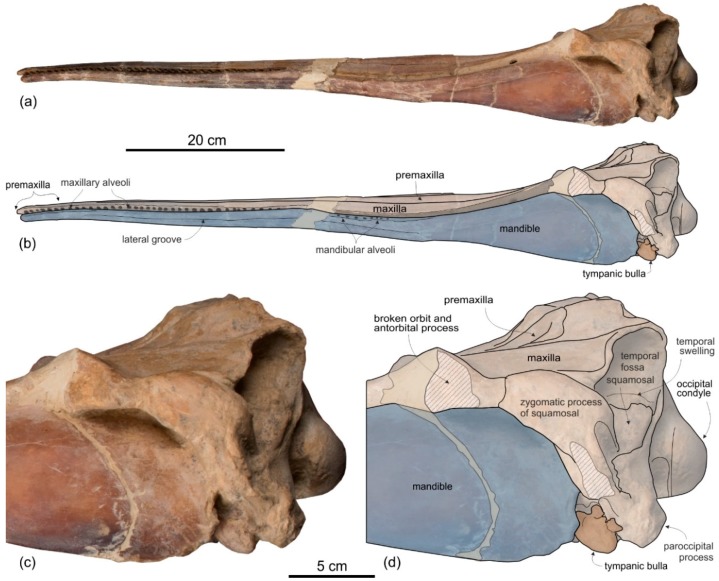
Skull in left lateral view of the holotype (MUSM 3898) of *Ensidelphis riveroi* from the lower Miocene Chilcatay Fm (Zamaca, Pisco Basin, Peru). (**a**,**b**), complete skull; (**c**,**d**), detail of the neurocranium. Linear hatching indicates major breaks, dark shading areas covered by the sediment or dental alveoli, and beige shading reconstructed missing parts. In (**b**) and (**d**) the mandibles are shown in blue and the tympanic bulla in brown.

**Figure 5:**
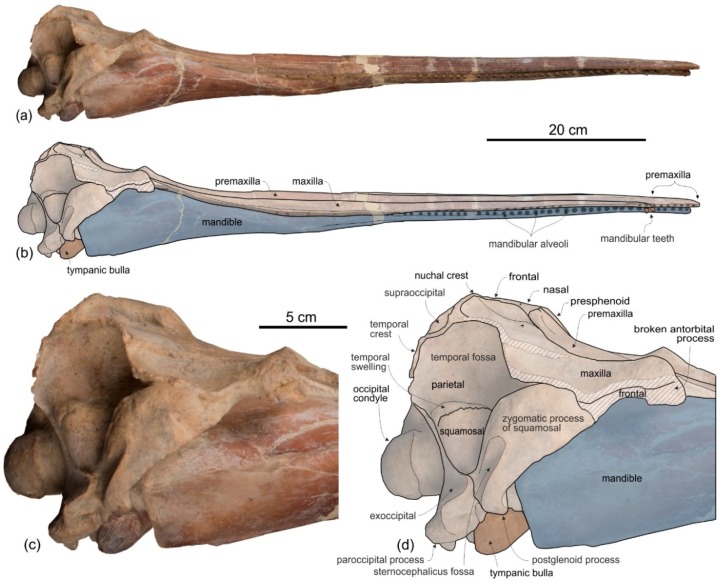
Skull in right lateral view of the holotype (MUSM 3898) of *Ensidelphis riveroi* from the lower Miocene Chilcatay Fm (Zamaca, Pisco Basin, Peru). (**a**,**b**), complete skull; (**c**,**d**), detail of the neurocranium. Linear hatching indicates major breaks, dark shading areas covered by the sediment or dental alveoli, and beige shading reconstructed missing parts. In (**b**) and (**d**) the mandibles are shown in blue, the tympanic bulla in brown, and the teeth in orange.

**Figure 6:**
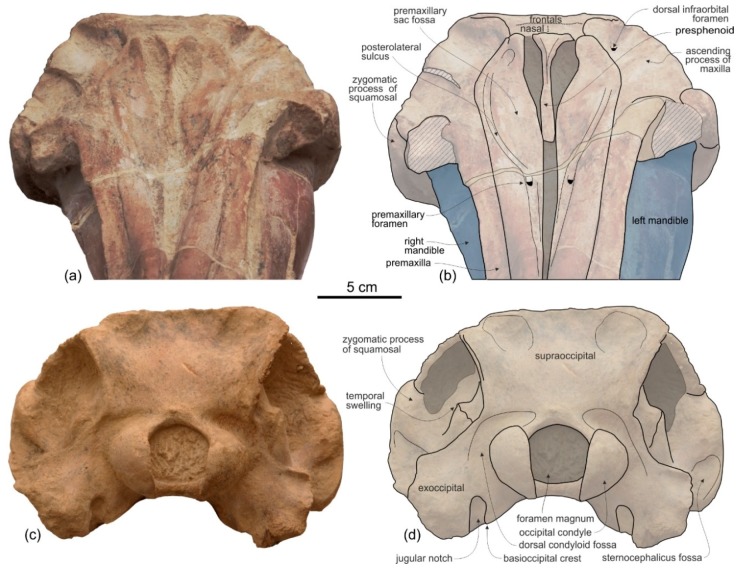
Skull of the holotype (MUSM 3898) of *Ensidelphis riveroi* from the lower Miocene Chilcatay Fm (Zamaca, Pisco Basin, Peru). (**a**,**b**), anterodorsal view; (**c**,**d**), posterior view. Linear hatching indicates major breaks, dark shading areas covered by the sediment, and beige shading reconstructed missing parts. In (**b**) the mandibles are shown in blue.

**Supraoccipital**. In lateral view the nuchal crest is not prominent, even if it represents the highest part of the skull (Figure 4 and Figure 5). In anterior (Figure 6a,b) and posterior (Figure 6c,d) views of the cranium this crest draws a straight horizontal line, whereas in dorsal view it displays a low posterior concavity.

The lateral margin of the supraoccipital forms, together with the parietal, the temporal crest that delimits posterodorsally the temporal fossa. In posterior view the temporal crest is obliquely oriented due to the gradual transverse narrowing of the occipital shield (supraoccipital + exoccipitals) ventrally, as mentioned above. A maximum transverse constriction of the occipital shield is similarly located ventrally, although less marked, in *Macrosqualodelphis.* All the other platanistoids have lateral margins of the occipital shield less laterally concave in posterior view; in several cases these margins are almost straight (i.e., pomatodelphinines and allodelphinids). In all these cases the maximum transverse constriction of the shield is not located ventrally as in *Ensidelphis* and *Macrosqualodelphinus*. A peculiar condition is observed in *Platanista*, displaying a remarkable transverse narrowing of the shield related to the transverse widening of the temporal fossae. However, in *Platanista* the narrowest portion of the occipital is located more dorsally, suggesting a non-homologous origin of this feature in *Ensidelphis* and in the extant South Asian river dolphin.

The posterodorsal surface of the occipital shield is weakly transversely concave and exhibits two wide, roughly circular depressions with a diameter of about 30 mm, one for each side of the supraoccipital. These depressions might indicate the origin of neck muscles, such as m. semispinalis capitis or m. rhomboideus capitis (see [120]). There is no external occipital crest (sensu [27]).

**Palatine**. In ventral view, palatines are not visible in the well exposed posteromedial portion of the rostrum (Figure 3c,d), possibly because they are fully covered by the pterygoids. However, the lateral portion of the neurocranium is still covered by sediment and by the two mandibular rami; consequently, it is not possible to check for the presence of a lateral exposure of the palatine.

**Pterygoid**. The right and left pterygoids, joined together medially, form a narrow point that extends 55 mm beyond the level of the right antorbital notch (Figure 3c,d). Partially covered by the pterygoid plates, the pterygoid sinus fossae also reach beyond the base of the rostrum, as in all platanistoids. The well-preserved left lateral lamina of the pterygoid is a rectilinear plate that contacts posterolaterally the falciform process of the squamosal.

**Jugal-Lacrimal**. The lacrimal and the jugal are lost, on both sides of the skull, due to the breakage of the antorbital processes (Figure 4 and Figure 5).

**Squamosal**. In lateral view (Figure 4 and Figure 5), the zygomatic process of the squamosal is short and robust, with a maximum thickness making 35% of the vertical distance from the lower margin of the occipital condyles to the vertex. A value similar or greater than in *Ensidelphis* was observed in all Platanidelphidi. The zygomatic process of *Ensidelphis* also shares with other Platanidelphidi the globose shape in lateral view, due to the dorsal margin being convex and the ventral margin being not concave (in this case it is straight and obliquely oriented). In particular the dorsal margin of the zygomatic process of *Ensidelphis* draws a regular arch, as in most Platanidelphidi with the exception of *Pomatodelphis* and *Zarhachis*, having the posterior portion of the dorsal margin that slopes more abruptly posteriorly. In *Ensidelphis*, the anterodorsal surface of the zygomatic process is tightly appressed to the postorbital process of the frontal, a feature due to the anterodorsal development of the zygomatic process and shared with all platanistoids and eurhinodelphinids. In lateral view the sternocephalicus fossa extends on the posteroventral portion of the zygomatic process as a narrow and elongate groove that forms an angle of about 65° with the horizontal plane. The postglenoid process is small and anteroventrally direct as in other Platanidelphidi. The squamosal plate is visible in lateral view, forming the ventral portion of the medial wall of the temporal fossa. This wall is locally laterally inflated, forming the peculiar temporal swelling mentioned above, with the maximum lateral expansion (better seen in posterior view) in correspondence to the squamosal-parietal suture. In *Platanista* this suture line is only slightly swollen. 

The ventral surface of the zygomatic processes is almost entirely covered by the articulated mandibular condyles (Figure 3). The thin plate of the left falciform process can be observed, contacting anteriorly the posterolaterally elongated, plate-like lateral lamina of the pterygoid.

**Parietal.** Visible on the medial wall of the temporal fossa the parietal forms most of the lateral wall of the neurocranium (Figure 4 and Figure 5). The aforementioned temporal swelling involves also the ventral portion of the parietal exposed in the temporal fossa.

**Exoccipital**. The occipital condyles are large and posteriorly prominent: they are bordered dorsolaterally by well-excavated dorsal condyloid fossae (visible in posterior view: Figure 6c,d) and ventrally by ventral condyloid fossae (visible in ventral view: Figure 3). The foramen magnum is circular and the jugular notches are deeply incised. The paroccipital process is robust; in lateral view it is thicker and significantly more ventrally extended than the postglenoid process of the squamosal, a condition observed in all Platanidelphidi, with the exception of *Platanista*, which has an atrophied paroccipital process and a large, ventrally extended postglenoid process.

**Basioccipital**. The basioccipital basin is transversely narrow, delimited laterally by robust basioccipital crests that are posterolaterally bent, drawing together a small angle (about 30°) (Figure 3).

**Vomer**. The vomer is visible in ventral view and delimits posteriorly and medially each choana (Figure 3).

**Figure 7:**
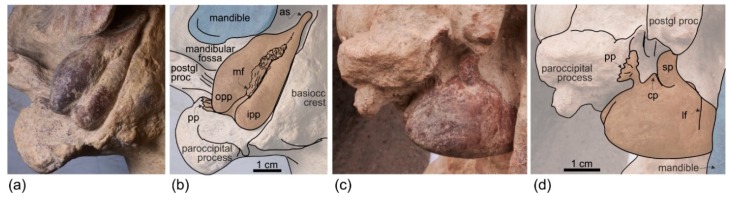
Right tympanic bulla articulated to the skull of the holotype (MUSM 3898) of *Ensidelphis riveroi* from the lower Miocene Chilcatay Fm (Zamaca, Pisco Basin, Peru). (**a**,**b**), ventral view; (**c**,**d**), lateral view. In (**b**) and (**d**) the tympanic bulla is shown in brown and the mandible in blue. Abbreviations: as, anterior spine; basiocc, basioccipital; cp, conical process; ipp, inner posterior prominence; ls, lateral furrow; mf, median furrow; opp, outer posterior prominence; postgl proc, postglenoid process; pp, posterior process; sp, sigmoid process.

**Tympanic bulla**. Both tympanic bullae are preserved in situ, well exposed on the ventral surface of the skull (Figure 3, Figure 7a,b). They are also visible, partially covered by the mandibles, with the skull in lateral view (Figure 4, Figure 5, Figure 7c,d). As in the platanistids *Platanista*, *Pomatodelphis*, and *Zarhachis*, the median furrow is partially filled with spongy bone, and, unlike the squalodelphinids for which the tympanic bulla is preserved (*Notocetus*, *Phocageneus*, and *Squalodelphis*), it does not extend anteriorly on the anterior spine (Figure 7a,b). The outer posterior prominence is slightly shorter than the inner posterior prominence, a condition shared with the allodelphinids *Allodelphis, Ninjadelphis*, and *Zarhinocetus*, whereas in the squalodelphinids *Notocetus*, *Phocageneus*, and *Squalodelphis* the outer and inner posterior prominences have approximately the same posterior extent, and in the platanistids *Platanista*, *Pomatodelphis*, and *Zarhachis* the outer posterior prominence extends farther posteriorly than the inner prominence.

The anterior spine is thin and very long. A more or less elongated anterior spine is present in all platanistoids whose tympanic bulla is preserved, but we observed an extreme elongation as in *Ensidelphis* (anterior spine ca 27% of the total length of the bulla) only in the tympanic bulla associated with the skull MUSM 603 (Figure 7 in [24]). Interestingly enough, the tympanic bulla MUSM 603 shares other features with *Ensidelphis* (e.g., spongy bones in the median furrow, outer posterior prominence posteriorly shorter than the inner posterior prominence), supporting a congeneric referral of the two specimens (see below). The partly exposed lateral surface of the tympanic bulla of *Ensidelphis* (Figure 4, Figure 5, Figure 7c,d) reveals a high and inflated outer lip, as in other platanistoids (e.g., [92]). The conical process is moderately developed and almost in contact posteriorly with the elongated sigmoid process, the latter having its distal portion posterodorsally bent. The lateral furrow is clearly visible. A small portion of the posterior process of the tympanic bulla, articulated with the squamosal and the exoccipital, is also visible.

*Mandible* 

The mandibles are tightly articulated with the cranium, the right and the left mandibular condyles being inside their respective mandibular fossae on the ventral surface of the zygomatic processes of the squamosals (Figure 3). The two mandibles are fused together as in all other Platanistoidea having mandibles preserved and in the Eurhinodelphinoidea (Eurhinodelphinidae + Eoplatanistidae), but not in the longirostral homodont odontocete *Chilcacetus* [57]. In particular, along the 150 mm-long anterior portion of the symphysis the mandibles are ankylosed, with the medial suture being invisible in ventral view. The long mandibular symphysis represents 61% of the total length of the mandibles. Posterior to the symphysis the two mandibular rami draw an angle of 25° in ventral view. The same proportions of the symphysis and a similar angle between the rami are observed in all allodelphinids, whereas the platanistids *Araeodelphis*, *Platanista, Pomatodelphis*, and *Zarhachis* have a more elongated symphysis (>65%) and a consequently larger angle between the mandibular rami (roughly 60°). Among other platanistoids the mandible is only well known in the squalodelphinids *Notocetus* and *Squalodelphis*, both having a shorter symphysis (40% and 43%, respectively) and an angle between the mandibular rami equal to 38°. These values are consistent with a rostrum significantly shorter than in *Ensidelphis*.

The symphysis of *Ensidelphis* is dorsoventrally flattened and it is longitudinally furrowed by two deep lateral grooves, one for each mandible, running from 65 mm from the anterior end of the mandible to the posterior end of the symphysis (Figure 4a,b, Figure 5a,b). Such grooves are present in all platanistoids whose mandible is preserved, with the exception of the squalodelphinids *Huaridelphis*, *Notocetus*, and *Squalodelphis*.

In lateral view the height of the postsymphyseal portion of the mandible increases gradually posteriorly, with both the dorsal and ventral margins forming a low angle with the horizontal axis of the mandible. Among other platanistoids with a mandible preserved, a similar shape of the mandibular rami in lateral view is observed in the allodelphinid *Goedertius* and the squalodelphinid *Notocetus*, whereas a more abrupt posterior elevation of the dorsal margin is present in *Zarhinocetus* and, to an even greater extent, in *Platanista* and *Zarhachis.* The mandible of *Squalodelphis* is too damaged to assess this feature.

*Dentition* 

In ventral view on the right side of the rostrum, 54 alveoli are visible, the six anteriormost being in the premaxilla (Figure 5a,b). However, this value does not represent the total tooth count of the upper right quadrant, considering that the posterior portion of the upper right alveolar row is covered by the mandible. The complete alveolar row is instead exposed on the left mandible (Figure 4a,b); here 62 alveoli are counted and two additional alveoli are estimated to have been originally present in a 28 mm-long reconstructed mid-length portion of the rostrum, resulting in a total tooth count for each mandible of about 64. Since the exposed alveoli on the rostrum have roughly the same longitudinal length and the same spacing as on the mandible, approximately 64 teeth could also be inferred for each upper quadrant. Therefore, the total tooth count of *Ensidelphis* is estimated at 256. A tooth count > 200 characterizes all hyper-longirostrine platanistoids, with a remarkable count of about 315 teeth in *Zarhachis* [111].

The alveoli are small and roughly circular, only slightly longer than transversely wide. Their transverse width varies between 2.4 and 4.4 mm, with an average value of 3.5 mm. The ratio between the transverse width for alveoli at mid rostrum length and the BZW is 0.018. Similarly, low values (<0.02) are observed in all other platanistoids, except in squalodelphinids (ratio > 0.03). The interalveolar septa range between 2.3 and 8.0 mm in length, with an average value of 5.5 mm. On each tooth row, the 6–8 anteriormost alveoli (for premaxillary teeth) are slightly smaller and less spaced than more posterior alveoli. However, with the exception of a few cases, interalveolar septa are longer than the adjacent alveoli, suggesting that when the mouth was closed interlocking teeth of the upper and lower tooth rows did not systematically contact each other. It is significant to underline that for example in the extant longirostrine dolphin *Pontoporia blainvillei* interalveolar spaces increase with the age of the animal (C.M. personal observation); therefore, in *Ensidelphis riveroi* this character could also be subject to ontogenetic variation. 

**Figure 8:**
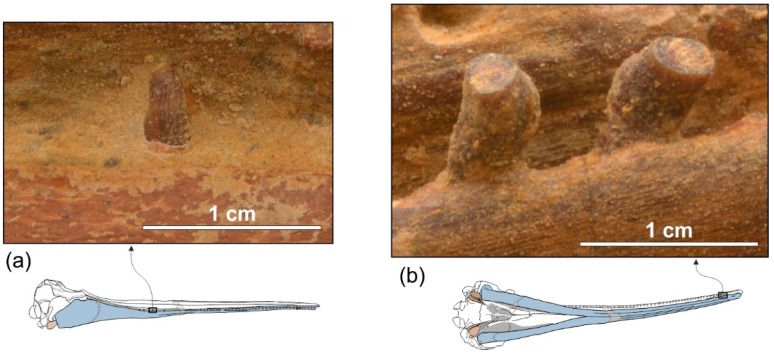
In situ lower teeth of the holotype (MUSM 3898) of *Ensidelphis riveroi* from the lower Miocene Chilcatay Fm (Zamaca, Pisco Basin, Peru). (**a**), posterior tooth; (**b**), two anterior teeth. Tympanic bulla shown in brown and mandibles in blue.

Six teeth are preserved in their alveoli on the right mandible (Figure 5a,b, Figure 8): two in the anterior part of the symphyseal portion (sixth and seventh teeth from the apex) and four in the postsymphyseal portion. All preserved teeth are single rooted, with a simple conical crown lacking accessory denticles or cingula. However, it cannot be excluded that accessory denticles and/or cingula were present in the lost posteriormost teeth, as in some other platanistoids (e.g., *Notocetus* and *Phocageneus*). The crowns of the two preserved anterior teeth are broken and only their basal portion is preserved; their diameter is 4.3 mm. The best-preserved posterior tooth is slender, having a diameter at the base of the crown of 2.8 mm; the height of its almost complete crown is 4.6 mm.

*Cervical Vertebrae* 

**Figure 9:**
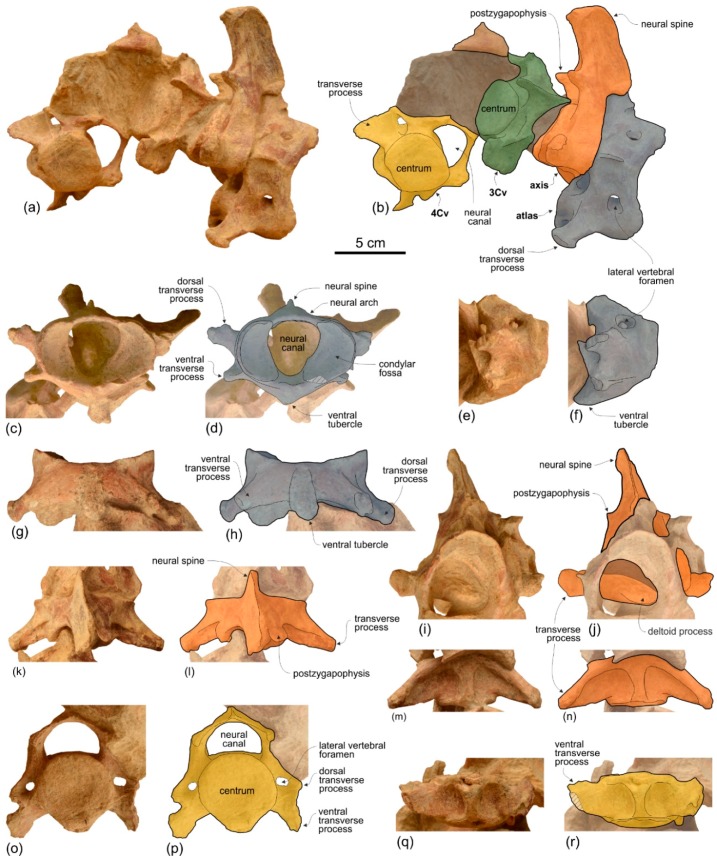
Cervical vertebrae of the holotype (MUSM 3898) of *Ensidelphis riveroi* from the lower Miocene Chilcatay Fm (Zamaca, Pisco Basin, Peru). (**a**,**b**), overall view of the disarticulated cervicals joined by sediment. (**c**–**h**), atlas in anterior (**c**,**d**), lateral (**e**,**f**), and ventral (**g**,**h**) views. (**i**–**n**), axis in anterior (**i**, **j**), dorsal (**k**,**l**), and ventral (**m**, **n**) views. (**o**–**r**), fourth cervical in anterior (**o**,**p**) and ventral (**q**,**r**) views. Linear hatching indicates major breaks. The atlas is shown in blue, the axis in orange, the third cervical in green, the fourth cervical in yellow, and a fragment of unidentified cervical in brown.

None of the atlas, axis, and two other cervicals were fused (Figure 9). They were kept attached by sediment in their position as found in the field, which is not in anatomical connection but strictly associated. We think it is plausible that the original anatomical sequence was maintained and that, therefore, the two vertebrae posterior to the axis represent the third and fourth cervicals.

**Atlas**. The dorsal transverse processes of the atlas are slightly more elongated and robust than the ventral transverse processes (Figure 9c–h). As far as the degree of reduction of the ventral transverse processes is concerned, *Ensidelphis* is intermediate between squalodelphinids (*Huaridelphis*, *Macrosqualodelphis, Notocetus*, and *Phocageneus*), all having an extreme reduction of this process, and the other platanistoids, all having similarly elongated dorsal and ventral transverse processes. As in *Macrosqualodelphis* and *Notocetus* the dorsal transverse processes are widely visible in anterior view, unlike in other platanistoids whose atlas is preserved. This is due to the less posteriorly projected condition of these processes. The neural canal is transversely compressed (ratio between width and height = 0.81), as in *Macrosqualodelphis* (ratio 0.76), whereas it is circular or slightly dorsoventrally compressed in other platanistoids with the atlas preserved. The neural arch is low, pierced by large lateral vertebral foramina for the vertebrarterial canal, and with a transversally thin and short neural spine. The ventral tubercle is robust with a posteroventrally directed pointed tip. Measurements of the cervical vertebrae of MUSM 3898 are provided in Table 2.

**Axis**. Among Platanidelphidi the axis is only known in *Araeodelphis* (USNM 16569), *Huaridelphis* (MUSM 1403, incomplete), *Platanista*, and USNM 206006, an undescribed platanistid from the Calvert Formation (U.S.A.) showing affinities with *Pomatodelphis* and *Zarhachis*. The transverse processes of *Ensidelphis* are similar to those of *Huaridelphis*, being short and robust, triangular in dorsal and ventral view, and posteriorly projected (Figure 9i–n). The transverse processes of *Araeodelphis*, *Platanista*, and USNM 206006 are slender, longer, and posteroventrally projected. The neural arch and the neural spine of *Ensidelphis* are massive and anteroposteriorly thick in lateral view, whereas they are thin in *Araeodelphis* and USNM 206006, and dorsoventrally short and overall triangular in *Platanista*.

**Third and fourth cervicals.** Among Platanidelphidi, the cervical vertebrae posterior to the axis are only known in *Araeodelphis* (USNM 16569), *Huaridelphis* (MUSM 1403, incomplete), *Phocageneus* (USNM 21036, only the third and fifth) *Platanista*, and *Notocetus* (only one in MUSM 1395). Better preserved than the third, the fourth cervical of *Ensidelphis* shows the closest similarities with the third cervical of *Phocageneus*, both having an almost circular centrum in anterior view, a wider than high neural canal, and a low and transversely wide medial keel (Figure 9o–r). However, the ventral transverse processes in C4 of *Ensidelphis* are more ventrally projected and the transverse foramina for the vertebrarterial canal are smaller than in the C3 of *Phocageneus.* Cervicals of the allodelphinids *Allodelphis*, *Goedertius*, and *Ninjadelphis* differ from the cervicals of *Ensidelphis* and of the other Platanidelphidi by having significantly more anteroposteriorly elongated centra [121].

**Platanidelphidi** **indet.**

Figure 10, Table 1

**Referred specimen, locality, and age.** MUSM 3899 is an incomplete cranium with several missing parts including the anterior portion of the rostrum and the right orbital region. Moreover, several areas are damaged, such as the premaxillae on the rostrum, the vertex, and the supraoccipital, and the posterior half of the neurocranium is shifted to the left in respect to the sagittal plane, as clearly seen in ventral view. On the palatal surface of the rostrum 21 and 24 small alveoli for single-rooted teeth are counted on the right and left side, respectively. Earbones and teeth are not preserved. Zamaca locality, Western Ica Valley, Ica Region, Peru (Figure 1a,b). Geographic coordinates: 14°37'28.77" S, 75°38'22.92" W; 345 m above sea level. This specimen was reported in the Zamaca fossil map of Di Celma et al. [22] with the field number ZM 128 and provisionally referred to “Platanistoidea indet.” From the Chilcatay Fm, 38.1 m above the contact with the underlying Otuma Formation, in the Ct1a facies association of the Ct1 allomember [21,22] (Figure 1d). The age of this portion of the Ct1a facies association can be constricted between 19.00 ± 0.25 Ma and 18.08 ± 0.07 Ma (early Burdigalian) on the basis of two volcanic ash layer samples dated by ^40^Ar/^39^Ar [89].

Brief Description and Comparison

**Figure 10:**
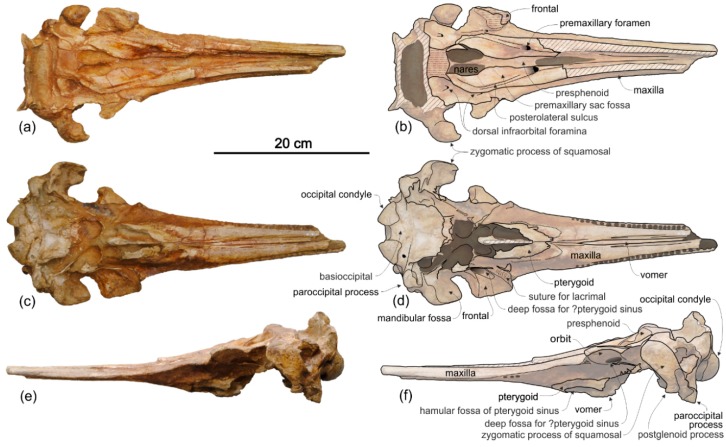
Cranium (MUSM 3899) of Platanidelphidi indet. from the lower Miocene Chilcatay Fm (Zamaca, Pisco Basin, Peru). (**a**,**b**), dorsal view. (**c**,**d**), ventral view. (**e**,**f**), left lateral view. Oblique linear hatching indicates major breaks, horizontal dotted hatching eroded surface, and dark shading areas covered by the sediment or dental alveoli.

The long hamular fossa of the pterygoid sinus extending anteriorly on the palatal surface of the rostrum, the cranium distinctly shorter than wide, and the anterior portion of the zygomatic process of the squamosal tightly appressed to the postorbital process of the frontal are all derived characters allowing us to refer MUSM 3899 to the Platanistoidea superfamily (Figure 10). In particular, MUSM 3899 belongs to the Platanidelphidi clade in having: (1) Asymmetry of the premaxillae on the rostrum at some distance anterior to the premaxillary foramina, with the right premaxilla being distinctly narrower than the left in dorsal view; (2) posterior dorsal infraorbital foramen along the vertex more medial than the lateralmost margin of the premaxilla in the cranium; (3) deep fossa in the frontal on the orbit roof, at the level of the frontal groove, presumably for an orbital lobe of the pterygoid sinus; (4) vertex distinctly shifted to the left compared to the sagittal plane of the skull; (5) palatine not exposed anterior to the pterygoid; (6) dorsoventrally thick zygomatic process of the squamosal; and (7) straight ventral edge of the zygomatic process in lateral view. 

Within the Platanidelphidi MUSM 3899 shares affinities with *Ensidelphis* in its small size (BZW equals 183 cm in MUSM 3899 and 196 cm in the holotype of *Ensidelphis riveroi*; bicondylar width equals 79 cm in MUSM 3899 and 80 cm in the holotype of *E. riveroi*), the narrow and possibly elongated rostrum, the shape of the zygomatic process of the squamosal (with half-circle shaped dorsal margin and straight anteroventral margin), the moderately transversely wide dorsal opening of the mesorostral groove in the rostrum base area, the transversely wide and anteriorly located premaxillary foramen, the limited posterior extension of the ascending processes of the premaxillae, and the small maxillary alveoli (the average of the transverse diameter for the 21 posteriormost alveoli is 3.8 m in both MUSM 3899 and *Ensidelphis*). Moreover MUSM 3899 shares with *Ensidelphis* the absence of any of the derived characters distinguishing both the squalodelphinids and the platanistids within the Platanidelphidi. Nevertheless, MUSM 3899 differs from *Ensidelphis* in the dorsoventrally thinner supraorbital process of the frontal, the transversely narrower vertex (20% and 25% of the BZW in MUSM 3899 and *Ensidelphis*, respectively), the right and left posterior dorsal infraorbital foramina being more laterally located, and the apparent absence of the peculiar temporal swelling on the medial wall of the temporal fossa (but this area is badly preserved on both sides of MUSM 3899's cranium). Moreover, MUSM 3899 differs from *Ensidelphis* in the narrower space between the alveoli (the average length of interalveolar septa between the 21 posteriormost alveoli is 3.0 m in MUSM 3899 and 4.6 in the holotype of *E. riveroi*), although this character, as outlined above, could be subject to intraspecific ontogenetic variation. Based on these observations and considering the fragmentary state of the specimen, we assign MUSM 3899 to an indeterminate basal Platanidelphidi, pending the discovery of more complete specimens.

Squalodelphinidae Dal Piaz, 1917

**Emended diagnosis**. The Squalodelphinidae have the following synapomorphies, absent in other members of the Platanidelphidi clade: (1) Deep, V-shaped, left antorbital notch, related to an anteriorly pointed left antorbital process; (2) left-side torsion of the rostrum with the longitudinal axis of the neurocranium forming an angle of about 5° with the main axis of the rostrum in dorsal view, generating asymmetry of the posterior portion of the rostrum; (3) pars cochlearis of the periotic square-shaped in ventral view; (4) large and thin-edged aperture of the cochlear aqueduct of the periotic; (5) median furrow of the tympanic affecting the whole length of the bone, including the anterior spine; (6) apical extension of the manubrium of the malleus; (7) strong development of the dorsal transverse process of the atlas and extreme reduction of its ventral process. 

**Type genus**. *Squalodelphis* Dal Piaz, 1917

**Other genera included.***Furcacetus* gen. nov., *Huaridelphis*, *Macrosqualodelphis*, *Medocinia*, *Notocetus*, *Phocageneus*.

***Furcacetus***, **gen. nov.**

**LSID**: zoobank.org:act: 7B69F853-98CE-4824-88AC-3294D1B0580D

**Type and only known species**. *Furcacetus flexirostrum*, sp. nov.

**Diagnosis**. As for the type and only referred species.

**Etymology**. From ‘*furca’*, fork in Latin, and ‘*cetus*’, whale in Latin. For the procumbent anterior upper teeth, which, together with the rostrum, look like a fork. Gender masculine.

***Furcacetus flexirostrum***, **sp. nov.**

**Figure 11:**
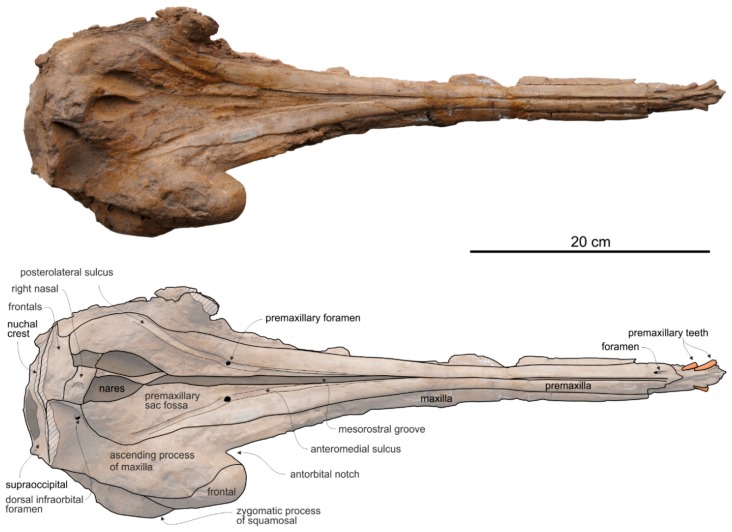
Cranium in dorsal view of the holotype (MUSM 487) of *Furcacetus flexirostrum* from the lower Miocene Chilcatay Fm (Pisco Basin, Peru). Linear hatching indicates major breaks. Teeth are shown in orange.

**Figure 12:**
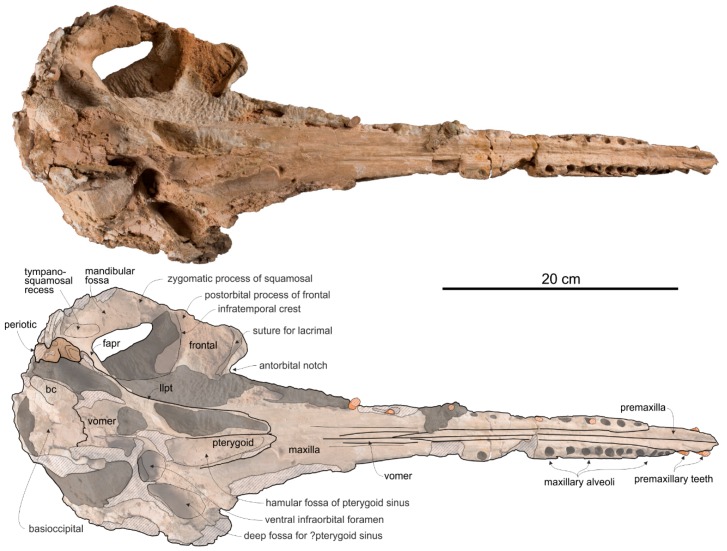
Cranium in ventral view of the holotype (MUSM 487) of *Furcacetus flexirostrum* from the lower Miocene Chilcatay Fm (Pisco Basin, Peru). Linear hatching indicates major breaks and dark shading areas covered by the sediment. Right periotic shown in brown and teeth in orange.

**Figure 13:**
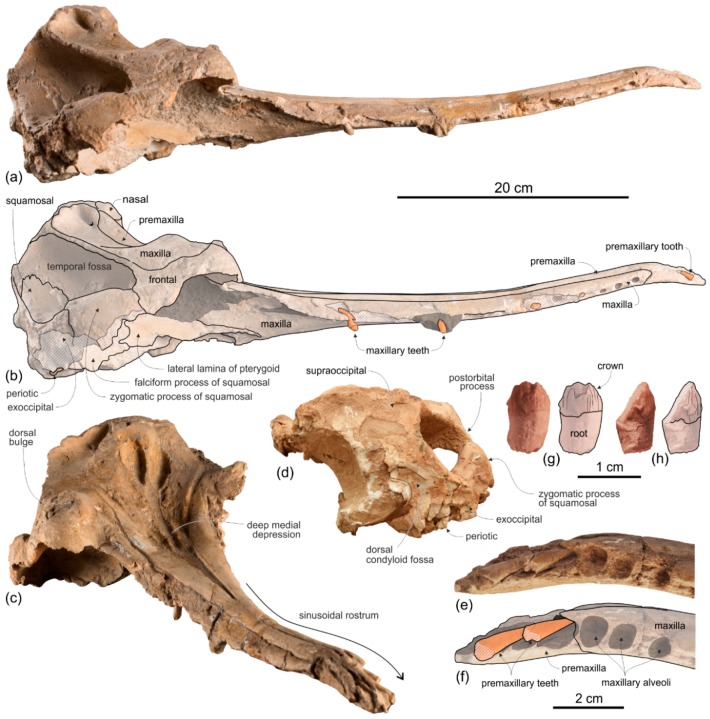
Holotype (MUSM 487) of *Furcacetus flexirostrum* from the lower Miocene Chilcatay Fm (Pisco Basin, Peru). (**a**,**b**), cranium in right lateral view; (**c**) cranium in right anterolateral view; (**d**) cranium in posterior view; (**e**,**f**), detail of the anterior portion of the rostrum in lateral view; (**g**,**h**), posterior tooth in lateral (**g**) and anterior (**h**) views. Linear hatching indicates major breaks and dark shading areas covered by the sediment or the dental alveoli. Right periotic shown in brown and teeth in orange.

Figure 11, Figure 12 and Figure 13, Table 3

**LSID**: zoobank.org:act: B290D90E-D943-4768-956F-28DD1DF75988

**Holotype and only referred specimen**. MUSM 487 consists of a cranium damaged in some parts; in particular the left lateral and the posteroventral portions of the neurocranium are missing. Eight broken teeth are inside their alveoli on the rostrum (five on the maxilla and three on the premaxilla). The incomplete right periotic is still articulated to the cranium.

**Type locality**. MUSM 487 was collected several years ago from layers of the Chilcatay Fm in the Zamaca-Ullujaya area, western Ica Valley, Ica Region, southern Peru (Figure 1a,b). Approximate geographic coordinates: 14°36’ S, 75°38’ W.

**Type horizon**. The exact horizon of the Chilcatay Fm where the holotype of *Furcacetus flexirostrum*, MUSM 487, was discovered is unknown. Nevertheless, the entire stratigraphical sequence of the Chilcatay Fm exposed at Zamaca and Ullujaya localities has been roughly constricted through radiometric dating of ash layers to an interval between 19.25 ± 0.05 and 18.02 ± 0.07 Ma (early Burdigalian) [89].

**Diagnosis**. *Furcacetus* is a small odontocete having an asymmetrical cranium with a narrow and moderately elongated rostrum (about 68% of the CBL), bearing about 25 single-rooted teeth in each upper quadrant. Its rostrum differs from that of all other Platanistoidea s.s., as defined above, in having a sigmoid shape in lateral view and bearing procumbent anterior teeth. *Furcacetus* shares with the other platanistoids the elongated hamular fossa of the pterygoid sinus extending anteriorly on the palatal surface of the rostrum and the neurocranium being shorter than wide (ratio < 0.90). *Furcacetus* belongs to the Platanidelphidi clade in having: asymmetry of the premaxillae on the rostrum at some distance anterior to the premaxillary foramina (ca 18 cm in this case), with the right premaxilla distinctly narrower than the left in dorsal view; posterior dorsal infraorbital foramen along the vertex more medial than the lateralmost margin of the premaxilla on the cranium; deep fossa in the frontal on orbit roof, at the level of the frontal groove; vertex distinctly shifted to the left compared to the sagittal plane of the skull; and thick zygomatic process of the squamosal (ratio between the maximum distance from the anteroventral margin of the zygomatic process to the posterodorsal margin, in lateral view, and the vertical distance from the lower margin of the occipital condyles to the vertex of the skull > 0.35). *Furcacetus* is referred to the Squalodelphinidae in having: pars cochlearis of the periotic square-shaped in ventral view; and longitudinal axis of the neurocranium forming an angle of about 5° with the main axis of the rostrum in dorsal view, generating asymmetry in the posterior portion of the rostrum.

It differs from all other squalodelphinids in having a greater asymmetry of the ascending processes of the premaxillae, the left process being significantly wider and more posteriorly extended than the right; and in having about 25 teeth for upper quadrant, a tooth count that is greater than in *Notocetus* (22–23) and *Squalodelphis* (15), and lower than in *Dilophodelphis* (ca 35) and *Huaridelphis* (28–30) (exact tooth count unknown in *Macrosqualodelphis*, *Medocinia*, and *Phocageneus*). It shares with *Macrosqualodelphis* and *Notocetus* the anteroposteriorly elongated temporal fossa (ratio between horizontal width and vertical height = 1.42) and the correspondingly elongated zygomatic process of the squamosal. It shares with *Dilophodelphis* and *Huaridelphis* a deep, V-shaped right antorbital notch drawing an angle of about 60°. It shares with *Notocetus* the transverse widening of the premaxillae in the anterior portion of the rostrum. It shares with *Dilophodelphis* and *Medocinia* a marked dorsoventral thickening of the preorbital process of the frontal. It differs from *Medocinia,* and *Squalodelphis* in the narrower transverse opening of the mesorostral groove near the rostrum base and in the lesser transverse widening of the premaxilla at rostrum base.

**Etymology**. From ‘*flexus’*, bent in Latin, and ‘*rostrum*’. For the sinusoidal shape of the rostrum in lateral view of the cranium.

Description and Comparison

**Ontogeny.** The closed sutures of the cranial bones, the medial fusion of the frontals on the vertex, and the well-individualized alveoli on the rostrum suggest that the holotype and only referred specimen of *Furcacetus flexirostrum* MUSM 487 was an adult animal.

**Total body length estimate**. Estimating the BZW of the holotype MUSM 487 (left zygomatic process of the squamosal lost) at 240 mm, we used the equation proposed by Pyenson and Sponberg [110] for stem Platanistoidea to obtain an approximate TBL of 234 cm for *Furcacetus flexirostrum*, a value slightly lower than the TBL of *Notocetus vanbenedeni* (237–255 cm based on the BZW of MUSM 3896, and MUSM 3897).

*Cranium* 

**General morphology**. By adding about 20 mm to the length for the missing part of the occipital condyles, the cranium of *Furcacetus* could have reached a CBL of roughly 585 mm, 67% of which being occupied by the rostrum (Table 3). These values are in the range of *Notocetus* (CBL = 580–634 mm; rostrum = 62%–68% of CBL). CBL of other squalodelphinids is either greater (*Macrosqualodelphis*: > 770 mm; *Squalodelphis*: 640 mm) or lesser (*Huaridelphis*: 494 mm; *Dilophodelphis*: 440) than in *Furcacetus*, but in all of them the rostrum is moderately elongated (63–70% of CBL), as in *Furcacetus*.

The rostrum of *Furcacetus* has a peculiar sinusoidal shape in lateral view (Figure 13a–d): from its base, it curves upward until about 50 mm from the apex, where it curves downward until its anterior end. The 50 mm downward-bent anterior portion of the rostrum is formed by the premaxillae only and hosted procumbent incisors (Figure 13e,f). Among other squalodelphinids, we also observed a curved (but not sinusoidal) rostrum in the cranium MUSM 1403 of *Huaridelphis raimondii* (but not in the holotype) and in the crania MUSM 1395 and MUSM 3897 of *Notocetus vanbenedeni* (but not in the holotype and the other referred specimens). Among extant odontocetes, although not associated with procumbent incisors a sinusoidal shape of the rostrum was observed in a few crania of *Pontoporia blainvillei* [114], whereas a rostrum raising anterodorsally, but lacking the anterior downward counter-curvature and, again, the procumbent incisors, was noted in one cranium of the brevirostrine pontoporiid *Brachydelphis mazeasi* [122] and in some crania of *Platanista gangetica* [113,123] and of few delphinid species (e.g., *Delphinus delphis* MZUF 12484, G.B. personal observation; *Delphinus capensis*, Figure 2 in [124] ).

The rostrum of *Furcacetus* is dorsoventrally compressed in its anterior and posterior portions, whereas it becomes more laterally compressed towards mid-length. A deep transverse concavity, involving both the maxillae and the premaxillae, occurs on the dorsal surface of the cranium around the base of the rostrum (Figure 11, Figure 13c). This concavity is laterally overhung by the elevated antorbital regions, which, as in all Platanidelphidi, are distinctly higher than the dorsal margin of the rostrum base in lateral view.

The right antorbital notch is deep and V-shaped, drawing an angle of about 60°, as in *Dilophodelphis* and *Huaridelphis*, whereas other squalodelphinids have a more open right antorbital notch. The left antorbital notch is not preserved and, consequently, it is not possible to check if the right and left antorbital notches were asymmetrical, as in most other platanistoids.

As mentioned above, the original BZW of *Furcacetus* is estimated at 240 mm, suggesting that the neurocranium was proportionally short (79% of BZW), a condition observed in all Platanistoidea.

The asymmetry of the cranium of *Furcacetus* is remarkable, as in most other Platanidelphidi. This asymmetry concerns: (1) The vertex and the bony nares, being shifted to the left side; (2) the right bony naris being transversely broader and more posteriorly located than the left; (3) the left premaxilla being markedly more posteriorly extended than the left; (4) the left frontal at the vertex being anteroposteriorly shorter than the right; and (5) the missing left nasal being probably originally smaller than the right. Moreover, as in all other squalodelphinids the asymmetry of the cranium of *Furcacetus* involves also the posterior portion of the rostrum due to the left lateral shift of the rostrum (main axis of the rostrum in dorsal view forming an angle of about 5° with the longitudinal axis of the neurocranium).

The temporal fossa is significantly elevated (ratio between the vertical height of the fossa, in lateral view, and the vertical distance from the lower margin of the occipital condyles to the vertex of the skull = 0.70), as in *Ensidelphis*, *Macrosqualodelphis*, *Notocetus*, and *Platanista.* The temporal fossa of *Furcacetus* is also anteroposteriorly elongated (ratio between the horizontal length and vertical height = 1.42), similarly to *Macrosqualodelphis* and *Notocetus* [26].

**Premaxilla**. The anterior portion of the rostrum is formed by the premaxilla alone for 50 mm (Figure 11), a condition also observed in all squalodelphinids having the apex of the rostrum preserved (*Dilophodelphis*, *Huaridelphis*, *Notocetus*, and *Squalodelphis*), and in *Ensidelphis*. Partially related to this feature, in dorsal view the lateral premaxilla-maxilla suture is laterally bent and the premaxilla widens transversely towards the apex of the rostrum. A similar widening is observed in *Notocetus*, as well as in squalodontids; in the latter it is similarly associated with procumbent premaxillary teeth (e.g., [98]).

As outlined above, this anterior premaxillary portion of the rostrum is curved downward, dorsoventrally compressed, and bears three alveoli on each side (Figure 13).

The dorsal surface of the anterior portion of the rostrum is poorly preserved, but a foramen that pierces the left premaxilla is clearly visible 55 mm posteriorly to the apex. Foramina piercing the premaxillae on the anterior portion of the rostrum are also observed in *Araeodelphis*, *Dilophodelphis*, and *Notocetus*.

The dorsal opening of the mesorostral groove is very narrow (maximum transverse width = 2 mm) on the 160 mm-long anterior portion of the rostrum; the mesorostral groove is fully closed dorsally, in the middle portion of the rostrum, for a tract 75 mm long, then the premaxillae gradually diverge towards the base of the rostrum, although the opening remains narrow for the whole posterior tract of the mesorostral groove, reaching a maximum transverse width of 8 mm. Among other squalodelphinids, a similarly narrow opening of the mesorostral groove near the rostrum base is present in *Huaridelphis*, *Macrosqualodelphis,* and *Notocetus*, whereas *Medocinia* and *Squalodelphis* display a wider opening.

The premaxilla–maxilla suture is distinct along the whole rostral length, but it is not located in a deep lateral groove as in the platanistids *Platanista*, *Pomatodelphis*, and *Zarhachis*.

The right and the left premaxillae on the rostrum maintain almost the same transverse width for their whole anteroposterior extension, with the right premaxilla only slightly narrower than the left near rostrum mid-length. The asymmetry of the premaxillae, a peculiar feature of the Platanidelphidi, is more pronounced in other squalodelphinids, as for example *Notocetus*.

At the base of the rostrum the premaxilla is transversely wider than the maxilla as in most other squalodelphinids, but not as much as in *Medocinia* and *Squalodelphis*. At this level each premaxilla exhibits a marked medial slope, bounding a deep medial depression that extends posteriorly on the neurocranium, with the premaxillary sac fossae also ventromedially sloping.

The premaxillary foramina (one on each premaxilla) are located roughly at the level of the right antorbital notch, a condition observed, among other platanistoids, in *Dilophodelphis*, *Macrosqualodelphis*, *Platanista*, and the *Notocetus* skulls from the Chilcatay Fm.

The weakly excavated premaxillary sac fossa is laterally delimited by a wide and deep posterolateral sulcus that ends posteriorly where the premaxilla reaches it maximum transverse width. The anteromedial sulcus is shallower than the posterolateral sulcus and, as in *Huaridelphis*, *Macrosqualodelphis,* and *Notocetus*, is significantly elongated, extending about 120 mm anterior to the premaxillary foramen. The posteromedial sulcus is not clearly discernible.

The anterior limit of the bony nares is defined by an angle of the medial margin of each premaxilla, the angle of the right premaxilla being 20 mm posterior to the angle of the left, generating the marked asymmetry of the bony nares mentioned above.

The right and left ascending processes of the premaxilla are strongly asymmetrical. The right process ends with a posteromedial point that contacts the anterolateral angle of the right nasal. The left process extends significantly more posteriorly, far beyond the nares; it was probably medially in contact with the lost left nasal, and reaches posteriorly the frontal. Among other squalodelphinids, an asymmetry of the ascending processes of the premaxillae is also observed in *Dilophodelphis, Huaridelphis,* and *Notocetus*, but less marked than in *Furcacetus,* being limited to a more pointed end of the right ascending process (left process not significantly longer than the right). Unlike in other squalodelphinids and some platanistids (e.g., *Zarhachis*), there is no trace of a longitudinal groove on the posterior portion of the ascending processes of the premaxillae of *Furcacetus* MUSM 487.

In ventral view (Figure 12), on the rostrum the premaxilla–maxilla suture runs obliquely from the posterior margin of the third incisor to a medial point located 138 mm anterior to the right antorbital notch. Consequently, the premaxillae display a narrow and elongated ventral exposure between the palatal processes of the maxillae.

**Maxilla**. The rostral portions of the maxillae are not well preserved, with some missing parts, especially for the left maxilla (Figure 11). However, it is clearly discernible that the transverse width of the dorsal exposure of the maxilla remains narrow for the entire length of the rostrum. Moreover, for most of its anteroposterior extension on the rostrum, the maxilla appears to slope laterally. Approaching the antorbital notch, the maxilla first becomes flat and horizontal, then slopes medially, forming, with the premaxilla, the deep dorsomedian depression at the base of the rostrum.

The only dorsal infraorbital foramen discernible in MUSM 487 is a small posterior foramen piercing the ascending process of the right maxilla very close to the posterior end of the right premaxilla and more medial than the lateralmost margin of the premaxilla. A similar position of the posterior dorsal infraorbital foramen is observed in all the known skulls of Platanidelphidi for which this area is well preserved.

Apparently, the maxilla displays a limited anterolateral extension above the preorbital process of the frontal, although the maxilla–frontal suture it is not clearly discernible on the right side of the cranium and the left side is incompletely preserved. Above the orbit, the right maxilla exhibits a longitudinal bulge posterolateral to the antorbital notch (Figure 13c). A similar bulge is present in other squalodelphinids, but less marked, except in *Dilophodelphis* (thickening significantly greater than in *Furcacetus*) and *Squalodelphis* (thickening similar to *Furcacetus*). It is important to note that the comparison was made using the right side of the neurocranium and that in other squalodelphinids the right preorbital + orbital region is less elevated than the left. It is therefore probable that the thickening on the partly preserved left side of MUSM 487 was even greater than that of the right side. Be that as it may, the thickening observed in *Furcacetus* does not produce an individualized crest forming an acute angle in cross section as observed in the platanistids *Platanista*, *Pomatodelphis*, and *Zarhachis*. As in other squalodelphinids, a marked asymmetry characterizes the posteromedial portion of the ascending processes of the maxillae lateral to the vertex, the right maxilla being significantly anteroposteriorly longer than the left maxilla. Both posteromedial portions of the maxillae slope steeply laterally from the vertex, with the right maxilla being almost vertical.

In ventral view (Figure 12), the palatal surface of the rostrum is mainly formed by the maxillae. It is flat along its anterior two thirds, becoming weakly transversely convex towards the base of the rostrum. Badly preserved and partly covered by sediment, the lateral portions of the palatal surface of the maxillae display relatively large alveoli, some of them being filled by broken teeth, as described in detail below.

**Presphenoid and cribriform plate**. The nasal septum separating the asymmetrical nares is well ossified and elevated (Figure 11). Its posterodorsal margin (medial portion of the cribriform plate) is in contact with the right nasal.

**Nasal**. Only the right nasal is preserved. In dorsal view (Figure 11), it is weakly inflated, rectangular, with a main axis that is obliquely oriented in respect to the frontal plane of the skull. Similar features are observed in *Huaridelphis*, *Macrosqualodelphis*, and *Notocetus.* In lateral view (Figure 13a,b), the nasal of *Furcacetus* appears to slope anteriorly (at least in its anterior portion) as in *Huaridelphis*, but not in *Macrosqualodelphis* and *Notocetus*, having the dorsal surface of the nasal roughly horizontal. The lost left nasal was probably smaller than the right, judging by the size and the shape of the depressed area between the right nasal and the medial margin of the posterior portion of the left premaxilla. If this interpretation is correct, the asymmetry of the nasals in *Furcacetus* is opposite to the asymmetry observed in the other squalodelphinids, all of them having the left nasal slightly larger than the right. The nasal–frontal suture is sinusoidal, with a weak anteromedial convexity, resembling the holotype of *Huaridelphis raimondii* MUSM 1396 and differing from *Macrosqualodelphis* (suture roughly straight), *Notocetus* (suture distinctly anteromedially pointed), and *Medocinia* (suture posteromedially pointed).

**Frontal**. At the vertex, the lateral margin of the right frontal is longitudinally more elongated than the left (Figure 11), a condition shared with *Dilophodelphis*, *Huaridelphis*, *Notocetus*, and *Squalodelphis*. The medial suture between the frontals at the vertex is not discernible. In lateral view (Figure 13a,b), the dorsal surface of the frontals at the vertex appears horizontal, as in *Notocetus*, but not in *Huaridelphis* and *Macrosqualodelphis,* both having frontals anteroventrally sloping.

On the anterolateral portion of the neurocranium, the frontal is widely exposed dorsally, since the maxilla only partially covers the supraorbital and preorbital processes, a condition also observed in *Dilophodelphis*, among other squalodelphinids. In lateral view both these processes appear dorsoventrally thickened. The thickening of the preorbital process is greater than in all other squalodelphinids except *Dilophodelphis, Medocinia,* and the possibly related skull USNM 475596 [24,126]. A more developed preorbital process is also seen in the platanistids *Pomatodelphis* and *Zarhachis*. The postorbital process of the frontal of *Furcacetus* is robust and trapezoidal in lateral view, similar to that of *Notocetus*. The medial portion of the ventral surface of the frontal of *Furcacetus* is excavated in the orbit region by a deep and obliquely oriented fossa (Figure 12). A similar fossa, probably corresponding to an extension of the pterygoid sinus in the orbit region, has been observed in all other Platanidelphidi.

**Supraoccipital**. The supraoccipital is poorly preserved. The eroded nuchal crest is roughly straight in dorsal view (Figure 11). The supraoccipital slopes posteriorly from the vertex with its posterodorsal surface drawing, in lateral view, an angle of ca 50° with the horizontal plane (Figure 13a,b). A similar inclination of the supraoccipital is observed in *Notocetus*, whereas *Huaridelphis* and *Macrosqualodelphis* display a lower inclination and *Squalodelphis* an almost vertical supraoccipital.

**Palatine**. The palatines are not discernible on the ventral surface of the skull (Figure 12). Their anterior portions are probably fully covered by the pterygoids, as generally observed in other Platanidelphidi. Lateral to the pterygoids, the ventral surface of the skull is damaged and partially covered by sediment; it does not show any trace of the lateral exposure of the palatines. 

**Pterygoid**. On the posterior palatal surface of the rostrum (Figure 12), the right and left pterygoids are medially sutured and each is excavated by a pterygoid sinus fossa extending about 30 mm anterior to the right antorbital notch. An elongated hamular fossa of the pterygoid sinus, extending anterior to the rostrum base, is a derived feature shared by all platanistoids. The lateral lamina of the pterygoid runs posterolaterally, reaching the anterior margin of the falciform process of the squamosal. 

**Jugal–Lacrimal**. There is no trace of the jugals and lacrimals. Nevertheless, the ventral surface of the well-preserved preorbital process of the right frontal is marked by a deep oblique groove that represents the suture for the missing lacrimal (Figure 12). This suture indicates that the lacrimal was anteroposteriorly narrow along the lateral wall of the antorbital notch, as in all other platanistoids.

**Squamosal**. In lateral view (Figure 13a,b), the zygomatic process of the squamosal is robust and displays a convex dorsal margin, two features shared with all Platanidelphidi. More specifically, the dorsal margin of the zygomatic process of *Furcacetus* is more similar to that of *Medocinia*, contrasting with those, more regularly arched, of other squalodelphinids. The anterodorsal surface of the zygomatic process of *Furcacetus* tightly contacts the postorbital process of the frontal, a feature related to the anterodorsal extension of the zygomatic process and shared with all platanistoids and eurhinodelphinids. The anteroventral margin of the zygomatic process it not preserved in the holotype of *Furcacetus flexirostrum*. The anteroposterior elongation of the process is significant, similar to that observed in *Macrosqualodelphis* and *Notocetus*, but not in *Dilophodelphis*, *Huaridelphis* and *Ensidelphis*, all having a shorter zygomatic process. In ventral view, posteromedial to the mandibular fossa a longitudinally elongated tympanosquamosal recess is visible. The falciform process projects anteromedially and articulates with the lateral lamina of the pterygoid.

**Parietal.** In lateral view (Figure 13a,b), the parietal is widely exposed in the temporal fossa. There is no trace of the peculiar temporal swelling observed in *Ensidelphis*.

**Exoccipital**. The whole left exoccipital is lost and the right paroccipital process is badly preserved (Figure 13c). A shallow dorsal condyloid fossa is visible dorsolateral to the broken right occipital condyle.

**Basioccipital**. The basioccipital is damaged, but the right basioccipital crest is partially preserved (Figure 12). The angle between right and left basioccipital crests in ventral view is estimated to about 40°.

**Vomer**. The vomer is exposed in ventral view (Figure 12), posterior and medial to each choana, and on the palatal surface of the rostrum, between the maxillae, for a tract extending from roughly 100 to 150 mm anterior to the right antorbital notch.

**Periotic**. The incomplete right periotic is preserved in articulation with the corresponding squamosal (Figure 12). The posterior process is lost and the pars cochlearis is damaged. As in all other squalodelphinids, the anterior process is elongate and not transversely thickened; it does not display the peculiar marked anteromedial bending that characterizes platanistids, and its ventral surface is excavated by an elongate and deep anterior bullar facet. The posterior portion of the pars cochlearis is lost, but its well-preserved anterior wall is rectilinear, suggesting that originally this part of the periotic was square-shaped in dorsal and ventral view, as in all other squalodelphinids.

*Dentition* 

The estimated tooth count for each upper quadrant is 25 (Figure 12). Excluding the longirostrine platanistoids, all having smaller and more numerous teeth, the tooth count of *Furcacetus* is slightly higher than in *Notocetus* (18−23), significantly higher than in *Squalodelphis* (15), and lower than in *Araeodelphis* (ca 50), *Dilophodelphis* (ca 35), and *Huaridelphis* (28−30).

The transverse diameter of the preserved alveoli ranges from 4.5 to 7.2 mm and the spacing between the alveoli increases posteriorly; the premaxillary alveoli are almost in contact and the alveoli at rostrum mid-length are about 15 mm from each other. The posterior alveoli are either not preserved or covered by hard sediment. The ratio between the maximum transverse width for alveoli at rostrum mid-length and the estimated BZW is 0.025, a value similar to *Huaridelphis* (0.026) and smaller than in *Macrosqualodelphis* (0.042) and *Notocetus* (0.040). Judging by the marked oblique orientation of the axis of the three roots embedded in the premaxillae, the incisors (premaxillary teeth) were anterolaterally procumbent (Figure 13e,f), a condition absent in all other Platanistoidea s.s. and more similar to *Waipatia* and squalodontids. The maximum diameter of the root of these incisors is 7 mm. The posteriormost preserved tooth, located about 100 mm anterior to the right antorbital notch, displays a small proximal portion of crown (Figure 13g,h). In particular, only the lateral surface of the crown is well preserved, showing cusp-like cingular nodules and enamel ornamented with longitudinal striations. The maximum diameter of the root and crown of this tooth is 6.0 and 6.5 mm, respectively. About 70 mm anterior to this tooth, along the upper alveolar groove, another tooth has a root with a diameter of 6.0 mm and a crown without accessory denticles (Figure 13a,b).

*Notocetus* Moreno, 1892

**Type and only included species**. *Notocetus vanbenedeni* Moreno, 1892.

***Notocetus vanbenedeni*** **Moreno, 1892**

**Figure 14:**
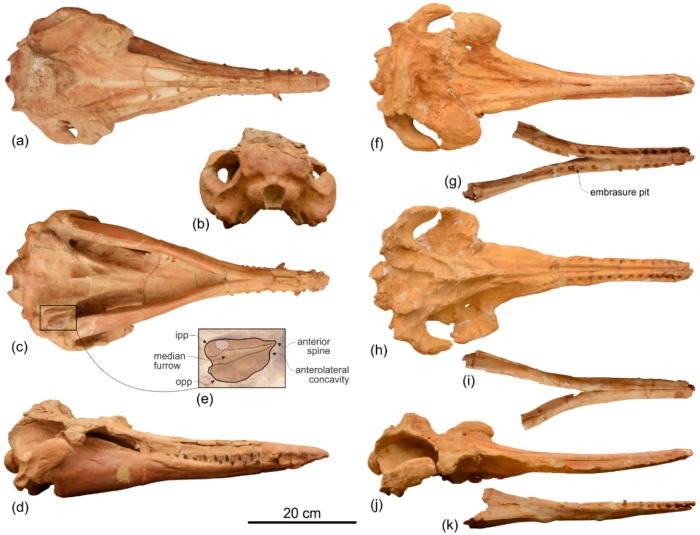
Skulls of *Notocetus vanbenedeni* from the lower Miocene Chilcatay Fm (Zamaca, Pisco Basin, Peru). (**a**–**e**), cranium and articulated mandibles (MUSM 3896) in dorsal (**a**), posterior (**b**), ventral (**c**), and lateral (**d**) views, and detail of the left tympanic bulla in ventral view (**e**); (**f**–**k**), cranium and mandibles (MUSM 3897) in dorsal (**f**, **g**), ventral (**g**,**h**), and left lateral (**j**,**k**) views. In (**e**) the linear hatching indicates a major break.

**Figure 15:**
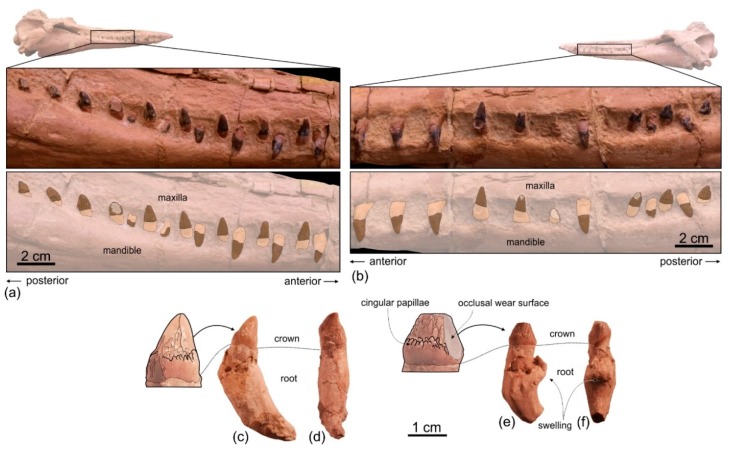
Teeth of *Notocetus vanbenedeni* from the lower Miocene Chilcatay Fm (Zamaca, Pisco Basin, Peru). (**a**,**b**), details of the skull MUSM 3896 in right (**a**) and left (**b**) lateral view showing the lower and upper teeth in place; (**c**–**f**), two detached teeth associated with the skull MUSM 3897 in lateral (**c**,**d**) and posterior (**e**,**f**) views.

**Figure 16:**
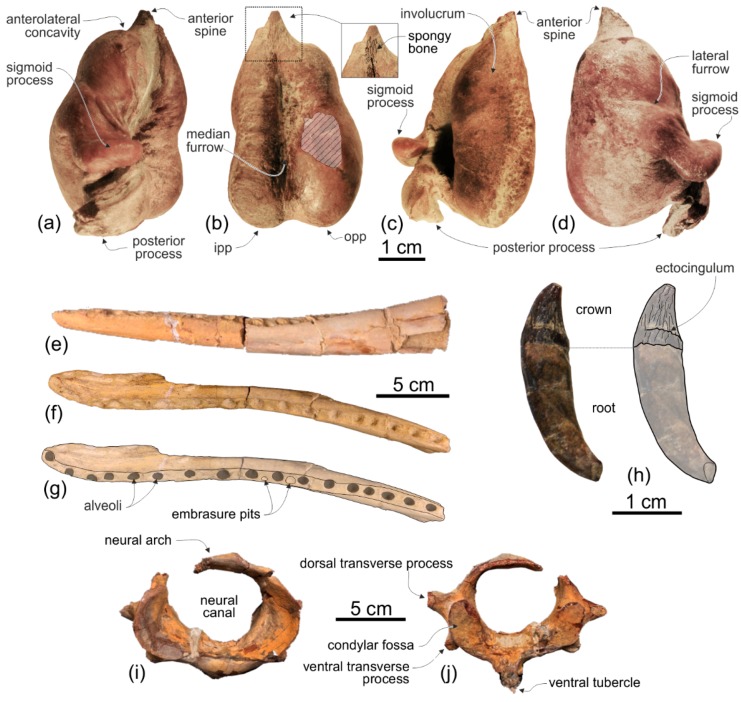
Fragmentary specimen (MUSM 1484) of *Notocetus vanbenedeni* from the lower Miocene Chilcatay Fm (Ullujaya, Pisco Basin, Peru). (**a**–**d**), left tympanic bulla in dorsal (**a**), ventral (**b**), medial (**c**), and lateral (**e**) views; (**e**–**g**), fragment of left mandible in lateral (**e**) and dorsal (**f**, **g**) views; (**h**), tooth in medial view; (**i**,**j**), atlas in anterior (**h**) and posterior (**i**) views. Abbreviations: ipp, inner posterior prominence; opp, outer posterior prominence. Linear hatching indicates major breaks.

**Figure 17:**
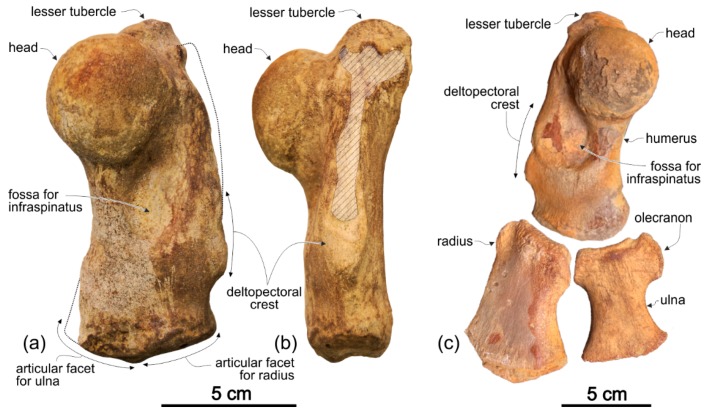
Fragmentary specimen (MUSM 1484) of *Notocetus vanbenedeni* from the lower Miocene Chilcatay Fm (Ullujaya, Pisco Basin, Peru). (**a**,**b**), right humerus in lateral (**a**) and anterior (**b**) views; (**c**), left humerus, radius, and ulna in lateral view. Linear hatching indicates major breaks.

Figure 14, Figure 15, Figure 16 and Figure 17; Table 3, Table 4, Table 5 and Table 6

**Holotype.** MLP 5-5, skull including the mandible but without ear bones; early Miocene of Chubut Province, Argentina [127,128,129].

**Previously referred specimens**. AMNH 9485, skull including the right tympanic bulla, mandibles and some vertebrae and ribs; lower Miocene, Santa Cruz Province, Argentina [129]; AMNH 29026, fragmentary skull including the squamosal, part of the exoccipital, the right periotic, tympanic bulla and malleus, several teeth, a scapula and fragments of vertebrae and ribs; lower Miocene, Chubut Province, Argentina [92]. MUSM 1395, incomplete cranium with associated periotics and without teeth, and one cervical vertebra of the same animal [25]; Ullujaya, Western Ica Valley, Ica Region, Peru, from an unknow horizon of the Chilcatay Fm. The age of the Chilcatay Fm exposed at Ullujaya can be constricted between 19.00 ± 0.25 Ma and 18.02 ± 0.07 Ma (early Burdigalian) on the basis of two volcanic ash layer samples dated by ^40^Ar/^39^Ar [89].

**New referred specimens, localities, and ages.** MUSM 3896 (Figure 14a–e, Figure 15a,b), an almost complete skull including the cranium (only the jugals are missing, the dorsal surface of the vertex is covered by a concretion, and the dorsal surface of the rostrum at mid-length is damaged), fused mandibles in articulation with the cranium, articulated ear bones, and presumably all upper and lower teeth in their respective alveoli. Zamaca locality, western Ica Valley, Ica Region, Peru. Geographic coordinates: 14°37'28.77" S, 75°38'22.92" W; 340 m above sea level. This specimen was reported in the Zamaca fossil map of Di Celma et al. [22] with the field number ZM 47 and provisionally referred to “*Notocetus* sp.”. From the Chilcatay Fm, 7 m above the contact with the underlying Otuma Formation, in the Ct1c facies association of the Ct1 allomember [21,22]. The age of the Ct1c facies association is constricted between 19.25 ± 0.08 Ma and 19.00 ± 0.28 Ma (early Burdigalian) on the basis of two volcanic ash layer samples dated by ^40^Ar/^39^Ar [89]. More details for this age are reported above in the horizon and age description of the holotype of *Ensidelphis riveroi* found ca 3 m above MUSM 3896 in the same locality. 

MUSM 3897 (Figure 14f–k, Figure 15c–f), a cranium with eroded vertex, lacking jugals and ear bones, and without teeth in situ; incomplete mandibles fused, but not articulated to the cranium and with two teeth inside alveoli; and 4 detached teeth, all belonging to the same animal. Zamaca locality, Western Ica Valley, Ica Region, Peru. Geographic coordinates: 14°37'18.05" S, 75°38'34.55" W; 340 m above sea level. This specimen was recently discovered and consequently it was not reported in the previously published Zamaca fossil map [22]. From the Chilcatay Fm, 35 m above the contact with the underlying Otuma Formation, in the upper portion of the Ct1a facies association of the Ct1 allomember [21,22]. The age of this portion of the Ct1a facies association can be constricted between 19.00 ± 0.25 Ma and 18.08 ± 0.07 Ma (early Burdigalian) on the basis of two volcanic ash layer samples dated by ^40^Ar/^39^Ar [89].

MUSM 1484 (Figure 16 and Figure 17) consists of the left tympanic bulla, the incomplete fused mandibles, one tooth, the atlas, the right and left humeri, the left radius and the left ulna of a single individual. Other bones of the same animal, including some vertebrae and ribs are still in the field (Figure 8h–i in [20]); the cranium is not preserved. Ullujaya locality, western Ica Valley, Ica Region, Peru. Geographic coordinates: 14°34'50.8" S, 75°38'44.9" W; 325 m above sea level. This specimen was reported in the Ullujaya fossil map [21] with the field number O4 and provisionally referred to “Squalodelphinidae indet.” From the Chilcatay Fm, 27.9 m above the base of the exposed section at Ullujaya, in the Ct1a facies association of the Ct1 allomember [21,22]. The age of the Chilcatay Fm exposed at Ullujaya can be constricted between 19.00 ± 0.25 Ma and 18.02 ± 0.07 Ma (early Burdigalian) on the basis of two volcanic ash layer samples dated by ^40^Ar/^39^Ar [89].

Brief Description and Comparison of the Newly Referred Specimens

MUSM 3896 and MUSM 3897 fall in the size, shape, and measurements ranges of variation of the skulls previously referred to *Notocetus vanbenedeni* (Table 3). In fact, both skulls show the diagnostic characters of *N. vanbenedeni* as redefined by Bianucci et al. [25] for the description of MUSM 1395. The only significant difference is observed in the tooth count for the upper quadrant of the skull MUSM 3897, being smaller (18) and out of the range of variation (21–23) of the two Argentinian specimens (tooth count unknown in MUSM 1395, 1484, and 3896). Associated with a larger size of the alveoli, this difference could be due to intraspecific variation as observed in the extant *Platanista gangetica*, for which the tooth count of the upper quadrant varies from 26 to 39 [23].

This new Zamaca material confirms that *Notocetus* was a squalodelphinid with a powerful feeding apparatus characterized by robust mandibles, large, interlocking, and moderately heterodont teeth, and anteroposteriorly elongated temporal fossa and zygomatic process of the squamosal. These characters are particularly evident in MUSM 3896 (Figure 14a–e), the only skull of the species that preserves the complete mandibles firmly articulated to the cranium (and probably all the teeth and ear bones in place). For these features *Notocetus* is found to be intermediate between the slightly smaller *Furcacetus* and the significantly larger *Macrosqualodelphis*.

On the ventral surface of the tympanic bulla of MUSM 3896, the wide and deep median furrow extends clearly on the anterior spine (Figure 14e). Such an anterior extension of the median furrow is present in *N. vanbenedeni* AMNH 29026 from Argentina and is considered a synapomorphy of the family Squalodelphinidae. By contrast, the median furrow of the well-preserved tympanic bulla of MUSM 1484 apparently does not extend anteriorly along the anterior spine (Figure 16a–d). However, some longitudinal striations on the ventral surface of the anterior spine could represent spongy bone filling the anterior portion of the median furrow, suggesting that this character could be subject to intraspecific variation, possibly due to ontogenesis. Both tympanic bullae of MUSM 1484 and MUSM 3896 display an elongated anterior spine (although incomplete in MUSM 1484) associated with a marked anterolateral convexity, a derived character observed in all platanistoids. Moreover, the inner and outer posterior prominences of both tympanic bullae have roughly the same posterior extent, a condition shared with the squalodelphinids *Notocetus*, *Phocageneus*, and *Squalodelphis*. In lateral view the tympanic bulla of MUSM 1484 is almost identical to that of *Notocetus vanbenedeni* AMNH 29026, figured by Muizon ([92], Figures. 4a,7a).

The preservation of a complete set of teeth implanted in their alveoli in MUSM 3896 (Figure 15a,b), together with the four detached teeth of MUSM 3897 (Figure 15c–f; Table 4), is relevant since, to date, teeth of *Notocetus* were only known in the fragmentary specimen AMNH 29026 [92]. The Peruvian teeth confirm the observations made by Muizon [92] on AMNH 29026: the two posterior teeth embedded in the left mandible of MUSM 3896 have a low triangular crown with two-three small posterior accessory denticles and cusp-like cingular nodules on the lateral surface; moving to the anterior portion of the mandibles and rostrum of MUSM 3896, tooth crowns become gradually higher, forming a conical point, slightly recurved posteriorly, and lacking the posterior denticles; their enamel is ornamented only by thin longitudinal striations. The four detached teeth of MUSM 3897 have a fusiform root, proportionally large compared to the crown. The two best-preserved teeth have a low triangular crown with weak anterior and posterior carinae and several cusp-like cingular nodules and papillae forming a cingulum near the base of the crown. One of these two teeth displays a wide wear surface on the posterolingual surface of the crown, probably due to the contact with the opposite tooth (attritional wear facet). The same tooth has a peculiar swelling on the posterior surface of the root. The only preserved tooth of MUSM 1484 (Figure 16h) fully overlaps in size and shape with the described anterior teeth of *N. vanbendeni* [92]. This tooth is fusiform, weakly curved, and its crown is conical, weakly mesiodistally compressed, and bearing a posterior carina. The enamel is ornamented by thin anastomosed longitudinal grooves and ridges, more marked on the labial surface of the crown, where an ectocingulum is also visible. 

As in the mandibles of the Argentinian specimens, in MUSM 3896 (Figure 14a,c,d), MUSM 3897 (Figure 14g,i,k), and MUSM 1484 (Figure 16e–g) the mandibular symphysis is fused, the symphyseal portion is markedly dorsoventrally flattened, and, as in *Huaridelphis* and *Squalodelphis*, lacking the pair of lateral grooves observed in members of the families Allodelphinidae and Platanistidae, and in the basal Platanidelphidi *Ensidelphis*. The symphyseal portion of the complete mandibles of MUSM 3896 represents 40% of the total mandibular length, measured parallel to the sagittal plane, a value close to *Squalodelphis* (42%), the only other squalodelphinid for which complete mandibles are known. The angle between the two mandibles equals 38° in both MUSM 3896 and MUSM 3897, similar to *Squalodelphis* but significantly smaller than in all platanistids (> 50°). Embrasure pits are observed between and lateral to the alveoli on the mandibles of MUSM 3897 (Figure 14g) and MUSM 1484 (Figure 16f–g). In lateral view, posterior to the alveolar row, the robust ramus (preserved in MUSM 3896 and MUSM 3897) raises posterodorsally and the coronoid process is significantly elevated. The angular process is prominent, separated by a wide notch from the mandibular condyle.

The almost complete atlas of MUSM 1484 (Figure 16i,j) is close in shape to the atlas of the specimen of *N. vanbenedeni* AMNH 9485, described by True [125], in the extreme reduction of the ventral transverse processes, the neural arch being proportionally lower, and the roughly circular neural canal.

*Description of the Forelimb Bones of MUSM 1484* 

**Figure 18:**
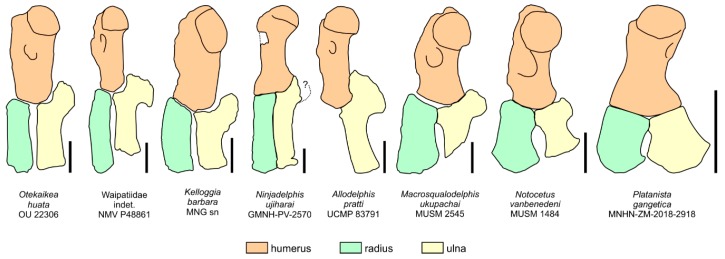
Comparison of the shape of the left humerus, radius, and ulna of *Notocetus vanbenedeni* with the squalodelphinid *Macrosqualodelphis ukupachai*, the extant platanistid *Platanista gangetica*, the allodelphinids *Allodelphis pratti* and *Ninjadelphis ujiharai* (redrawn from [121]), the waipatiid *Otekaikea huata* (redrawn from [104]), an indeterminate waipatiid (redrawn from [131]), and the squalodontid *Kelloggia barbara* (redrawn from [130]) in lateral view. Scale bars equal 5 cm.

The humerus, radius, and ulna, preserved in MUSM 1484 (Figure 17; Table 5), are here described in detail because up to now these bones were unknown in *Notocetus vanbenedeni*. Among other squalodelphinids the forelimb was described only in *Macrosqualodelphis ukupachai*, whereas among other platanistoids these bones are known in the extant *Platanista gangetica* and in the allodelphinids *Allodelphis pratti*, A. *woodburnei*, *Goedertius oregonensis*, and *Zarhinocetus errabundus* ([121], Figure 39). Comparisons (Figure 18; Table 6) were made also with the few other archaic odontocetes having some of these bones preserved (*Awamokoa tokarahi*, *Kelloggia barbara*, *Otekaikea huata*, *Schizodelphis* sp., *Squalodon bellunensis*, *S. calvertensis*, and with derived archaeocetes (e.g., *Cynthiacetus peruvianus*).

**Humerus.** The humerus of MUSM 1484 (Figure 17) is similar in shape but smaller than in *Macrosqualodelphis ukupachai*; among platanistoids it shares only with *M. ukupachai* the large, hemispherical and posterolaterally protruding head, the lesser tubercle being higher than the head, the salient and distally elongated deltopectoral crest, and the large and deep fossa for insertion of M. infraspinatus. In particular, the large, hemispherical and posterolaterally protruding head may be a diagnostic character of squalodelphinids since it was not observed in the humerus of any other cetacean, whereas the lesser tubercle being higher than the head is also observed in *Kelloggia barbara* [130]. Moreover, the humeri of MUSM 1484 and *M. ukupachai* are clearly anteroposteriorly wider than allodelphinid humeri. In fact, the ratio between the anteroposterior width at mid-length and the proximodistal length (B/A in Table 6) is 0.41 and 0.46 in MUSM 1848 and *M. ukupachai*, respectively, whereas in allodelphinids it ranges from 0.26 in *Zarhinocetus errabundus* to 0.31 in *Allodelphis woodburnei*. A humerus as robust as in MUSM 1848 and *M. ukupachai* is instead observed in *Platanista gangetica* (B/A = 0.36–0.41), although the humerus of the only extant platanistid differs from all other platanistoids in lacking the delctopectoral crest and in its pronounced distal anteroposterior widening. 

**Radius.** The radius of MUSM 1484 (Figure 17c) is a mediolaterally flat bone that distally widens anteroposteriorly, more so than in *M. ukupachai* but to a lesser degree than in *P. gangetica*. The proximodistal length of the radius of MUSM 1484 is significantly smaller than the proximodistal length of the humerus, with a ratio (C/A in Table 6) = 0.69, lower than in *M. ukupachai* (0.77), but higher than in *P. gangetica* (0.50–0.56), whereas in the allodelphinid *Ninjadelphis ujiharai* the two bones almost have the same length (C/A = 0.96).The ratio between the anteroposterior width at mid-length and the proximodistal length (D/C) is relatively high (0.51), if compared to *N. ujiharai* (0.24) and some archaic odontocetes, whereas it is similar to *M. ukupachai* (0.49) and lower than in *P. gangetica* (0.68–0.74). 

The radius of MUSM 1484 is articulated with the ulna only for a small proximal portion of its posterior margin; distal to this articulation, the interosseous space between the radius and ulna is very broad, a condition similarly observed in *M. ukupachai* and *P. gangetica*. The articular surface for the first carpals is distally convex and arched in lateral view, as in *P. gangetica.*

**Ulna.** The ulna of MUSM 1484 (Figure 17c), is proximodistally shorter than the radius and, as in *M. ukupachai* and *P. gangetica,* at its mid-length it is anteroposteriorly narrower than the radius. Like the radius, it is mediolaterally flattened and the ratio between its anteroposterior width at mid-length and its proximodistal length (F/E in Table 6) has a value (0.51) higher than in allodelphinids and other archaic odontocetes, but lower than in *P. gangetica* (0.72–0.87) (ratio not computable for the ulna of *M. ukupachai* due to its incompleteness). The olecranon of the ulna of MUSM 1484 is even smaller than in *M. ukupachai*, clearly less developed anteroposteriorly and proximally than in allodelphinids (plesiomorphic condition), whereas the olecranon is completely lacking in *Platanista*.

**cf.** ***Notocetus*** **sp.**

Figure 19

**Referred specimen, locality, and age.** MUSM 1485 consists of a right isolated tympanic bulla. Ullujaya locality, Western Ica Valley, Ica Region, Peru. Exact locality and stratigraphical horizon unknown. Approximate geographic coordinates: 14°35’ S, 75°38’ W. The age of the Chilcatay Fm exposed at Ullujaya can be constricted between 19.00 ± 0.25 Ma and 18.02 ± 0.07 Ma (early Burdigalian) on the basis of two volcanic ash layer samples dated by ^40^Ar/^39^Ar [89].

Brief Description and Comparison

**Figure 19:**
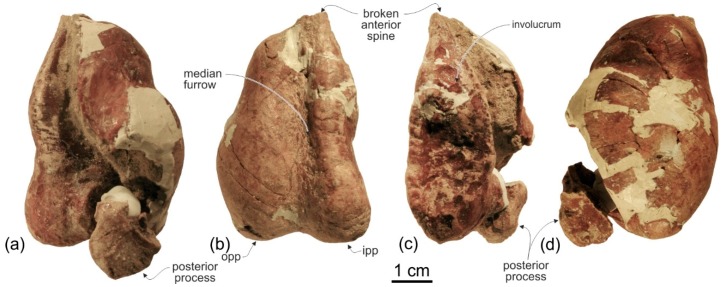
Right tympanic bulla (MUSM 1485) of cf. *Notocetus* sp. from the lower Miocene Chilcatay Fm (Ullujaya, Pisco Basin, Peru) in dorsal (**a**), ventral (**b**), medial (**c**), and lateral (**d**) views. Abbreviations: ipp, inner posterior prominence; opp, outer posterior prominence.

Like MUSM 1484 described above this tympanic bulla is similar in size and shape to those referred to *Notocetus vanbenedeni*. The median furrow (Figure 19b) is only moderately deep, but it extends anteriorly on the preserved portion of the broken anterior spine, a feature that, as outlined above, has been observed in all squalodelphinid tympanic bullae (*Phocageneus*, *Notocetus*, and *Squalodelphis*). Here also, as in the other squalodelphinids, the inner and outer posterior prominences have approximately the same posterior extent. Considering the incompleteness of this specimen, we cautiously refer it to cf. *Notocetus* sp.

Platanistidae Gray, 1846

**Emended diagnosis**. The Platanistidae are characterized by the following synapomorphies, absent in the other members of the Platanidelphidi clade: (1) extremely elongated mandibular symphysis (>65% of the total length of the mandible) and wide angle (>50°) between the mandibular rami; (2) distinct dorsal crest in the antorbital-supraorbital region (character absent in *Araeodelphis*); (3) hook-like articular process on the lateral surface of the periotic (periotic unknown in *Araeodelphis*); (4) outer posterior prominence of the tympanic bulla posteriorly longer than the inner posterior prominence (tympanic bulla unknown in *Araeodelphis*).

**Platanistidae indet. aff.** ***Araeodelphis***

Figure 20; Table 7

**Referred specimen.** MUSM 631, fragmentary skull consisting of rostrum and fused symphyseal portion of mandibles. 

**Locality and horizon.** Western Ica Valley, Ica Region, Zamaca, Peru, Chilcatay Fm. Exact locality and stratigraphical horizon unknown. Approximate geographic coordinates: 14°37’ S, 75°38’ W. The entire stratigraphical sequence of the Chilcatay Fm exposed at Zamaca can be roughly constricted between 19 and 18 Ma (early Burdigalian), considering that a volcanic ash layer located 4 m above the contact between the Chilcatay Fm and the underlying Otuma Formation in the Zamaca area gave an Ar/Ar age of 19.25 ± 0.05 Ma, and a volcanic ash layer from the nearby locality of Ullujaya, located below the contact between the Chilcatay and Pisco formations, gave an age of 18.02 ± 0.07 Ma [89].

Brief Description and Comparison

**Figure 20:**
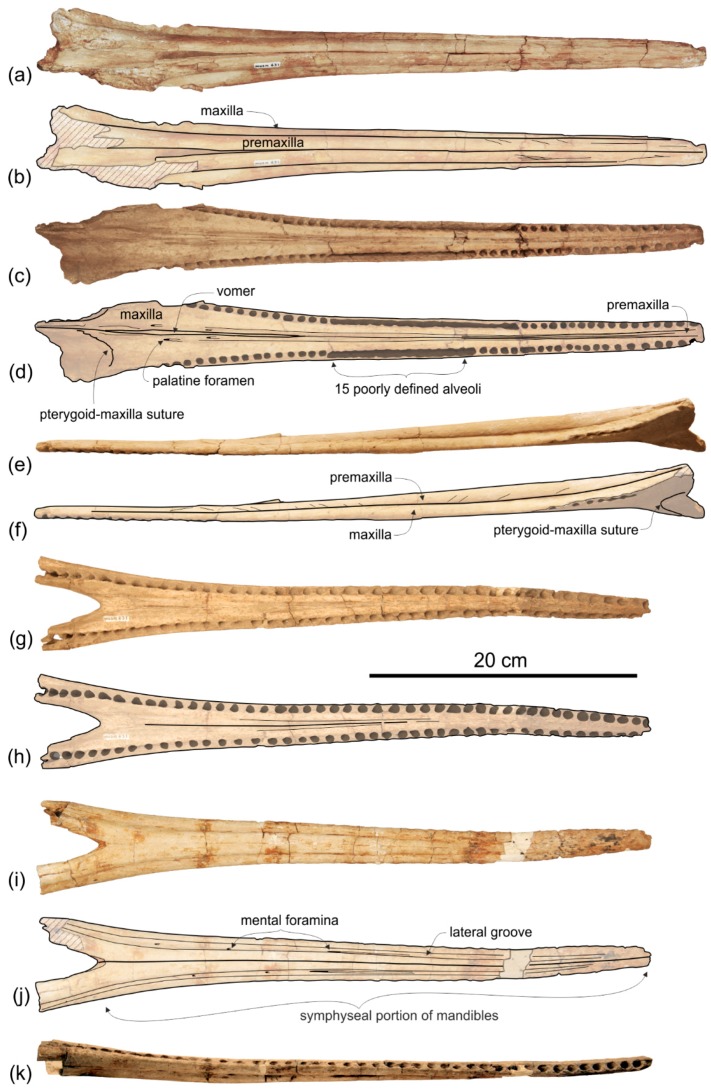
Rostrum and associated mandible (MUSM 631) of Platanistidae indet. aff. *Araeodelphis* from lower Miocene of Chilcatay Fm (Zamaca, Pisco Basin, Peru). (**a**,**b**), rostrum in dorsal view; (**c**,**d**), rostrum in ventral view; (**e**,**f**), rostrum in left lateral view; (**g**,**h**), mandibles in dorsal view; (**i**,**j**), mandibles in ventral view; (**k**), mandibles in right lateral view. Linear hatching indicates major breaks and beige shading reconstructed missing part.

MUSM 631 shows marked affinities with the rostrum and associated mandibles of the early diverging platanistid *Araeodelphis natator* (holotype USNM 10478 and referred cranium USNM 526604 [108,132]), from the late early Miocene (Burdigalian) of Maryland (USA). Shared characters between MUSM 631 and *A. natator* are the following:

(1) Rostrum and mandibles elongated and narrow, with lateral margins parallel in dorsal view (Figure 20a,b), and strongly dorsoventrally compressed in lateral view (Figure 20e,f), especially in their anterior half;
(2) premaxilla fused to the maxilla in the anteriormost portion of the rostrum;
(3) premaxilla-maxilla suture along the rostrum outlined by a distinct sulcus (more excavated in the anterior half of the rostrum), but without the deep lateral groove featuring more derived platanistids;
(4) mesorostral canal dorsally closed or very narrow for the whole rostrum length;
(5) oblique sulci on the dorsal surface of the premaxillae; similar sulci are also present in the longirostral odontocete *Chilcacetus*, but not in other platanistoids;
(6) firmly ankylosed mandibular symphysis being extremely elongated (Figure 20g–j), a distinctive character of the platanistids within the platanistoids; indeed, even if it is not possible to calculate the ratio between the length of the symphysis and the total length of the mandible, the extreme elongation of MUSM 631's symphysis is evidenced by the fact that the posterior end of the symphysis almost reaches the pterygoid-maxilla suture;
(7) ventral surface of the symphyseal portion of each mandible marked by a deep longitudinal groove; this character is also present in all other platanistoids with the exception of squalodelphinids;
(8) symphyseal portion of the mandible bearing a similar number of teeth: 39–40 for MUSM 631 and 38 for USNM 10478;
(9) similar tooth count for each upper quadrant (Figure 20g,h); in fact Godfrey et al. [108] estimated approximately 50 teeth for each quadrant on the rostrum of USNM 526604, a number close to that counted (55) for the almost complete rostrum of MUSM 631; USNM 10478 has 47 alveoli for each upper quadrant, but the rostrum is not as complete as in MUSM 631;
(10) presence of a medial trough between the maxillae on the palatal surface of the rostrum.

Significant differences between MUSM 631 and *Araeodelphis natator* are:

(1) the size, MUSM 631 being larger than USNM 10478 (the length of the mandibular symphyseal portion is 415 mm in MUSM 631 contra 291 mm in USNM 10478; the transverse width of the fused mandibles at the posterior end of the symphysis is 57 mm in MUSM 631 contra 50 mm in USNM 10478) (Table 7); *A. natator* USNM 526604 is even smaller than the holotype (approximately 10%–15% smaller, according to Godfrey et al. [108]);
(2) the mandibular alveoli being proportionally larger and not as many, compared to the upper alveoli in MUSM 631, contra the same size and number of alveoli in lower and upper quadrant in *A. natator* USNM 10478; in fact the transverse diameters of alveoli in MUSM 631 range between 4 and 7 mm in the mandible and 2 and 5.5 mm in the rostrum and there are 40 alveoli in the symphyseal portion of the mandible, for about 53 in the corresponding rostral portion;
(3) the angle formed by the mandibular rami is apparently more acute in MUSM 631 than in *A. natator* USNM 10478; the wide angle (>50°) between the mandibles is an important feature related to the extreme elongation of the symphysis shared by all platanistoids, including *Araeodelphis*; however, the two mandibular rami of MUSM 631 are fragmentary and show some post-mortem fractures that could have changed their original orientation; it is therefore probable that the two mandibles originally formed a wide angle, as expected considering the very long symphysis; and
(4) on the dorsal surface of the rostrum of *A. natator* USNM 526604 the lateral margin of the premaxilla is laterally convex near the antorbital notch, a feature not observed in MUSM 631; however this convexity of the premaxilla could have been originally present in the missing posterior portion of the rostrum of MUSM 631 (the same could be true for *A. natator* USNM 10478, apparently lacking a premaxillary convexity, but with a rostrum incomplete at its base).

Considering the affinities and differences above mentioned, it is possible that MUSM 631 either belongs to: (1) A large specimen of *A. natator,* considering that the size of the mandible and of the alveoli are subject to ontogenetic variation; (2) an undescribed, larger species of *Araeodelphis;* or (3) an unknow genus and species of basal platanistid. Pending the discovery of a more complete specimen, MUSM 631 is here assigned to Platanistidae indet. aff. *Araeodelphis*.

## 5. Phylogeny

**Figure 21:**
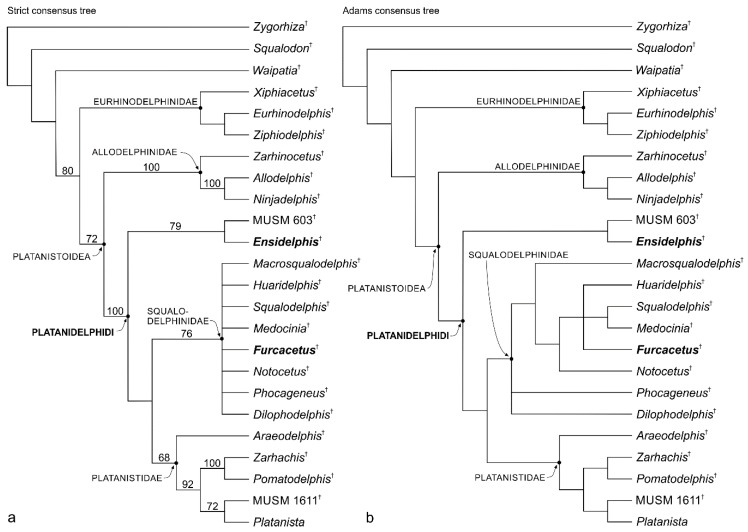
Results of the main phylogenetic analysis showing the relationships of *Ensidelphis* and *Furacetus* with the other Platanistoidea. (**a**), consensus tree of 12 equally parsimonious trees, with tree length = 98, consistency index (CI) = 0.60 and retention index (RI) = 0.82; numbers associated with the nodes are bootstrap values; (**b**), Adams consensus tree.

The cladistic analysis produced 12 equally parsimonious trees, with tree length = 98, consistency index (CI) = 0.60, and retention index (RI) = 0.83. The strict consensus tree, the bootstrap values, and the Adams consensus tree are presented in Figure 21.

Beside the addition of new taxa, the topology here obtained does not differ significantly from those published by Lambert et al. [24], Godfrey et al. [108], Kimura [133], and Bianucci et al. [26]. In fact, in all these analyses the clade that was previously informally named ‘homodont platanistoids’ and that is here redefined as the superfamily Platanistoidea includes three monophyletic families: the Allodelphinidae in the basalmost position, and the sister group-related Platanistidae + Squalodelphinidae. Similarly, our analysis confirms the position of the Eurhinodelphinidae branching between the more basal *Squalodon* + *Waipatia* and the Platanistoidea, supporting the paraphyly of the Platanistoidea as defined by Muizon [92] and Fordyce [99]. The monophyly of the Platanistoidea as redefined here is supported by a bootstrap value of 72 and by the following synapomorphies: (1) Vertex distinctly shifted to the left compared to the sagittal plane of the skull (char. 14, state 1; reversal to state 0 in *Allodelphis* and *Ninjadelphis*); (2) long hamular fossa of the pterygoid sinus extending anteriorly on the palatal surface of the rostrum (char. 17, state 1); (3) presence of an articular rim on the lateral surface of the periotic (char. 20, states 1,2); (4) elongated anterior spine on the tympanic bulla, associated with a marked anterolateral convexity (char. 27, states 1,2); and (5) great reduction of the coracoid process of the scapula and the acromion being located on the anterior edge of the scapula (char. 36, state 1; also present in *Squalodon*). 

Within the Platanistoidea, the clade Platanistidae + Squalodelphinidae forms, together with the new genus *Ensidelphis* and the possibly congeneric MUSM 603, the Platanidelphidi. This new clade is supported by a bootstrap value of 100 and by the following synapomorphies: (1) Asymmetry of the premaxillae on the rostrum at some distance anterior to the premaxillary foramina, with the right premaxilla being distinctly narrower than the left in dorsal view (char. 4, state 1; reversal to state 0 in *Araeodelphis*)*;* (2) posterior dorsal infraorbital foramen(ina) along the vertex more medial than the lateralmost margin of the premaxilla on the cranium (char. 12, state 1); (3) deep fossa in the frontal on orbit roof, at the level of the frontal groove (presumably for orbital lobe of pterygoid sinus) (char. 13, state 1); (4) palatines not contacting each other on the sagittal plane and displaced dorsolaterally (char. 16, states 1,2); (5) thick zygomatic process of the squamosal (ratio between the maximum distance from the anteroventral margin of the zygomatic process to the posterodorsal margin, in lateral view, and the vertical distance from the lower margin of the occipital condyles to the vertex of the skull > 0.35) (char. 18, state 1); (6) dorsal outline of the zygomatic process of the squamosal in lateral view dorsally convex (char. 19, states 1,2; also present in *Squalodon* and in some specimens of *Xiphiacetus*); and (7) ventral edge of the zygomatic process of the squamosal almost straight or convex in lateral view (char. 42, state 1). 

The new phylogeny here presented supports the referral of *Furcacetus* to the Squalodelphinidae, although the relationships within this family remain poorly resolved, as in previous analyses [24,26,108]. However, the Adams consensus tree shows a more satisfactory result, with *Furcacetus* in a derived position among squalodelphinids, forming a clade together with *Huaridelphis* and *Medocinia* + *Squalodelphis*. Interestingly enough, in our new analysis *Dilophodelphis* is placed within the Squalodelphinidae, instead of Platanistidae as proposed by Boersma et al. [101] and Bianucci et al. [26]. Also recovered by Kimura [133], this different familial attribution could be due here to some changes of character states when re-examining the holotype of this genus and to the reformulation of the character 9 dealing with the dorsal crest in the antorbital-supraorbital region. In fact, we consider the prominent dorsal swelling in the antorbital-supraorbital region characterizing the cranium of *Dilophodelphis* homologous to the similar, although less developed thickening observed in other squalodelphinids (e.g., *Squalodelphis*). Such a thickening does not generate a true crest, forming an acute angle in cross section, as observed instead in the platanistids *Platanista*, *Pomatodelphis*, and *Zaharachis*.

## 6. Shape, Function, and Ecology of the Platanistoids of the Chilcatay Fm

**Figure 22:**
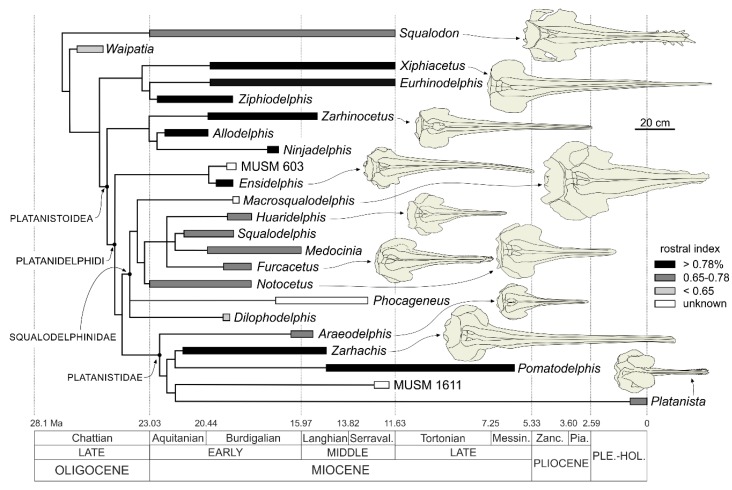
Evolution of size and morphology of the cranium among Platanistoidea using the Adams consensus tree of Figure 21b (the outgroup *Zygorhiza* is not reported). The rostral index is defined as the ratio between the rostrum length and the skull length [17]. Chronostratigraphic scale follows Cohen et al. [139].

**Figure 23:**
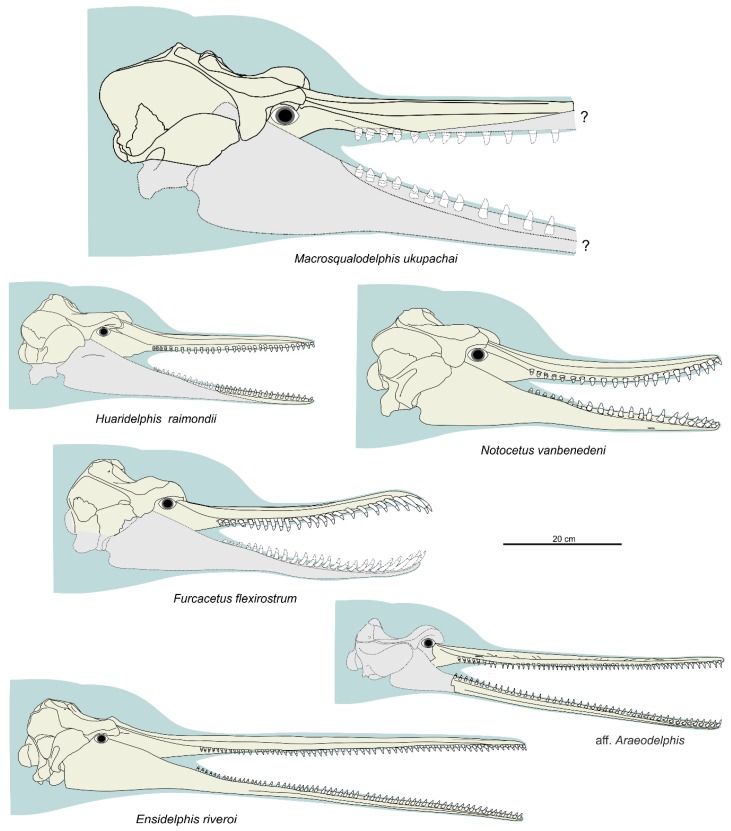
Comparison of the skulls in right lateral views of the platanistoids from lower Miocene of Chilcatay Fm (Zamaca, Pisco Basin, Peru). Stippled lines and grey shading indicate reconstructed missing part for main reconstructed bony parts; green shading for a hypothetical reconstruction of the soft tissue.

The new fossil specimens described here indicate that the diversity and disparity of the platanistoids living along the Peruvian coast during the early Miocene was twice as high as previously pointed out [26]. For a short time interval, well-defined chronostratigraphically between ca 19 and 18 Ma, and in a restricted geographical area, six distinct monogeneric species recovered in the families Squalodelphinidae and Platanistidae and more stemward among basal Platanidelphidi have been described (Figure 22). Concerning morphological disparity, the features showing the most striking variation are the body size, the rostrum length, and the number and size of the teeth (Figure 23). These and other characters related to distinct feeding strategies are reported below for each platanistoid of the Chilcatay Fm, also including the previously described squalodelphinids *Marcrosqualodelphis ukupachai* and *Huaridelphis raimondii* [24,26].

### 6.1. Ensidelphis riveroi

The basal Platanidelphidi *Ensidelphis riveroi* was a medium size odontocete (estimated TBL = ca 3 m) characterized by an extremely elongated rostrum (RI = 0.81) bearing a large number (about 64 for each quadrant) of small teeth (transverse width of alveoli at rostrum mid-length = 1.18 % BZW). It represents a new hyper-longirostrine dolphin, falling in the same category as a series of previously described taxa with a similar cranial morphology (allodelphinids, eoplatanistids, eurhinodelphinids, the lipotid *Parapontoporia*, and pomatodelphinines). Boessenecker et al. [16] and McCurry and Pyenson [17] outlined that hyper-longirorstrine odontocetes are mainly restricted to the early Miocene epoch. The description of *E. riveroi* further supports the radiation of this peculiar morphotype during this short temporal range. The extremely elongated rostrum, being dorsoventrally flattened in its anterior portion and combined with a mandible that is as long as the rostrum are all features shared by *E. riveroi* with the pomatodelphinines *Pomatodelphis inaequalis* and *Zarhachis flagellator*. McCurry and Pyenson [17] suggested that these two pomatodelphinines stunned fish with the mouth closed, performing oscillatory rotation movements mainly along the horizontal plane, like the swordfish *Xiphias gladius* [134], or used the jaws sweeping through the water to grasp the prey as the Indian gharial (*Gavialis gangeticus*). A feeding strategy similar to that of the gharial was also observed in the Amazon river dolphin *Inia geoffrensis* [135]. Nevertheless, if the extreme elongation of the rostrum allows for great angular acceleration and greater speed during sweep feeding [135], the bite force decreases significantly near the anterior end of elongated jaws [136,137], probably preventing the capture of large and medium sized prey. Furthermore, considering the relatively poor maneuverability of a body with such a long rostrum when swimming, we consider a valid alternative hypothesis to the ones mentioned above that *E. riveroi* used its long and slender snout to probe soft sediment for hidden prey, as proposed for the hyper-longirostrine eurhinodelphinids [3,138].

Interestingly enough, *E. riveroi* exhibits a temporal fossa (the area of insertion for the temporalis muscles, involved in jaw adduction) that is significantly smaller than in *Platanista gangetica* and *I. geoffrensis*, suggesting a lower bite force compared to these extant longirostrine dolphins (see Lambert [117] for a similar observation among eurhinodelphinids). On the other side, the peculiar protuberance (temporal swelling) observed on the temporal fossa of *E. riveroi* may provide a stronger attachment surface for part of the temporalis muscles subject to strong stress, due to the very elongated mandible, during the closing of the mouth. Combined with relatively short tooth rows, the significant length of the post-symphyseal portion of the mandible and the marked posterodorsal elevation of its dorsal margin (mirrored by a gradual posterodorsal elevation of the lateral margin of the rostrum) in the skull of *E. riveroi* results in a long, toothless tubular posterior portion of the oral cavity that may have favored the transfer of food to the posterior part of the mouth by suction, before swallowing [140]. Such an elongated post-symphyseal portion of the mandible and associated relatively short tooth row are probably plesiomorphic features, also observed in allodelphinids [121] and partially in squalodelphinids, but not in platanistids, all having the mandibular symphysis and tooth row more posteriorly extended and of which the extant representative *P. gangetica* is considered a typical raptorial feeder [141,142]. In addition to the suction feeding hypothesis, a relatively elongated and robust post-symphyseal portion may have also functioned to strengthen the posterior portion of such narrow and elongated jaws when the mouth was closed. Finally, the long space between the post-symphyseal mandibles could also be related to sound reception, delimiting the “gular pathway” observed for example in the extant *Ziphius cavirostris* [143]. 

In summary, the above observations support the interpretation that *E. riveroi* captured prey using suction-assisted raptorial feeding: (1) either after having stunned it with lateral oscillations of the rostrum; or (2) after having grasped it when laterally sweeping through the water; (3) or after flushing it out of soft sediment along the bottom as proposed by Lambert et al. [54] for the longirostrine ziphiid *Ninoziphius platyrostris*.

Another significant feature of the skull of the holotype of *E. riveroi* is the right-side torsion of the rostrum. Unfortunately, *E. riveroi* is only known from this single skull and consequently it is not possible to check if this peculiar morphology is an anomaly present in a single individual, or rather a distinctive character of the species. In support of the “anomaly” hypothesis, a similar torsion has been observed in some skulls of *P. gangetica* [113], *Inia geoffrensis* [144], and *Pontoporia blainvillei* [114,145]. Even if the causes of this and other cranial anomalies observed on the skull of extant "river dolphins" are still unclear, Gerholdt [145] speculated that a slight twisting due to a trauma in a young individual of *P. blainvillei* could increase disproportionally in the adult due to the positive allometric growth of the rostrum. Having a rostrum significantly longer that *P. blainvillei*, *E. rivireoi*, may have suffered an even greater increase of this anomaly during ontogeny. The hypothesis of the rostral torsion as a distinctive character of the species is instead partially supported by the observation of a similar twisting in all squalodelphinid species and specimens, although in all these cases the rostral torsion processes towards the left side (see below). 

The cervical vertebrae of *E. riveroi* are all free, with their centra anteroposteriorly elongated, suggesting a significant neck elongation similar to that of *P. gangetica*. The distinct ventral tubercle on the ventral surface of the atlas and the deep ventral excavations on the centrum of the axis can be interpreted as surfaces of insertion for a well-developed M. longus colli, responsible for the flexion of the neck [50,146,147]. The massive neural spine of the axis is anteroposteriorly thick in lateral view; it constituted the origin of the M. rectus capitis dorsalis major, the M obliqus capitis caudalis and the M. multifundus cervicalis that extend and rotate the head in extant cetaceans [50,146,147]. In brief, the morphology of the cervical vertebrae suggests that *E. riveroi* had a relatively long neck, possibly with an even greater flexibility than in *P. gangetica*, potentially useful for rotational movements of the snout when searching and/or catching prey. An even more elongated and slender neck was described in allodelphinids [121]. 

### 6.2. aff. Araeodelphis

The fragmentary MUSM 631 specimen here assigned to aff. *Araeodelphis* consists of a rostrum and associated mandibles, both being narrow, elongated, dorsoventrally flattened, and bearing ca 40 small single-rooted teeth for each quadrant. An extant analogue of this platanistoid, which is smaller with a shorter rostrum than *E. riveroi*, is *Pontoporia blainvillei*, having a roughly similar rostrum elongation and a slightly higher tooth count per quadrant (51–58) [148]. *P. blanvillei* feeds mainly upon bottom fish and, secondarily, squid and shrimps [148] and we like to imagine a similar feeding behavior for this small platanistid from Chilcatay Fm.

### 6.3. Furcacetus flexirostrum

The squalodelphinid *Furcacetus flexirostrum* had a body length in the range of the extant *Inia geoffrensis* (estimated TBL = 2.34 m). Its rostrum is moderately elongated (RI = 0.67), anteriorly tapered in dorsal view, and dorsoventrally flattened; the transverse width at rostrum mid-length equals 2.92% of BZW. It bears about 25 teeth in each quadrant. The temporal fossa of *F. flexirostrum* is moderately voluminous. The most peculiar feature of this new species is its curved, sinusoidal rostrum in lateral view, combined with large and proportionally larger, probably procumbent, upper incisors. A similar sinusoidal rostrum, but not associated with large and procumbent anterior teeth, has been observed in some skulls of *Pontoporia blainvillei* [114]. Large, but not procumbent anterior teeth, sometimes combined with an upward but not sinusoidal curvature of the rostrum are also present in *Platanista gangetica* [23,149]. Pilleri [141] reported that captive *P. gangetica* individuals catch their prey by first securing it in the distal third of the jaws, then maneuvering it toward the throat, and swallowing it head first. A similar feeding behavior has been observed for *I. geoffrensis*, taking fish with the anterior teeth to be transferred to the posterior teeth [150]. Procumbent and large anterior teeth are also present in the squalodelphinid *Squalodelphis fabianii* [151] and in several other extinct odontocetes, including the longirostrine squalodontids (e.g., *Squalodon*, *Eosqualodon*, and especially *Kelloggia*) and waipatiids (e.g., [99,130,152]). Squalodontids were moderately heterodont odontocetes that probably used part of their conical and elongate post-apical teeth to grasp and restrain prey and their triangular, laterally compressed denticulated posterior teeth to slice and shear [153]. This probably corresponds to the plesiomorphic feeding strategy among odontocetes, also proposed for the basilosaurids [146]. On the other hand, the anteriormost incisors of squalodontids, as well as of some kentriodontids [154], are almost horizontal and anteriorly directed. Due to this peculiar orientation these teeth are unlikely to be used for grabbing prey items. Fordyce [99] speculated that similar delicate, procumbent, and elongated anteriormost incisors of *Waipatia* were used for display, rather than for predation. However, there is no evidence that the first incisor of *F. flexirostrum* was horizontal as in the squalodontids, *Waipatia* and some kentriodontids. Instead, the combination of a sinusoidal rostrum with procumbent large premaxillary teeth is a feature unique, within the cetaceans, to *F. flexirostrum*. It is reminiscent of the rosette structure observed in the African slender snouted crocodile (*Mecistops cataphractus*), the Indian gharial (*Gavialis gangeticus*), some Muraenosocidae anguilliform fishes, and the spinosaurid *Baryonyx* [155,156]. This rostral morphology has been interpreted as a biomechanical adaptation for biting and grabbing elusive prey items [156].

In summary, considering the moderately wide temporal fossa and the dorsoventrally flat and delicate sigmoidal rostrum bearing procumbent and large incisors, we speculate that *F. flexirostrum* may have fed near the bottom, grasping with quick bites small and elusive prey such as shrimps and small fishes.

### 6.4. Notocetus vanbenedeni

*Notocetus vanbenedeni* is currently the best known squalodelphinid species, thanks to five well-preserved skulls and other fragmentary material from the Chilcatay Fm and the Argentinian Monte León Formation. *N. vanbenedeni* resembles *Furcacetus flexirostrum* for the body size (estimated TBL: 2.37–2.55 m) and the anteriorly tapered and moderately elongated rostrum (RI = 0.62–0.68). However, *N. vanbenedeni* differs from *F. flexirostrum* in the generally more robust skull (including the rostrum), the lower tooth count for each quadrant (18–23), the larger teeth (transverse width at rostrum mid-length = 4% of the BZW), and a more voluminous temporal fossa, being anteroposteriorly more elongated and posterodorsally defined by a developed temporal crest. The interlocking teeth of *N. vanbenedeni* are roughly vertical and their crowns show occlusal wear surfaces and embrasure pits are observed between and laterally to the alveoli. 

In summary, *N. vanbenedeni* probably captured with quick grasps larger and harder prey as compared to *F. flexirostrum,* possibly using its interlocking posterior teeth with low, triangular, and carinated crowns to cut in smaller pieces the prey before swallowing it, in a similar way to that observed in *Inia geoffrensis* [150].

As already mentioned above, all squalodelphinids exhibit a left-side torsion of the rostrum. Being present in the five skulls of *N. vanbenedeni* currently known, this character could be considered a distinctive character of this family rather than an individual ‘anomaly’ as interpreted in some extant longirostrine odontocetes [113,114,144,145]. Although a similar twist of the rostrum is observed in protocetids and basilosaurids [48,157], none of the extant cetaceans consistently shows such a character. It is therefore not easy to associate this shape to a specific function. With a more speculative approach we can propose that the rostral torsion is linked to: (1) either side swimming, a behavior observed in *P. gangetica* in very shallow waters [141,158]; (2) or the leftward shift of the larynx, which would allow homodont odontocetes to swallow entire prey without suffocating [159]; (3) or improved directional hearing, for the reception of high frequency sounds [157,160]. These and other hypotheses should be tested with rigorous morphofunctional analyses in the future, aiming at understanding the evolutionary pressure having generated such a peculiar rostral torsion. Interestingly, this rostral torsion is associated in squalodelphinids to a marked left-side shift of the facial region, more so than in other closely related platanistoids. 

### 6.5. Macrosqualodelphis ukupachai

*Macrosqualodelphis ukupachai* is the largest squalodelphinid and platanistoid of the Chilcatay assemblage (estimated TBL: 3.5 m). It further differs from the other platanistoids from the Chilcatay Fm in the more robust rostrum, larger teeth (transverse width of alveoli at rostrum mid-length = 4.2% of the BZW), more voluminous temporal fossa, and well-developed temporal and nuchal crests [26]. All these features suggest *M. ukupachai* had the role of a macropredator within the odontocete Chilcatay paleocommunity, targeting larger prey.

### 6.6. Huaridelphis raimondii

*Huaridelphis raimondii* is a diminutive squalodelphinid, having an estimated TBL of 2.05 m, a value reached by adult males of *P. gangetica* [23]. Its skull is relatively more gracile than those of the other squalodelphinids, having a slender and more pointed rostrum and a less voluminous temporal fossa [24]. The rostrum is moderately elongated (RI = 0.67). The tooth count for each quadrant (28–30) is greater than in all other squalodelphinids, whereas the teeth are rather small (transverse width of alveoli at rostrum mid-length = 2.6% of the BZW).

It is likely that the feeding strategy of *H. raimondii* was not far from that of *N. vanbenedeni*, but this diminutive squalodelphinid certainly fed on smaller prey.

## 7. Concluding Perspectives on Trophic Partitioning Among the Platanistoids of the Chilcatay Assemblage

The rich sample of platanistoids from the Chilcatay Fm collected in the localities of Ullujaya and Zamaca represents a unique opportunity to reconstruct the ecological roles for a significant portion of a fossil cetacean community that lived in a well-defined and limited space and time. In fact, all platanistoids from the Chilcatay Fm are restricted to an 18–19 Ma time interval and, with the only exception of *Macrosqualodelphis ukupachai*, are precisely positioned along the well-described stratigraphical sequence of a sedimentary basin for which environmental conditions and vertical and horizontal variations are well known [20,21,22,89,90]. Moreover, the platanistoid specimens from Ulluyaja and Zamaca here examined represent a significant part of the entire cetacean assemblage recorded by us (more than 180 specimens of cetaceans, although a significant portion has been referred to Odontoceti indet.).

Expanding on previous, preliminary investigations of this assemblage [24,25,26], the range of sizes and the morphological disparity observed at the level of the oral apparatus for the taxa discussed here suggest that at least part of the trophic partitioning for the platanistoids of the Chilcatay Fm could be related to: (1) different prey sizes (related to the predator's size and the size of its teeth), (2) different prey types (from fish to other marine tetrapods and from cephalopods to harder prey items like benthic crustaceans and shelled mollusks), and (3) different feeding strategies and associated behaviors (raptorial feeding including lateral snapping, stunning of prey, prey probing in soft sediment, suction-assisted raptorial feeding.; see above and [161]. Furthermore, these various foraging behaviors could happen at different depths (from sea surface to seafloor). It should also be expected that these different platanistoid taxa did not all feed at the same distance from the coast (shoreface versus offshore, as the deposits of the Chilcatay Fm record different environments [20,21,22]). However, such a parameter is even more difficult to test, considering that the carcasses of these cetaceans could have drifted for some time with surface currents before sinking to the seafloor for final burial [20,162]. More quantitative methods (e.g., [17,137,163], together with stable isotope analyses on teeth (e.g., [164,165], would be needed to further investigate morphological disparity versus ecological parameters in this species-rich odontocete assemblage from a critical time of cetacean evolutionary history [1,166].

## Figures and Tables

**Table 1:** Measurements on the skulls of *Ensidelphis riveroi* MUSM 3898 (holotype) and Platanidelphidi indet. MUSM 3897 from the Chilcatay Fm (early Miocene, Peru). All measurements are in mm.

Dimension	*Ensidelphis riveroi* MUSM 3898	Platanidelphidi indet. MUSM 3897
Condylobasal length	865	+430
Length of rostrum	697	-
Width of rostrum its rostrum	e111	103
Width of premaxillae at base of rostrum	70	65
Bizygomatic width of skull	196	183
Width of maxillae at midlength of rostrum	33	-
Width of premaxillae at midlength of rostrum	22	-
Maximum width between temporal crests	120	118
Minimum posterior distance between temporal crests	90	98
Length of right orbit	31	-
Height of right temporal fossa	81	-
Length of right temporal fossa	72	-
Length of zygomatic process of squamosal from posglenoid process to tip of zygomatic process	68	-
Maximum width of premaxillae on neurocranium	89	82
Width of right premaxillary sac fossa	37	31
Width of left premaxillary sac fossa	37	31
Width of bony nares	31	34
Minimum posterior distance between maxillae	e42	-
Distance from foramen magnum to nuchal crest	81	-
Width between lateral margins of occipital condyles	80	79
Height of right occipital condyle	41	44
Width of foramen magnum	-	28
Height of foramen magnum	-	22
Maximum width between the exoccipitals	169	149
Length of alveoli at midlength of rostrum	4.0	-
Transverse width of alveoli at midlength of rostrum	3.4	-
Number of teeth per upper tooth row	e64	+21
Total length of mandibles	775	-
Length of symphyseal portion	476	-
Width of mandibles at posterior end of symphysis	46	-
Height of mandibles at posterior end of symphysis	27	-

+, incomplete; - missing data; e, estimate.

**Table 2:** Measurements on the cervical vertebrae of *Ensidelphis riveroi* MUSM 3898 (holotype) from the Chilcatay Fm (early Miocene, Peru). All measurements are in mm.

Dimension	C1	C2	C3	C4
Width of vertebra	121	110	97	e100
Height of vertebra	78	110	71	78
Centrum length	-	33	33	33
Centrum width	-	43	e50	46
Centrum height	-	41	39	40
Neural canal width	36	-	-	33
Neural canal height	41	-	-	20

- missing data; e, estimate.

**Table 3:** Measurements on the crania of *Furcacetus flexirostrum* MUSM 487 (holotype) and *Notocetus vanbenedeni* (MUSM 3896, 3897, 1395) from the Chilcatay Fm (early Miocene, Peru). The crania of *N. vanbedeni* are compared with the holotype (MLP 5-5) and referred specimen (AMNH 9485) from the Monte León Formation (early Miocene, Argentina). All measurements are in mm.

Dimension	*Furcacetus flexirostrum*	*Notocetus vanbenedeni*				
	MUSM 487	MUSM 3896	MUSM 3897	MUSM 1395	AMNH 9485	MLP 5-5 holotype
Condylobasal length	e585	590	+585	600	634*	580
Length of rostrum	392	380	+388	403	433*	360
Width of rostrum at its base	e97	117	126	136	142*	120
Width of premaxillae at base of rostrum	68	67	73	78	89*	81
Orbital width of skull	-	208	238	227	252*	230
Bizygomatic width of skull	e240	243	263	-	-	+220
Width of maxillae at mid-length of rostrum	39	50	49	49	52*	47
Width of premaxillae at mid-length of rostrum	19	27	32	28	30*	25
Maximum width between temporal crests	118	125	162	145	142*	145
Minimum posterior distance between temporal crests	-	117	138	134	128*	140
Length of orbit	-	53	56	55	-	58
Height of temporal fossa	71	83	72	66	74*	80
Length of temporal fossa	101	123	118	108	115*	118
Length of zygomatic process of squamosal from posglenoid process to tip of zygomatic process	-	90	90	91	-	-
Maximum width of premaxillae on neurocranium	101	110	e106	e105	-	108
Width of right premaxillary sac fossa	36	39	40	40	-	58
Width of left premaxillary sac fossa	33	40	40	41	-	48
Maximum distance between premaxillae anterior to bony nares	7	-	-	e15	28*	-
Width of bony nares	40	-	e41	45	-	44
Anterior width of nasals	-	-	-	43	48*	45
Length of medial suture of nasals	-	-	-	14	-	19
Length of medial suture of frontals at vertex	-	-	-	28	-	21
Minimum posterior distance between maxillae	34	-	-	40	-	47
Distance between foramen magnum and nuchal crest	-	95	83	93	97*	91*
Width between lateral margins of occipital condyles	-	85	87	88	-	76
Height of right occipital condyle	-	44	48	48	46*	45
Width of foramen magnum	-	35	41	e39	40*	34
Height of foramen magnum	-	35	34	e29	40*	33*
Length of alveoli at mid-length of rostrum	7	-	10	9	9.5*	7*
Transverse width of alveoli at mid-length of rostrum	7	-	10	9	8*	5.5*
Length of upper tooth row	-	317	+285	+302	363*	315*
Number of teeth per upper tooth row	e25	-	18	+18	21*	23

+, incomplete; - missing data; e, estimate; *, measurement from [125].

**Table 4:** Measurements on detached teeth of *Notocetus vanbenedeni* (MUSM 3897, 1484) from the Chilcatay Fm (early Miocene, Peru). All measurements are in mm.

Dimension	MUSM 3897				MUSM 1484
	a	b	c	d	
Total length	31.5	+28.5	+25.8	25.0	28.6
Root length	27.2	+24.8	+19.9	+19.8	19.7
Crown length	9.4	+7.3	+2.0	5.5	9.2
Maximum transverse diameter of root	8.0	7.6	8.5	7.6	7.1
Maximum mesiodistal diameter of root	10.2	9.4	10.8	10.0	6.2
Transverse diameter at crown base	6.9	6.8	5.9	5.5	7.1
Mesiodistal diameter at crown base	6.5	6.8	5.6	6.8	6.2

+, incomplete.

**Table 5:** Measurements on the forelimb bones of *Notocetus vanbenedeni* (MUSM 1487) compared with the holotype of *Macrosqualodelphis ukupachai* (MUSM 2545), both from the Chilcatay Fm (early Miocene, Peru). All measurements are in mm.

Dimension	*Notocetus vanbenedeni* MUSM 1484	*Macrosqualodelphis ukupachai* MUSM 2545
**Humerus**		
Total proximodistal length	128	160
Anteroposterior width at distal end	51	79
Transverse width at distal end	31	52
Anteroposterior width at mid-length	48	71.
Transverse width at mid-length	26	54
Anteroposterior width of the head	47	64
Transverse width of the head	44	62
**Radius**		
Total proximodistal length	83	129
Transverse width at mid-length	42	60
**Ulna**		
Total proximodistal length	63	-
Transverse width at mid-length	31	-

+, incomplete; - missing data; e, estimate.

**Table 6:** Measurement ratios of the forelimb bones of *Notocetus vanbenedeni* (MUSM 1487) compared with those of other platanistoids, some archaic odontocetes, and the archaeocete *Cynthiacetus peruvianus.*

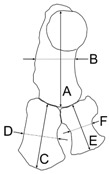	**Species**	**Inv. Number**	**B/A**	**D/C**	**F/E**	**C/A**	**E/A**
	*Notocetus vanbenedeni*	MUSM 1484	0.41	0.51	0.47	0.69	0.51
	*Macrosqualodelphis ukupachai*	MUSM 2545	0.46	0.49	-	0.77	-
	*Platanista gangetica*	MNHN-ZM-2018-2918	0.40	0.68	0.87	0.56	0.55
	*Platanista gangetica*	IRSNB 1507	0.41	0.74	0.74	0.52	0.55
	*Platanista gangetica*	MSNUP M272	0.36	0.90	0.72	0.50	0.47
	*Allodelphis pratti*	UCMP 83791	0.29	-	0.29	-	0.71
	*Allodelphis woodburnei*	SBCM L3210-1	0.31	-	-	-	-
	*Ninjadelphis ujiharai*	GMNH-PV-2570	0.28	0.24	0.25	0.90	0.87
	*Goedertius oregonensis*	LACM 123887	0.30	-	-	-	-
	*Zarhinocetus errabundus*	LACM 21031	0.26	-	-	-	-
	*Xiphiacetus bossi*	USNM 8842	0.45	-	-	-	-
	*Awamokoa tokarahi*	OU 22125	-	-	0.24	-	-
	*Squalodon bellunensis*	MGP-PD 26114	-	0.38	0.34	-	-
	*Squalodon calvertensis*	USNM 10484	-	-	0.34	-	-
	*Kelloggia barbara*	GAS IP S2/S6	0.42	0.40	0.42	0.69	0.60
	*Waipatiidae indet*	NMV P48861	0.37	0.25	-	0.96	-
	*Otekaikea huata*	OU 22306	0.44	0.32	0.37	0.73	0.67
	*Cynthiacetus peruvianus*	MNHN.F.PRU10	0.26	0.19	0.21	0.60	0.57

**Table 7:** Measurements on the rostrum and associated mandibles of aff. *Araeodelphis* MUSM 631 from the Chilcatay Fm (early Miocene, Peru). All measurements are in mm.

Length of the rostrum as preserved	500
Length of left upper tooth row	440
Number of teeth per upper tooth row	55
Length of the mandibles as preserved	455
Length of symphyseal portion of mandibles	415
Width of mandibles at posterior end of symphysis	57
Height of mandible at posterior end of symphysis	22

## Appendix A

### List of Characters for the Phylogenetic Analysis

Characters are polarized with respect to the basilosaurid *Zygorhiza* as the outgroup

1. Rostrum elongation ([167], modified): short, ratio between rostrum length and CBL < 0.75 (0); elongated, ratio > 0.75 (1).
2. Premaxilla at the apex of the rostrum ([168], modified): apex of the rostrum constituted only by the premaxillae on less than 10 per cent of its total length (0); apex of the rostrum constituted only by the premaxillae on more than 10 per cent of its total length and lacking alveoli (1): apex of the rostrum constituted both of the premaxillae and the maxillae (2).
3. Lateral rostral suture between premaxilla and maxilla deeply grooved [99]: no (0); yes (1).
4. Asymmetry of the premaxillae on the rostrum, at some distance anterior to the premaxillary foramina, with the right premaxilla distinctly narrower than the left in dorsal view ([24], modified): absent (0); present (1).
5. Widening of the premaxillae at the rostrum base [24]: narrow premaxillae, ratio between the width of the rostrum and the transverse width of the premaxillae at the antorbital notch < 0.60 (0); wide premaxillae, ratio between 0.60 and 0.75 (1); extremely wide premaxillae nearly reaching the lateral margin of the rostrum, ratio > 0.75 (2).
6. Dorsal opening of the mesorostral groove anterior to the rostrum base ([169], modified): narrower than the premaxilla (0); wider than the premaxilla (1).
7. Deep, V-shaped, left antorbital notch, related to an anteriorly pointed antorbital process [24]: no (0); yes (1).
8. Elevated antorbital region, distinctly higher than the dorsal margin of the rostrum base in lateral view [24]: no (0); yes (1).
9. Distinct prominent dorsal crest in the antorbital-supraorbital region forming an acute angle in cross section ([24], modified): no (0); yes (1).
10. Thickening of the antorbital process of the frontal, quantified as a ratio between the height of this process measured in lateral view perpendicular to the maxilla-frontal suture and the vertical distance from the lower margin of the occipital condyles to the vertex of the skull [24]; absent, ratio < 0.25 (0); present, ratio > 0.30 (1).
11. Widening of the neurocranium [24]: neurocranium roughly as long as wide or longer than wide with ratio between neurocranium length (longitudinal, from occipital condyles to level of antorbital notches) and postorbital width > 0.90 (0); neurocranium distinctly shorter than wide with ratio < 0.90 (1).
12. Posterior infraorbital foramen(ina) along the vertex more medial than the lateralmost margin of the premaxilla in the cranium [24]: no (0); yes (1).
13. Deep fossa in the frontal on orbit roof, at the level of the frontal groove [24]: no (0); yes (1).
14. Vertex distinctly shifted to the left compared to the sagittal plane of the skull [24]: no (0); yes (1).
15. Transverse premaxillary crest on the vertex [168]: absent (0), present (1).
16. Ventral exposure of the palatine ([92], modified): palatine exposed and joined sagittally anterior to the pterygoids (0); palatines lose sagittal contact and are displaced dorsolaterally but visible on the palate (1); palatines displaced dorsolaterally and totally covered by pterygoids (2).
17. Hamular fossa of the pterygoid sinus [54]: short, not reaching anteriorly the level of the antorbital notch (0); long, extending anteriorly on the palatal surface of the rostrum (1).
18. Thickening of the zygomatic process of the squamosal [24]; absent, ratio between the maximum distance from the anteroventral margin of the zygomatic process to the posterodorsal margin, in lateral view, and the vertical distance from the lower margin of the occipital condyles to the vertex of the skull < 0.35 (0); present, ratio > 0.35 (1).
19. Dorsal outline of the zygomatic process of the squamosal in lateral view ([24], modified): almost rectilinear (0); clearly dorsally convex forming a regular arc (1); dorsally convex with a with a abruptly ventral sloping in its posterior portion (2).
20. Articular rim on the lateral surface of the periotic ([92], modified): absent (0); present (1); present, forming a hook-like process (2).
21. Pars cochlearis of the periotic square-shaped in ventral view [92]: no (0); yes (1).
22. Aperture of the cochlear aqueduct of the periotic ([92], modified): small (0); very small (1); large and thin-edged (2).
23. Aperture of the cochlear aqueduct of the periotic ([92], modified): faces mediodorsally (0); faces dorsally (1).
24. Transverse thickening of the anterior process of the periotic [92]: no (0); yes (1).)
25. Internal auditory meatus of the periotic oval, with the dorsal opening for the facial canal lateral to the spiral cribriform tract [170]: no (0); yes (1).
26. Separate ossicle at the apex of the anterior process of the periotic [170]: no (0); yes (1).
27. Elongated anterior spine on the tympanic bulla, associated with a marked anterolateral convexity ([92], modified): no (0); moderate elongation (> 25% total length of the bulla) (1); extreme elongation (> 25% total length of the bulla) (2).
28. Ventral groove of the tympanic affecting the whole length of the bone, including the anterior spine [92]: no (0); yes (1).
29. Extent of the inner and outer posterior prominences of the tympanic [24]: both prominences with approximately the same posterior extent (0); outer posterior prominence posteriorly longer than the inner posterior prominence (1); outer posterior prominence posteriorly shorter than the inner posterior prominence (2).
30. Dorsal margin of the involucrum of the tympanic cut by a median indentation, in medial view [168]: absent (0), present (1).
31. Apical extension of the manubrium of the malleus [92]: no (0); yes (1).
32. Loss of double-rooted posterior teeth: [92]: no (0); yes (1).
33. Retention of accessory denticles on posterior teeth ([92], modified): yes (0); no (1).
34. Tooth count per upper or lower row ([24], modified): < 25 (0); > 25 and < 33 (1); > 33 (2).
35. Strong development of the dorsal transverse process of the atlas and extreme reduction of its ventral process [92]: no (0); yes (1).
36. Great reduction of coracoid process of the scapula [92]: no (0); yes (1).
37. Great reduction or loss of supraspinatus fossa, with acromion located on anterior edge of scapula [92]: no (0); yes (1).
38. Deep lateral groove on mandible [171]: no (0); yes (1).
39. Medial margin of the antorbital notch made of a thin plate [108]: (0) no, robust lateral margin of the rostrum at base; yes (1).
40. Dorsal surface of vertex [108]: flat (0); markedly transversely and longitudinally convex (1).
41. Vertex strongly transversely pinched [108]: absent (0); present, maxillae converging markedly posterior to bony nares (1).
42. Ventral edge of zygomatic process of squamosal in lateral view ([104], modified): concave (0); almost straight or convex (1).
43. Space between the apex of the zygomatic process of the squamosal and the posterior margin of the postorbital process of frontal in lateral view: zygomatic process distinctly posterior to the postorbital process and consequently there is space between the apex of the zygomatic process and the posterior margin of the postorbital process (0); apex of the zygomatic process exceeds the posterior margin of postorbital process and both processes are strictly in contact together (1).
44. Transverse width of the temporal fossae: temporal fossae wide transversally, ratio between the transverse width of the right + left temporal fossae and the BZW > 0.50 (0); temporal fossae moderately narrow transversally, ratio > 25 and < 50 (1); temporal fossae markedly narrow transversally < 25 (2).
45. Left-side torsion of the rostrum with longitudinal axis of the neurocranium forming an angle of about 5 degree with the main axis of the rostrum in dorsal view generating asymmetry of the posterior portion of the rostrum (right side transversally wider than the left side) and of the antorbital notches (left antorbital notch clearly more anteriorly placed and narrower than the right antorbital notch): absent(0); present (1).
46. Size of alveoli at the middle of the rostrum (greatest transverse diameter expressed as percentage of the BZW): > 3 % (0); <3 and >2 (1); < 2% (2).
47. Elongation of mandibular symphysis and angle between the mandibula rami; symphysis < 65 % of the total length of the mandibles and angle between the mandibles < 50 degrees (0); symphysis > 65 % of the total length of the mandibles and angle between the mandibles > 50 degrees (1).
48. Orientation of the head of the humerus: posteroproximally (0); posterolaterally (1).

## Appendix B

**Table A1:** Data matrix of 48 characters for one outgroup (*Zygorhiza*), 18 patanistoids and other possibly related odontocetes (*Squalodon*, *Waipatia* and the eurhinodelphinids *Eurhinodelphis*, *Xiphiacetus* and *Ziphiodelphis*). All characters are treated as unordered; 0, primitive state; 1, 2, derived states; a, variable between 0 and 1; b, variable between 1 and 2; ?, missing character.

	5	10	15	20	25	30	35	40	45	48
*Zygorhiza*	00000	00000	00000	00000	00000	00000	00000	000?0	?0000	000
*Squalodon*	00000	10000	00000	00010	00000	00000	00000	11000	00010	00?
*Waipatia*	00001	10000	00000	00000	00000	00000	00000	??000	00000	00?
*Xiphiacetus*	11101	00100	10000	000a0	00000	00011	?1020	00100	00110	20?
*Eurhinodelphis*	11101	00000	10001	00000	00000	00011	01?20	???00	00110	0??
*Ziphiodelphis*	11101	00100	10001	00000	00000	00011	01020	??100	00110	20?
*Zarhinocetus*	12100	10100	10010	0100?	00000	0???0	?1?2?	??100	10120	200
*Allodelphis*	12100	10000	10000	01001	10000	01020	?1?20	??a0	10120	200
*Ninjadelphis*	12??0	a1000	10000	0100?	10000	01020	?1?20	10100	10120	200
*Ensidelphis*	10011	0?1??	11?10	b111?	?????	?202?	?1?20	??110	01100	20?
MUSM 603	??011	00100	1??10	?1111	0??0?	02020	01???	???1?	01110	20?
*Macrosqualodelphis*	??011	00100	1?110	1111?	?????	?????	?1?01	???10	01101	0?1
*Huaridelphis*	00011	01100	11110	11111	12100	0???0	?1011	??000	01111	1??
*Notocetus*	00011	01100	11110	11111	12100	01100	11001	11000	01111	001
*Squalodelphis*	00012	11100	1?110	?11?1	12100	01100	1100?	??01?	0?11?	10?
*Phocageneus*	?????	?????	?????	????1	12100	01100	110?1	?????	??? ??	?0?
*Medocinia*	??012	11101	1?110	1111?	?????	?????	?1???	???00	011?1	???
*Furcacetus*	00011	0?100	11110	b1111	1??0?	0????	?1?0?	????0	0?111	1??
*Dilophodelphis*	00011	01101	11110	11111	1??0?	0????	?1?2?	???11	01111	2??
*Araeodelphis*	00001	00100	11110	???1?	?????	?????	?102?	??111	01110	21?
*Zarhachis*	12111	00111	11110	11122	00010	11010	01120	??111	01110	21?
*Pomatodelphis*	12111	00111	11110	11122	00010	01010	01120	??110	01110	21?
MUSM 1611	?????	?????	?????	????2	01001	1????	?????	?????	?????	???
*Platanista*	02111	00010	11110	21112	01001	11010	01110	11111	11100	210

## References

[1] Marx F.G., Lambert O., Uhen M.D. (2016). Cetacean Paleobiology.

[2] Fordyce R.E., Würsig B., Thewissen J.G.M., Kovacs K.M. (2018). Cetacean Evolution. Encyclopedia of Marine Mammals.

[3] de Muizon C. (2009). L’origine et l’histoire évolutive des Cétacés. Comptes Rendus Palevol.

[4] Gingerich P.D. (2012). Evolution of whales from land to sea. Proc. Am. Philos. Soc..

[5] Uhen M.D. (2010). The origin(s) of whales. Annu. Rev. Earth Planet. Sci..

[6] Thewissen J.G.M. (2014). The Walking Whales: From Land to Water in Eight Million Years.

[7] Marx F.G., Fordyce R.E. (2015). Baleen boom and bust: A synthesis of mysticete phylogeny, diversity and disparity. R. Soc. Open Sci..

[8] Tsai C.-H., Fordyce R.E. (2015). The earliest gulp-feeding mysticete (Cetacea: Mysticeti) from the Oligocene of New Zealand. J. Mamm. Evol..

[9] Geisler J.H., Boessenecker R.W., Brown M., Beatty B.L. (2017). The origin of filter feeding in whales. Curr. Biol..

[10] Lambert O., Martínez-Cáceres M., Bianucci G., Di Celma C., Salas-Gismondi R., Steurbaut E., Urbina M., de Muizon C. (2017). Earliest mysticete from the late Eocene of Peru sheds new light on the origin of baleen whales. Curr. Biol..

[11] Slater G.J., Goldbogen J.A., Pyenson N.D. (2017). Independent evolution of baleen whale gigantism linked to Plio-Pleistocene ocean dynamics. Proc. R. Soc. B Biol. Sci..

[12] Bianucci G., Marx F.G., Collareta A., Di Stefano A., Landini W., Morigi C., Varola A. (2019). Rise of the titans: Baleen whales became giants earlier than thought. Biol. Lett..

[13] Geisler J.H., Colbert M.W., Carew J.L. (2014). A new fossil species supports an early origin for toothed whale echolocation. Nature.

[14] Churchill M., Martinez-Caceres M., de Muizon C., Mnieckowski J., Geisler J.H. (2016). The origin of high-frequency hearing in whales. Curr. Biol..

[15] Park T., Fitzgerald E.M.G., Evans A.R. (2016). Ultrasonic hearing and echolocation in the earliest toothed whales. Biol. Lett..

[16] Boessenecker R.W., Fraser D., Churchill M., Geisler J.H. (2017). A toothless dwarf dolphin (Odontoceti: Xenorophidae) points to explosive feeding diversification of modern whales (Neoceti). Proc. R. Soc. B Biol. Sci..

[17] McCurry M.R., Pyenson N.D. (2019). Hyper-longirostry and kinematic disparity in extinct toothed whales. Paleobiology.

[18] Marx F.G., Uhen M.D. (2010). Climate, critters, and cetaceans: Cenozoic drivers of the evolution of modern whales. Science.

[19] Steeman M.E., Hebsgaard M.B., Fordyce R.E., Ho S.Y.W., Rabosky D.L., Nielsen R., Rahbek C., Glenner H., Sørensen M.V., Willerslev E. (2009). Radiation of extant cetaceans driven by restructuring of the oceans. Syst. Biol..

[20] Bianucci G., Collareta A., Bosio G., Landini W., Gariboldi K., Gioncada A., Lambert O., Malinverno E., de Muizon C., Varas-Malca R. (2018). Taphonomy and palaeoecology of the lower Miocene marine vertebrate assemblage of Ullujaya (Chilcatay Formation, East Pisco Basin, southern Peru). Palaeogeogr. Palaeoclimatol. Palaeoecol..

[21] Di Celma C., Malinverno E., Collareta A., Bosio G., Gariboldi K., Lambert O., Landini W., Pierantoni P.P., Gioncada A., Villa I.M. (2018). Facies analysis, stratigraphy and marine vertebrate assemblage of the lower Miocene Chilcatay Formation at Ullujaya (Pisco basin, Peru). J. Maps.

[22] Di Celma C., Pierantoni P.P., Malinverno E., Collareta A., Lambert O., Landini W., Bosio G., Gariboldi K., Gioncada A., de Muizon C. (2019). Allostratigraphy and paleontology of the lower Miocene Chilcatay Formation in the Zamaca area, East Pisco basin, southern Peru. J. Maps.

[23] Reeves R.R., Brownell H.R., Ridgway S.H., Harrison R.L.J. (1989). Susu *Platanista gangetica* (Roxburgh, 1801) and *Platanista minor* Owen, 1853. Handbook of Marine Mammals: River Dolphins and the Larger Toothed Whales.

[24] Lambert O., Bianucci G., Urbina M. (2014). *Huaridelphis raimondii*, a new early Miocene Squalodelphinidae (Cetacea, Odontoceti) from the Chilcatay Formation, Peru. J. Vertebr. Paleontol..

[25] Bianucci G., Urbina M., Lambert O. (2015). A new record of *Notocetus vanbenedeni* (Squalodelphinidae, Odontoceti, Cetacea) from the Early Miocene of Peru. Comptes Rendus Palevol.

[26] Bianucci G., Bosio G., Malinverno E., de Muizon C., Villa I.M., Urbina M., Lambert O. (2018). A new large squalodelphinid (Cetacea, Odontoceti) from Peru sheds light on the Early Miocene platanistoid disparity and ecology. R. Soc. Open Sci..

[27] Mead J.G., Fordyce R.E. (2009). The therian skull: A lexicon with emphasis on the odontocetes. Smithson. Contrib. Zool..

[28] Evans H.E., de Lahunta A. (2012). Miller’s Anatomy of the Dog.

[29] Swofford D.L. (2001). PAUP*. Phylogenetic Analysis Using Parsimony (*and Other Methods). Version 4b10.

[30] Thornburg T., Kulm L.D., Kulm L.D., Dymond J., Dasch E.J., Hussong D.M. (1981). Sedimentary basins of the Peru continental margin: Structure, stratigraphy, and Cenozoic tectonics from 6°S to 16°S latitude. Nazca Plate: Crustal Formation and Andean Convergence.

[31] Dunbar R.B., Marty R.C., Baker P.A. (1990). Cenozoic marine sedimentation in the Sechura and Pisco basins, Peru. Palaeogeogr. Palaeoclimatol. Palaeoecol..

[32] León Lecaros W.R., Rosell Solís W., Alemán R.A.M., Torres Bazán V.R.., De la Cruz Matos O. (2008). Estratigrafía, sedimentología y evolución tectónica de la cuenca Pisco Oriental. Bol. INGEMMET Ser. D.

[33] Cobbing E.J., Pitcher W.S. (1972). The coastal batholith of central Peru. J. Geol. Soc..

[34] Shackleton R.M., Ries A.C., Coward M.P., Cobbold P.R. (1979). Structure, metamorphism an geochronology of the Arequipa Massif of coastal Peru. J. Geol. Soc..

[35] Mukasa S.B., Henry D.J. (1990). The San Nicolás batholith of coastal Peru: Early Palaeozoic continental arc or continental rift magmatism?. J. Geol. Soc..

[36] DeVries T.J. (1998). Oligocene deposition and Cenozoic sequence boundaries in the Pisco Basin (Peru). J. S. Am. Earth Sci..

[37] DeVries T.J., Urbina M., Jud N.A. (2017). The Eocene-Oligocene Otuma depositional sequence (East Pisco Basin, Peru): Paleogeographic and paleoceanographic implications of new data. Bol. Soc. Geol. Perú.

[38] Di Celma C., Malinverno E., Bosio G., Collareta A., Gariboldi K., Gioncada A., Molli G., Basso D., Varas-Malca R.M., Pierantoni P.P. (2017). Sequence stratigraphy and paleontology of the upper Miocene Pisco Formation along the western side of the lower Ica Valley (Ica desert, Peru). Riv. Ital. Paleontol. Stratigr..

[39] DeVries T.J., Jud N.A. (2018). Lithofacies patterns and paleogeography of the Miocene Chilcatay and lower Pisco depositional sequences (East Pisco Basin, Peru). Bol. Soc. Geol. Perú.

[40] Brand L.R., Esperante R., Chadwick A.V., Porras O.P., Alomía M. (2004). Fossil whale preservation implies high diatom accumulation rate in the Miocene–Pliocene Pisco Formation of Peru. Geology.

[41] Esperante R., Brand L., Nick K.E., Poma O., Urbina M. (2008). Exceptional occurrence of fossil baleen in shallow marine sediments of the Neogene Pisco Formation, southern Peru. Palaeogeogr. Palaeoclimatol. Palaeoecol..

[42] Esperante R., Brand L.R., Chadwick A.V., Poma O. (2015). Taphonomy and paleoenvironmental conditions of deposition of fossil whales in the diatomaceous sediments of the Miocene/Pliocene Pisco Formation, southern Peru—A new fossil-lagerstätte. Palaeogeogr. Palaeoclimatol. Palaeoecol..

[43] Gariboldi K., Gioncada A., Bosio G., Malinverno E., Di Celma C., Tinelli C., Cantalamessa G., Landini W., Urbina M., Bianucci G. (2015). The dolomite nodules enclosing fossil marine vertebrates in the East Pisco Basin, Peru: Field and petrographic insights into the Lagerstätte formation. Palaeogeogr. Palaeoclimatol. Palaeoecol..

[44] Gioncada A., Collareta A., Gariboldi K., Lambert O., Di Celma C., Bonaccorsi E., Urbina M., Bianucci G. (2016). Inside baleen: Exceptional microstructure preservation in a late Miocene whale skeleton from Peru. Geology.

[45] Bianucci G., Di Celma C., Landini W., Post K., Tinelli C., de Muizon C., Gariboldi K., Malinverno E., Cantalamessa G., Gioncada A. (2016). Distribution of fossil marine vertebrates in Cerro Colorado, the type locality of the giant raptorial sperm whale *Livyatan melvillei* (Miocene, Pisco Formation, Peru). J. Maps.

[46] Bianucci G., Di Celma C., Collareta A., Landini W., Post K., Tinelli C., de Muizon C., Bosio G., Gariboldi K., Gioncada A. (2016). Fossil marine vertebrates of Cerro Los Quesos: Distribution of cetaceans, seals, crocodiles, seabirds, sharks, and bony fish in a late Miocene locality of the Pisco Basin, Peru. J. Maps.

[47] Uhen M.D., Pyenson N.D., Devries T.J., Urbina M., Renne P.R. (2011). New middle Eocene whales from the Pisco Basin of Peru. J. Paleontol..

[48] Martínez-Cáceres M., Lambert O., de Muizon C. (2017). The anatomy and phylogenetic affinities of *Cynthiacetus peruvianus*, a large *Dorudon*-like basilosaurid (Cetacea, Mammalia) from the late Eocene of Peru. Geodiversitas.

[49] Lambert O., Bianucci G., Salas-Gismondi R., Di Celma C., Steurbaut E., Urbina M., de Muizon C. (2019). An amphibious whale from the middle Eocene of Peru reveals early south pacific dispersal of quadrupedal cetaceans. Curr. Biol..

[50] de Muizon C. (1984). Les vertébrés fossiles de la Formation Pisco (Péru). Deuxième partie: Les Odontocetés (Cetacea, Mammalia) du Pliocène inférieur de Sud-Sacaco. Trav. Inst. Fr. Etud. And..

[51] de Muizon C. (1988). Les vertébrés fossiles de la Formation Pisco (Pérou). Troisième partie: Les odontocètes (Cetacea, Mammalia) Miocènes. Trav. Inst. Fr. Etud. And..

[52] de Muizon C. (1993). Walrus-like feeding adaptation in a new cetacean from the Pliocene of Peru. Nature.

[53] Lambert O., Bianucci G., Post K., de Muizon C., Salas-Gismondi R., Urbina M., Reumer J. (2010). The giant bite of a new raptorial sperm whale from the Miocene epoch of Peru. Nature.

[54] Lambert O., de Muizon C., Bianucci G. (2013). The most basal beaked whale *Ninoziphius platyrostris*, 1983: Clues on the evolutionary history of the family Ziphiidae (Cetacea: Odontoceti). Zool. J. Linn. Soc..

[55] Lambert O., Bianucci G., Beatty B.L. (2014). Bony outgrowths on the jaws of an extinct sperm whale support macroraptorial feeding in several stem physeteroids. Naturwissenschaften.

[56] Lambert O., Collareta A., Landini W., Post K., Ramassamy B., Di Celma C., Urbina M., Bianucci G. (2015). No deep diving: Evidence of predation on epipelagic fish for a stem beaked whale from the Late Miocene of Peru. Proc. R. Soc. B Biol. Sci..

[57] Lambert O., de Muizon C., Bianucci G. (2015). A new archaic homodont toothed cetacean (Mammalia, Cetacea, Odontoceti) from the early Miocene of Peru. Geodiversitas.

[58] Lambert O., Bianucci G., de Muizon C. (2016). Macroraptorial sperm whales (Cetacea, Odontoceti, Physeteroidea) from the Miocene of Peru. Zool. J. Linn. Soc..

[59] Lambert O., Bianucci G., Urbina M. (2017). A new inioid (Cetacea, Odontoceti, Delphinida) from the Miocene of Peru and the origin of modern dolphin and porpoise families. Zool. J. Linn. Soc..

[60] Lambert O., de Muizon C., Malinverno E., Celma C.D., Urbina M., Bianucci G. (2018). A new odontocete (toothed cetacean) from the Early Miocene of Peru expands the morphological disparity of extinct heterodont dolphins. J. Syst. Palaeontol..

[61] Bianucci G., Di Celma C., Urbina M., Lambert O. (2016). New beaked whales from the late Miocene of Peru and evidence for convergent evolution in stem and crown Ziphiidae (Cetacea, Odontoceti). PeerJ.

[62] Collareta A., Lambert O., de Muizon C., Urbina M., Bianucci G. (2017). *Koristocetus pescei* gen. et sp. nov., a diminutive sperm whale (Cetacea: Odontoceti: Kogiidae) from the late Miocene of Peru. Foss. Rec..

[63] Bouetel V., de Muizon C. (2006). The anatomy and relationships of *Piscobalaena nana* (Cetacea, Mysticeti), a Cetotheriidae s.s. from the early Pliocene of Peru. Geodiversitas.

[64] Collareta A., Landini W., Lambert O., Post K., Tinelli C., Di Celma C., Panetta D., Tripodi M., Salvadori P.A., Caramella D. (2015). Piscivory in a Miocene Cetotheriidae of Peru: First record of fossilized stomach content for an extinct baleen-bearing whale. Sci. Nat..

[65] Marx F.G., Kohno N. (2016). A new Miocene baleen whale from the Peruvian desert. R. Soc. Open Sci..

[66] Marx F.G., Lambert O., de Muizon C. (2017). A new Miocene baleen whale from Peru deciphers the dawn of cetotheriids. R. Soc. Open Sci..

[67] Marx F.G., Collareta A., Gioncada A., Post K., Lambert O., Bonaccorsi E., Urbina M., Bianucci G. (2017). How whales used to filter: Exceptionally preserved baleen in a Miocene cetotheriid. J. Anat..

[68] de Muizon C., Bianucci G., Martínez-Cáceres M., Lambert O. (2019). *Mystacodon selenensis*, the earliest known toothed mysticete (Cetacea, Mammalia) from the late Eocene of Peru. Geodiversitas.

[69] de Muizon C. (1981). Les vertébrés fossiles de la Formation Pisco (Pérou). Première partie: Deux nouveaux Monachinae (Phocidae, Mammalia) du Pliocène inférieur de Sud-Sacaco. Trav. Inst. Fr. Etud. And..

[70] Amson E., de Muizon C. (2014). A new durophagous phocid (Mammalia: Carnivora) from the late Neogene of Peru and considerations on monachine seals phylogeny. J. Syst. Palaeontol..

[71] Clarke J.A., Ksepka D.T., Stucchi M., Urbina M., Giannini N., Bertelli S., Narvaez Y., Boyd C.A. (2007). Paleogene equatorial penguins challenge the proposed relationship between biogeography, diversity, and Cenozoic climate change. Proc. Natl. Acad. Sci. USA.

[72] Clarke J.A., Ksepka D.T., Salas-Gismondi R., Altamirano A.J., Shawkey M.D., D’Alba L., Vinther J., DeVries T.J., Baby P. (2010). Fossil evidence for evolution of the shape and color of penguin feathers. Science.

[73] Ksepka D.T., Clarke J.A., DeVries T.J., Urbina M. (2008). Osteology of *Icadyptes salasi*, a giant penguin from the Eocene of Peru. J. Anat..

[74] Stucchi M., Varas-Malca R., Urbina-Schmitt M. (2016). New Miocene sulid birds from Peru and considerations on their Neogene fossil record in the Eastern Pacific Ocean. Acta Palaeontol. Pol..

[75] Parham J.F., Pyenson N.D. (2010). New sea turtle from the Miocene of Peru and the iterative evolution of feeding ecomorphologies since the Cretaceous. J. Paleontol..

[76] de Muizon C., McDonald H.G. (1995). An aquatic sloth from the Pliocene of Peru. Nature.

[77] de Muizon C., Mcdonald H.G., Salas R., Urbina M. (2003). A new early species of the aquatic sloth *Thalassocnus* (Mammalia, Xenarthra) from the Late Miocene of Peru. J. Vertebr. Paleontol..

[78] de Muizon C., Mcdonald H.G., Salas R., Urbina M. (2004). The youngest species of the aquatic sloth *Thalassocnus* and a reassessment of the relationships of the nothrothere sloths (Mammalia: Xenarthra). J. Vertebr. Paleontol..

[79] Amson E., Argot C., McDonald H.G., de Muizon C. (2015). Osteology and functional morphology of the forelimb of the marine sloth *Thalassocnus* (Mammalia, Tardigrada). J. Mamm. Evol..

[80] Amson E., Argot C., McDonald H.G., de Muizon C. (2015). Osteology and functional morphology of the hind limb of the marine sloth *Thalassocnus* (Mammalia, Tardigrada). J. Mamm. Evol..

[81] Amson E., Argot C., McDonald H.G., de Muizon C. (2015). Osteology and functional morphology of the axial postcranium of the marine sloth *Thalassocnus* (Mammalia, Tardigrada) with paleobiological implications. J. Mamm. Evol..

[82] Ehret D.J., Hubbell G., Macfadden B.J. (2009). Exceptional preservation of the white shark *Carcharodon* (Lamniformes, Lamnidae) from the early Pliocene of Peru. J. Vertebr. Paleontol..

[83] Ehret D.J., Macfadden B.J., Jones D.S., Devries T.J., Foster D.A., Salas-Gismondi R. (2012). Origin of the white shark *Carcharodon* (Lamniformes: Lamnidae) based on recalibration of the upper Neogene Pisco Formation of Peru. Palaeontology.

[84] Landini W., Altamirano-Sierra A., Collareta A., Di Celma C., Urbina M., Bianucci G. (2017). The late Miocene elasmobranch assemblage from Cerro Colorado (Pisco Formation, Peru). J. S. Am. Earth Sci..

[85] Landini W., Collareta A., Pesci F., Di Celma C., Urbina M., Bianucci G. (2017). A secondary nursery area for the copper shark *Carcharhinus brachyurus* from the late Miocene of Peru. J. S. Am. Earth Sci..

[86] Landini W., Collareta A., Di Celma C., Malinverno E., Urbina M., Bianucci G. (2019). The early Miocene elasmobranch assemblage from Zamaca (Chilcatay Formation, Peru). J. S. Am. Earth Sci..

[87] Collareta A., Lambert O., Landini W., Di Celma C., Malinverno E., Varas-Malca R., Urbina M., Bianucci G. (2017). Did the giant extinct shark *Carcharocles megalodon* target small prey? Bite marks on marine mammal remains from the late Miocene of Peru. Palaeogeogr. Palaeoclimatol. Palaeoecol..

[88] Collareta A., Landini W., Chacaltana C., Valdivia W., Altamirano-Sierra A., Urbina-Schmitt M., Bianucci G. (2017). A well preserved skeleton of the fossil shark *Cosmopolitodus hastalis* from the late Miocene of Peru, featuring fish remains as fossilized stomach contents. Riv. Ital. Paleontol. Stratigr..

[89] Bosio G., Gioncada A., Malinverno E., Di Celma C., Villa I.M., Cataldi G., Gariboldi K., Collareta A., Urbina M., Bianucci G. (2019). Chemical and petrographic fingerprinting of volcanic ashes as a tool for high-resolution stratigraphy of the upper Miocene Pisco Formation (Peru). J. Geol. Soc..

[90] Bosio G., Malinverno E., Collareta A., Di Celma C., Gioncada A., Parente M., Berra F., Marx F.G., Vertino A., Urbina M. (2020). Strontium Isotope Stratigraphy and the thermophilic fossil fauna from the middle Miocene of the East Pisco Basin (Peru). J. S. Am. Earth Sci..

[91] Simpson G.G. (1945). The principles of classification and a classification of mammals. Bull. Am. Mus. Nat. Hist..

[92] de Muizon C. (1987). The Affinities of *Notocetus vanbenedeni*, an Early Miocene platanistoid (Cetacea, Mammalia). Am. Mus. Novit..

[93] de Muizon C. (1988). Les relations phylogenetiques des Delphinida (Cetacea, Mammalia). Ann. Paléontol..

[94] Cassens I., Vicario S., Waddell V.G., Balchowsky H., Van Belle D., Ding W., Fan C., Mohan R.S.L., Simoes-Lopes P.C., Bastida R. (2000). Independent adaptation to riverine habitats allowed survival of ancient cetacean lineages. Proc. Natl. Acad. Sci. USA.

[95] Geisler J.H., McGowen M.R., Yang G., Gatesy J. (2011). A supermatrix analysis of genomic, morphological, and paleontological data from crown Cetacea. BMC Evol. Biol..

[96] de Muizon C., Lambert O., Bianucci G., Würsig B., Thewissen J.G.M., Kovacs K.M. (2018). River dolphins, evolution. Encyclopedia of Marine Mammals (Third Edition).

[97] Mcgowen M.R., Tsagkogeorga G., Álvarez-Carretero S., dos Reis M., Struebig M., Deaville R., Jepson P.D., Jarman S., Polanowski A., Morin P.A. (2019). Phylogenomic resolution of the cetacean tree of life using target sequence capture. Syst. Biol..

[98] de Muizon C. (1991). A new Ziphiidae (Cetacea) from the early Miocene of Washington State (USA) and phylogenetic analysis of the major groups of odontocetes. Bull. Muséum Natl. D’Histoire Nat. Paris.

[99] Fordyce R.E. (1994). *Waipatia maerewhenua*, new genus and new species (Waipatiidae, new family), an archaic late Oligocene dolphin (Cetacea: Odontoceti: Platanistoidea) from New Zealand. Proc. San Diego Soc. Nat. Hist..

[100] Barnes L.G. (2006). A phylogenetic analysis of the superfamily Platanistoidea (Mammalia, Cetacea, Odontoceti). Beitr. Zur Paläontol..

[101] Boersma A.T., McCurry M.R., Pyenson N.D. (2017). A new fossil dolphin *Dilophodelphis fordycei* provides insight into the evolution of supraorbital crests in Platanistoidea (Mammalia, Cetacea). R. Soc. Open Sci..

[102] Lambert O., Godfrey S.J., Fitzgerald E.M.G. (2018). *Yaquinacetus meadi*, a new latest Oligocene–early Miocene dolphin (Cetacea, Odontoceti, Squaloziphiidae, fam. nov.) from the Nye Mudstone (Oregon, U.S.A.). J. Vertebr. Paleontol..

[103] Tanaka Y., Fordyce R.E. (2014). Fossil Dolphin *Otekaikea marplesi* (Latest Oligocene, New Zealand) Expands the morphological and taxonomic diversity of Oligocene cetaceans. PLoS ONE.

[104] Tanaka Y., Fordyce R.E. (2015). A new Oligo-Miocene dolphin from New Zealand: *Otekaikea huata* expands diversity of the early Platanistoidea. Palaeontol. Electron..

[105] Tanaka Y., Fordyce R.E. (2017). *Awamokoa tokarahi*, a new basal dolphin in the Platanistoidea (late Oligocene, New Zealand). J. Syst. Palaeontol..

[106] Viglino M., Buono M., Gutstein C., Cozzuol M., Cuitiño J. (2018). A new dolphin from the early Miocene of Patagonia (Argentina): Insights into the evolution of Platanistoidea in the Southern Hemisphere. Acta Palaeontol. Pol..

[107] Viglino M., Buono M.R., Fordyce R.E., Cuitiño J.I., Fitzgerald E.M.G. (2019). Anatomy and phylogeny of the large shark-toothed dolphin *Phoberodon arctirostris* Cabrera, 1926 (Cetacea: Odontoceti) from the early Miocene of Patagonia (Argentina). Zool. J. Linn. Soc..

[108] Godfrey S.J., Barnes L.G., Lambert O. (2017). The early Miocene odontocete *Araeodelphis natator* Kellogg, 1957 (Cetacea; Platanistidae), from the Calvert Formation of Maryland, U.S.A.. J. Vertebr. Paleontol..

[109] Cantino P.D., de Queiroz K. PhyloCode. International Code of Phylogenetic Nomenclature. Version 4c. https://www.ohio.edu/phylocode/PhyloCode4c.pdf.

[110] Pyenson N.D., Sponberg S.N. (2011). Reconstructing body size in extinct crown Cetacea (Neoceti) using allometry, phylogenetic methods and tests from the fossil record. J. Mamm. Evol..

[111] Kellogg R. (1924). A fossil porpoise from the Calvert Formation of Maryland. Proc. U. S. Natl. Mus..

[112] Kellogg R. (1926). Supplementary observations on the skull of the fossil porpoise *Zarhachis flagellator* Cope. Proc. U. S. Natl. Mus..

[113] Smith B.D., Braulik G.T., Würsig B., Thewissen J.G.M., Kovacs K.M. (2018). Susu and Bhulan: *Platanista gangetica gangetica* and *P. g. minor*. Encyclopedia of Marine Mammals.

[114] Denuncio P., Panebianco M., Del Castillo D., Rodríguez D., Cappozzo H., Bastida R. (2016). Beak deviations in the skull of Franciscana dolphins *Pontoporia blainvillei* from Argentina. Dis. Aquat. Organ..

[115] Boersma A.T., Pyenson N.D. (2016). *Arktocara yakataga*, a new fossil odontocete (Mammalia, Cetacea) from the Oligocene of Alaska and the antiquity of Platanistoidea. PeerJ.

[116] Barnes L.G., Reynolds R.E. (2009). A new species of early Miocene allodelphinid dolphin (Cetacea, Odontoceti, Platanistoidea) from Cajon Pass, Southern California, U.S.A.. Mus. North. Ariz. Bull..

[117] Lambert O. (2005). Phylogenetic affinities of the long-snouted dolphin *Eurhinodelphis* (Cetacea, Odontoceti) from the Miocene of Antwerp. Palaeontology.

[118] Aguirre-Fernández G., Fordyce R.E. (2014). *Papahu taitapu*, gen. et sp. nov., an early Miocene stem odontocete (Cetacea) from New Zealand. J. Vertebr. Paleontol..

[119] Bianucci G., Miján I., Lambert O., Post K., Mateus O. (2013). Bizarre fossil beaked whales (Odontoceti, Ziphiidae) fished from the Atlantic Ocean floor off the Iberian Peninsula. Geodiversitas.

[120] von Schulte H.W., de Smith M.F. (1918). The external characters, skeletal muscles, and peripheral nerves of *Kogia breviceps* (Blainville). Bull. Am. Mus. Nat. Hist..

[121] Kimura T., Barnes L.G. (2016). New Miocene fossil Allodelphinidae (Cetacea, Odontoceti, Platanistoidea) from the North Pacific Ocean. Bull. Gunma Mus. Nat. Hist..

[122] Gutstein C.S., Cozzuol M.A., Vargas A.O., Suárez M.E., Schultz C.L., Rubilar-Rogers D. (2009). Patterns of skull variation of *Brachydelphis* (Cetacea, Odontoceti) from the Neogene of the southeastern Pacific. J. Mammal..

[123] Pilleri G., Gihr M. (1971). Differences observed in the skulls of *Platanista gangetica* (Roxburgh, 1801) and *Platanista indi* (Blyth, 1859). Investig. Cetacea.

[124] Montes I.D., Chavera C.A., Van Bresem M., Perales C.R., Falcón P.N., Van Waerebeek K. (2013). Descripción y evaluación anatómica de lesiones óseas cráneo-mandibulares en cetáceos odontocetos del Mar Peruano. Rev. Investig. Vet. Perú.

[125] True F.W. (1910). Description of a skull and some vertebrae of the fossil cetacean *Diochotichus vanbenedeni* from Santa Cruz, Patagonia. Bull. Am. Mus. Nat. Hist..

[126] Whitmore F.C., Kaltenbach A. (2008). Neogene Cetacea of the Lee Creek Phosphate Mine, North Carolina. Virginia Mus. Nat. Hist. Spec. Publ..

[127] Moreno F.P. (1892). Lijeros apuntes sobre dos generos de cetaceos fosiles de la Republica Argentina. Rev. Mus. Plata.

[128] Lydekker R. (1893). Contributions to the study of the fossil vertebrates of Argentina. I. The dinosaurs of Patagonia. An. Mus. Plata Paleontol..

[129] Cione A.L., Cozzuol M.A., Dozo M.T., Acosta Hospitaleche C. (2011). Marine vertebrate assemblages in the southwest Atlantic during the Miocene. Biol. J. Linn. Soc..

[130] Mchedlidze G.A. (1976). General Features of the Palaeobiological Evolution of Cetacea {Osnovnye Cherty Paleobiologicheskoi Istorii Kitoobraznykh}.

[131] Fitzgerald E.M.G. (2016). A late Oligocene waipatiid dolphin (Odontoceti: Waipatiidae) from Victoria, Australia. Mem. Mus. Vic..

[132] Kellogg R. (1957). Two additional Miocene porpoises from the Calvert Cliffs, Maryland. Proc. U. S. Natl. Mus..

[133] Kimura T. (2018). First squalodelphinid from the early Miocene of the Pacific realm in the Northern Hemisphere. J. Vertebr. Paleontol..

[134] Habegger M.L., Dean M.N., Dunlop J.W.C., Mullins G., Stokes M., Huber D.R., Winters D., Motta P.J. (2015). Feeding in billfishes: Inferring the role of the rostrum from a biomechanical standpoint. J. Exp. Biol..

[135] Layne L.N. (1959). Feeding adaptations and behavior of a freshwater dolphin, *Inia geoffrensis*. Anat. Rec..

[136] McHenry C.R., Clausen P.D., Daniel W.J.T., Meers M.B., Pendharkar A. (2006). Biomechanics of the rostrum in crocodilians: A comparative analysis using finite-element modeling. Anat. Rec. A Discov. Mol. Cell. Evol. Biol..

[137] McCurry M.R., Walmsley C.W., Fitzgerald E.M.G., McHenry C.R. (2017). The biomechanical consequences of longirostry in crocodilians and odontocetes. J. Biomech..

[138] Myrick A.C. (1979). Variation, Taphonomy and Adaptation of the Rhabdosteidae (=Eurhinodelphidae) (Odontoceti, Mammalia) from the Calvert Formation of Maryland and Virginia. Ph.D. Thesis.

[139] Cohen K.M., Finney S.C., Gibbard P.L., Fan J.-X. (2013). The ICS International Chronostratigraphic Chart. Episodes.

[140] Werth A.J. (2006). Mandibular and dental variation and the evolution of suction feeding in Odontoceti. J. Mammal..

[141] Pilleri G. (1970). Feeding behaviour of the Gangetic dolphin, *Platanista gangetica*, in captivity. Investig. Cetacea.

[142] Werth A., Schwenk K. (2000). Feeding in marine mammals. Feeding: Form, Function and Evolution in Tetrapod Vertebrates.

[143] Cranford T.W., Krysl P., Hildebrand J.A. (2008). Acoustic pathways revealed: Simulated sound transmission and reception in Cuvier’s beaked whale (*Ziphius cavirostris*). Bioinspir. Biomim..

[144] Casinos A., Ocaą J. (1979). A Craniometrical study of genus *Inia* D’Orbigny, 1834. Cetacea, Platanistoidea. Säugetierkd. Mitteilungen.

[145] Gerholdt J.M. (2006). Abnormalities and pathologies in the snout of the La Plata dolphin. Ecphora.

[146] Uhen M.D. (2004). Form, function, and anatomy of *Dorudon atrox* (Mammalia, Cetacea): An archaeocete from the middle to late Eocene of Egypt. Univ. Mich. Pap. Paleontol..

[147] Ramassamy B., Lambert O., Collareta A., Urbina M., Bianucci G. (2018). Description of the skeleton of the fossil beaked whale *Messapicetus gregarius*: Searching potential proxies for deep-diving abilities. Foss. Rec..

[148] Brownell R.L., Ridgway S.H., Harrison R.L.J. (1989). Franciscana *Pontoporia blainvillei* (Gervais and d’Orbigny, 1844). Handbook of Marine Mammals: River Dolphins and the Larger Toothed Whales.

[149] Lockyer C.H., Braulik G.T. (2014). An evaluation of age estimation using teeth from South Asian River dolphins (Platanistidae). Nammco Sci. Publ..

[150] Best R.C., da Silva M.F., Ridgway S.H., Harrison R.L.J., Ridgway S.H., Harrison R.L.J. (1989). Amazon river dolphin, boto *Inia geoffrensis* (de Blainville, 1817). Handbook of Marine Mammals: River Dolphins and the Larger Toothed Whales.

[151] Dal Piaz G. (1917). Gli Odontoceti del Miocene bellunese, Parte Terza. *Squalodelphis fabianii*. Mem. Ist. Geol. R. Univ. Padova.

[152] Collareta A., Di Cencio A., Ricci R., Bianucci G. (2020). The shark-toothed dolphin *Squalodon* (Cetacea: Odontoceti) from the remarkable Montagna della Majella marine vertebrate assemblage (Bolognano Formation, central Italy). Carnets Geol.

[153] Loch C., Kieser J.A., Fordyce R.E. (2015). Enamel ultrastructure in fossil cetaceans (Cetacea: Archaeoceti and Odontoceti). PLoS ONE.

[154] Kellogg R. (1927). *Kentriodon pernix*, a Miocene porpoise from Maryland. Proc. U. S. Natl. Mus..

[155] Cuff A.R., Rayfield E.J. (2013). Feeding mechanics in spinosaurid theropods and extant crocodilians. PLoS ONE.

[156] Vullo R., Allain R., Cavin L. (2016). Convergent evolution of jaws between spinosaurid dinosaurs and pike conger eels. Acta Palaeontol. Pol..

[157] Fahlke J.M., Gingerich P.D., Welsh R.C., Wood A.R. (2011). Cranial asymmetry in Eocene archaeocete whales and the evolution of directional hearing in water. Proc. Natl. Acad. Sci. USA.

[158] Herald E.S., Brownell R.L., Frye F.L., Morris E.J., Evans W., Scott A. (1969). Blind river dolphin: First side-swimming cetacean. Science.

[159] Macleod C.D., Reidenberg J.S., Weller M., Santos M.B., Herman J., Goold J., Pierce G.J. (2007). Breaking symmetry: The marine environment, prey size, and the evolution of asymmetry in cetacean skulls. Anat. Rec. Adv. Integr. Anat. Evol. Biol..

[160] Fahlke J.M., Hampe O. (2015). Cranial symmetry in baleen whales (Cetacea, Mysticeti) and the occurrence of cranial asymmetry throughout cetacean evolution. Sci. Nat..

[161] Hocking D.P., Marx F.G., Fitzgerald E.M.G., Evans A.R. (2017). Ancient whales did not filter feed with their teeth. Biol. Lett..

[162] Schäfer W. (1972). Ecology and Palaeoecology of Marine Environments.

[163] Mourlam M.J., Orliac M.J. (2017). Infrasonic and ultrasonic hearing evolved after the emergence of modern whales. Curr. Biol..

[164] Clementz M.T., Koch P.L. (2001). Differentiating aquatic mammal habitat and foraging ecology with stable isotopes in tooth enamel. Oecologia.

[165] Clementz M.T., Fordyce R.E., Peek S.L., Fox D.L. (2014). Ancient marine isoscapes and isotopic evidence of bulk-feeding by Oligocene cetaceans. Palaeogeogr. Palaeoclimatol. Palaeoecol..

[166] Marx F., Fitzgerald E., Fordyce R.E. (2019). Like phoenix from the ashes: How modern baleen whales arose from a fossil ‘dark age’. Acta Palaeontol. Pol..

[167] Bianucci G., Lambert O., Post K. (2010). High concentration of long-snouted beaked whales (genus *Messapicetus*) from the Miocene of Peru. Palaeontology.

[168] Lambert O. (2005). Systematics and phylogeny of the fossil beaked whales *Ziphirostrum* du Bus, 1868 and *Choneziphius* Duvernoy, 1851 (Mammalia, Cetacea, Odontoceti), from the Neogene of Antwerp (North of Belgium). Geodiversitas.

[169] Geisler J.H., Sanders A.E. (2003). Morphological evidence for the phylogeny of Cetacea. J. Mamm. Evol..

[170] Bianucci G., Lambert O., Salas-Gismondi R., Tejada J., Pujos F., Urbina M., Antoine P.-O. (2013). A Miocene relative of the Ganges River dolphin (Odontoceti, Platanistidae) from the Amazonian Basin. J. Vertebr. Paleontol..

[171] Miller G.S. (1923). Telescoping of the cetacean skull. Smithson. Misc. Collect..

